# Evolution of floral characters and biogeography of Heloniadeae (Melanthiaceae): an example of breeding system shifts with inflorescence change

**DOI:** 10.1038/s41598-021-01049-0

**Published:** 2021-11-02

**Authors:** Chien-Ti Chao, Chu-Chia Kuo, Jui-Tse Chang, Min-Wei Chai, Pei-Chun Liao

**Affiliations:** 1 School of Life Science, National Taiwan Normal University, No. 88, Tingchou Rd. Section 4, Wenshan District, Taipei City, 116 Taiwan; 2 Department of Biological Resources, National Chiayi University, No. 300, Hsuehfu Rd., Chiayi City, Chiayi County 600 Taiwan

**Keywords:** Plant ecology, Plant evolution

## Abstract

Heloniadeae (Melanthiaceae) presents an East Asia–North America disjunct distribution. Different molecular and morphological data nevertheless support the tribe as a monophyletic group. However, their phylogenetic relationships and biogeographic history, together with the character evolution, are not clear. Therefore, we constructed a Bayesian phylogenetic tree for Heloniadeae using cpDNA and inferred the historical biogeography and floral character evolution. The results revealed that Heloniadeae was distributed in high-latitudes of East Asia and North America, originating since 22.2 mya. The East Asia clade migrated into southwest China, and subsequently colonized the Korean Peninsula, Taiwan, the Ryukyus, and spread northward to Japan and southern Sakhalin. The evolution of the inflorescence and number of flowers were phylogenetically conserved, associated with the historical biogeography of Heloniadeae. The inflorescences transferred from raceme to sub-umbel, and the number of flowers decreased during the dispersal process, which may be accompanied by changes in the breeding system. Besides, the anthesis period was more affected by the habitat environment than phylogenetic constraints. The flowering temperature of Heloniadeae was below 20 °C in most species, except *H. kawanoi*. Such a low temperature might not be conductive to pollinator activities, but it could be compensated by sustaining seed production with long-lasting flowers.

## Introduction

Disjunct distribution is a discontinuous distribution pattern of organisms, and many types of disjunct distributions have been reported^1,2^. The intercontinental disjunction between North America and East Asia is one of the most prominent disjunctions, and had been long studied, over 150 years^3–12^. Till now, many taxa have been reported to display such a distribution pattern, such as *Sassafras*^13^, *Pogonia*^14^, *Chamaecyparis*^15^, *Maianthemum*^16^, *Cornus*^17^, *Pseudotsuga*^18^, and *Phryma*^19^. Generally, biogeographic studies attributed such distribution patterns to relics of Tertiary temperate forests, especially the disjunction between eastern North America and East Asia^20–24^.

Heloniadeae, a tribe of Melanthiaceae, comprises three genera which form the classic disjunct distribution between eastern North America (i.e., *Helonias* L.) and East Asia (i.e., *Heloniopsis* A.Gray, and *Ypsilandra* Franch)^25,26^. Among them, *Helonias* is a monotypic genus with *He. bullata* L. (*Helonias* was abbreviated as *He* after here) distributed in eastern North America^27^; in contrast, *Heloniopsis* has five species distributed in China, Japan, Korea, and Taiwan^28^; *Ypsilandra* has five species in China and the Himalayas^28^. These genera have perennial herbs with rosette leaves, scapose inflorescences, tepals with various colors, and seeds with caudate appendages on both ends. They also inhabit a similar habitat of the understory, shady and moist places, except that *Heloniopsis kawanoi* (Koidz.) Honda had been reported to also be epiphytic on trunks^29^. These genera are well known for their highly varying floral characters. The diagnostic characters for species delimitation rely on floral traits, such as inflorescence type, flower number per inflorescence, color of the tepals, stamen adnation, anther type, and style and stigma morphology^28,30,31^. *Helonias* has a compact raceme and three styluli. In contrast, *Heloniopsis* and *Ypsilandra* have relatively sparse racemes or umbels, and capitate or tri-lobed stigmas. The stamen of *Heloniopsis* is adnate to the opposite tepal, and the anther is dorsifixed with two locules or nearly so. On the other hand, the stamen of *Ypsilandra* is free from the tepals, and the anther is basifixed with one locule.

Previous studies on Heloniadeae were mainly focusing on taxonomy^30–37^, morphology^38–41^, life history^29,42^, genetic structure^43,44^, species maintenance mechanism^45^, and pollination biology^46,47^. The phylogeny of Heloniadeae has been reported in Tanaka^31,48^, Fuse and Tamura^45^, Fuse et al.^49,50^, and Kim et al.^26,51^. Among them, Fuse and Tamura^50^ reconstructed a phylogenetic tree from five plastid loci and discussed the generic classification of Heloniadeae. Although Tanaka^31,48^, Fuse et al.^49,50^, and Kim et al.^26^ discussed the biogeographic history, the results were merely based on morphological and phenetic aspects^31,39^, considered Heloniadeae lumped in the higher rank of Melanthiaceae^51^, or just had limited discussions^49^.

These studies have provided a wealth of data for the morphology and phylogeny of these genera, showing some trends in distribution and flower characteristics. For example, raceme inflorescence was mainly in continental species, e.g. *He. bullata*, *Ypsilandra*, and *H. koreana* and *H. tubiflora*, in contrast, the sub-umbel inflorescence was only found in the insular species of *Heloniopsis*. According to the phylogeny of Fuse and Tamura^45^ and the biogeographic inference of Tanaka^31^, the spreading of Heloniadeae was most probably from continental to the insular environment. Therefore, the inflorescence type change might be associated with biogeography. Based on these previous morphological and molecular findings, this study aims to reconstruct the historical biogeography of Heloniadeae from a phylogenetic perspective, focusing on the origin and spreading of *Heloniopsis*, by the model-based Bayesian evolutionary analysis. As a result, a hypothesis of the disjunct distribution was proposed, and the reconstruction of ancestral state and trait evolution, especially the floral parts, were also explored in this study.

## Results

### Phylogenetic analysis

**Figure 1:**
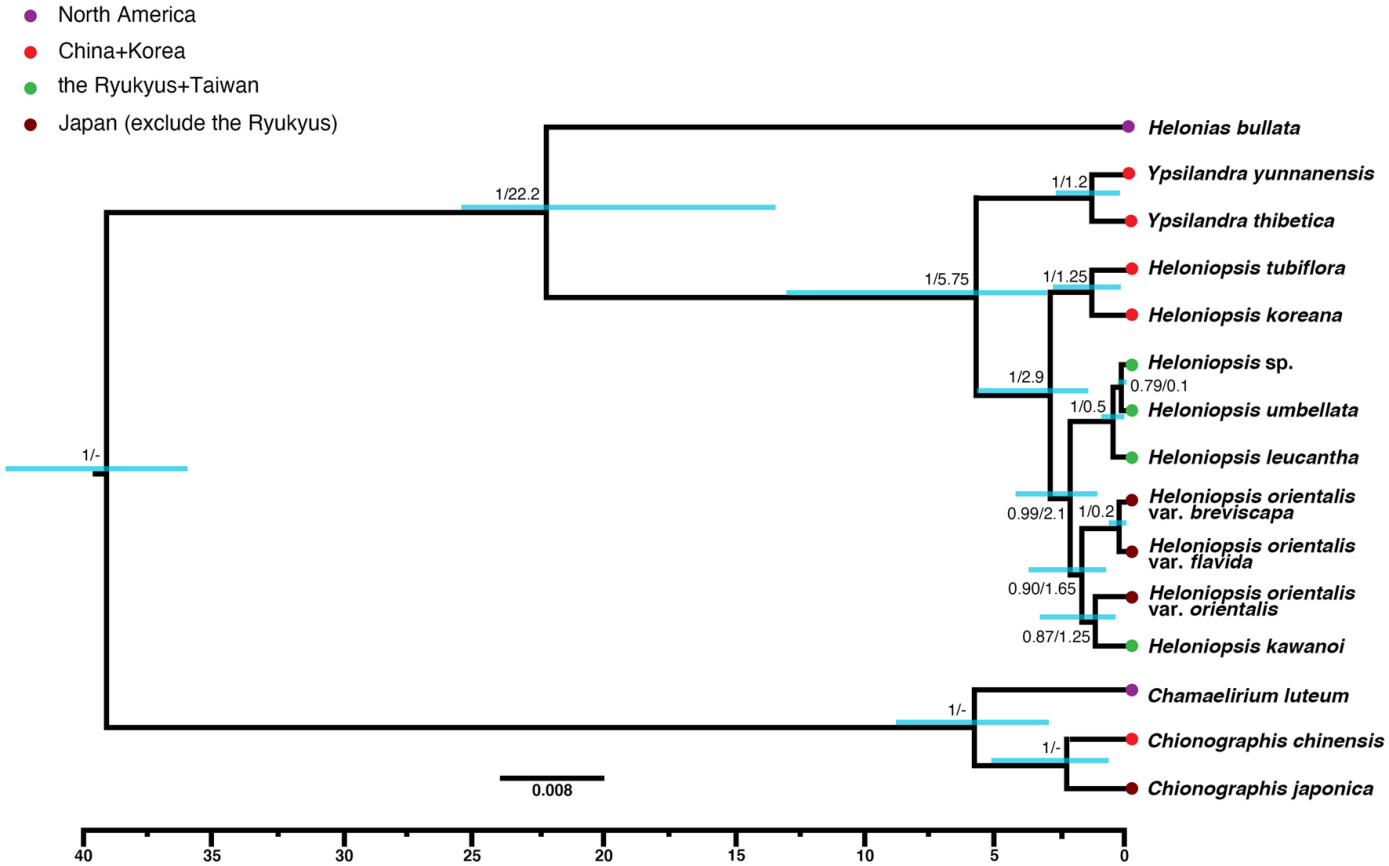
Phylogenetic tree of Heloniadeae reconstructed by chloroplast sequences. The value of each node represented supporting value (pp)/dating (mya), dating of outgroups not shown here. Bar on each node represent 95% HPD of dating results.

The substitution models selected for the phylogenetic analysis were HKY for atpB-rbcL and trnK, and HKY + G for the other sequences (Table 1). With *Chionographis* (*C. chinensis* and *C. japonica*) and *Chamaelirium luteum* as the outgroups, the phylogenetic tree confirms the monophyly of Heloniadeae that comprises a total of 11 taxa and one putative species from Taiwan (Fig. 1). In this phylogenetic tree, the genus *Helonias* diverged first from the monophyletic group of *Ypsilandra* and *Heloniopsis*. Each genus forms a highly supported monophyletic group [posterior probability (pp) = 1] (Fig. 1), as does the clade of two *Ypsilandra* species. *Heloniopsis* consisted of three highly to moderately supported clades. Among them, two Korean species (*H. koreana* Fuse, N.S.Lee & M.N.Tamura and *H. tubiflora* Fuse, N.S.Lee & M.N.Tamura) formed a highly supported clade (pp = 1) sister to the others. Among the remaining species, *H. umbellata*, *H. leucantha* (Koidz.) Honda, and *Heloniopsis* sp. formed a robust clade (pp = 1), and *H. leucantha* was sister to the others. The other taxa formed a highly supported clade (pp = 0.90), which consists of two subclades: one is comprised of two varieties of *H. orientalis* (Thunb.) Tanaka [var. *breviscapa* (Maxim.) Ohwi and var. *flavida* (Nakai) Ohwi], and another is composed of its autonym and *H. kawanoi*. However, the latter had only moderate support (pp = 0.87).

**Table 1:** Information of cpDNA dataset and primer used in the phylogenetic analysis.

Region	Length	VS	IS	Substitution model	primer	Sequence	References
*atp*B-*rbc*L	911	83	68	HKY	atpB-1 rbcL-1	5′-ACATCKARTACKGGACCAATAA-3′ 5′-AACACCAGCTTTRAATCCAA-3′	Chiang et al.^66^ Chiang et al.^66^
*mat*K	781	83	64	HKY + G	matK-F matK-R	5′-CTAATACCCTATCCCATCCATC-3′ 5′-CAAAGTTCTAGCACACGAAAGTC-3′	This study This study
*trn*G	648	52	36	HKY + G	5'trnG2G 3'trnG^UUC^	5′-GCGGGTATAGTTTAGTGGTAAAA-3′ 5′-GTAGCGGGAATCGAACCCGCATC-3′	Shaw et al.^67^ Shaw et al.^67^
*trn*K	584	59	37	HKY	trnK11 trnK630	5′-CTCAACGGTAGAGTACTCG-3′ 5′-ATCCTTTATTTTTGCAACCC-3′	Liston and Kadereit^68^ Fuse and Tamura^37^
*trn*L-F	393	50	35	HKY + G	trnL-c trnL-d	5′-CGAAATCGGTAGACGCTACG-3′ 5′-GGGGATAGAGGGACTTGAAC-3′	Taberlet et al.^69^ Taberlet et al.^69^
Total	3317	327		240			

*Length* aligned length of each loci, *VS* variable sites, *IS* informative sites.

### Miocene origin with vicariant diversification of Heloniadeae

The divergence time estimated in BEAST indicated an origin of Heloniadeae during the Miocene, 22.2 mya [95% highest posterior density (HPD): 29.5–11.45 mya]. The age of *Ypsilandra* was estimated as 5.75 mya (95% HPD 3.0–13.0 mya), and that of *Heloniopsis* was 2.9 mya (95% HPD 5.7–1.5 mya). The two Korean endemic species, *H. koreana* and *H. tubiflora*, can be dated to 1.25 mya (95% HPD 2.8–0.2 mya). The subtending Taiwan + Ryukyu clades diversified at 0.1 mya (95% HPD 0.25–0 mya) and 0.5 mya (95% HPD 1.3–0.05 mya), respectively. The clade of *H. orioentalis* + *H. kawanoi* dated to 1.65 mya (95% HPD 3.75–0.8 mya) (Fig. 1).

**Figure 2:**
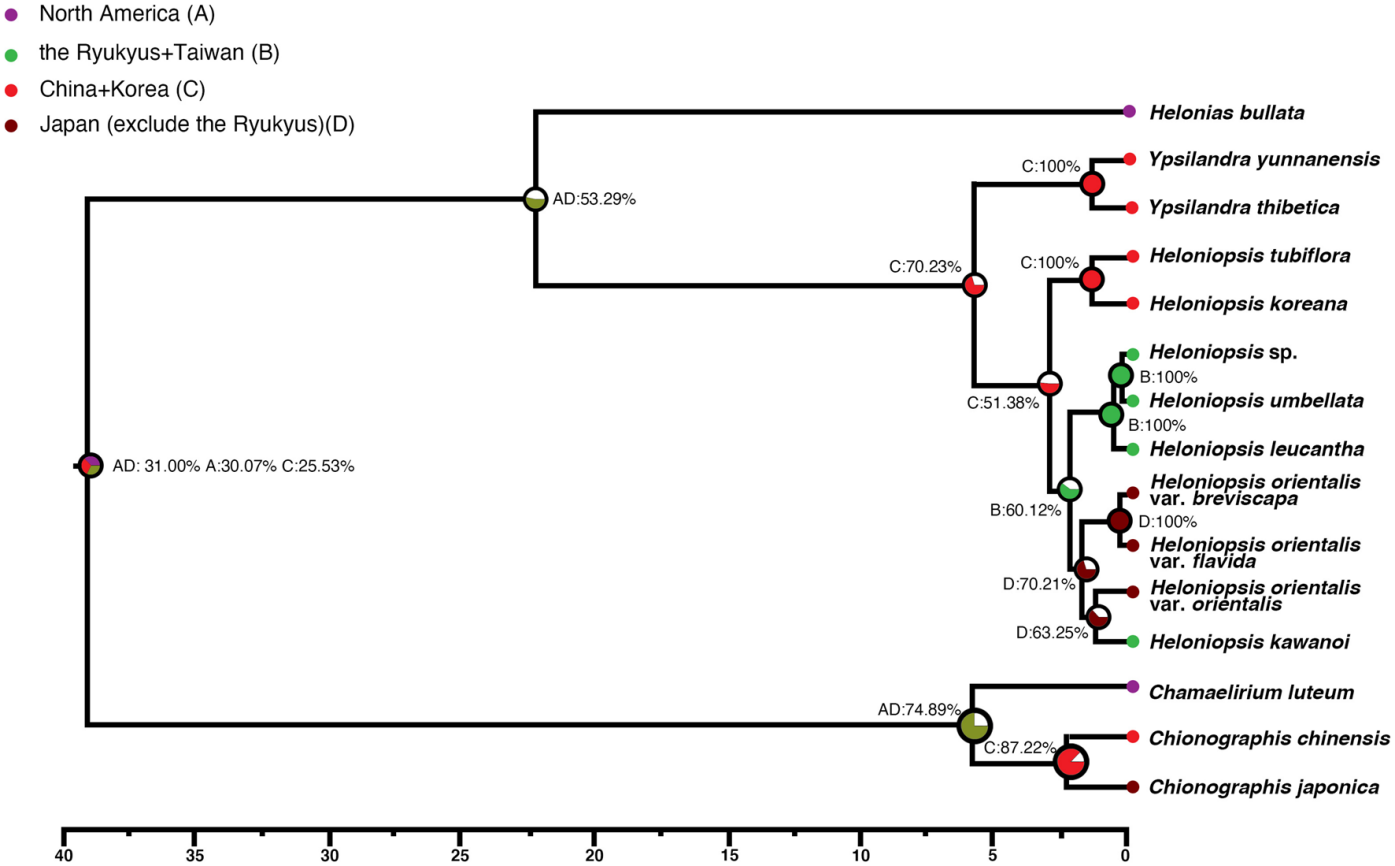
Ancestral area of Heloniadeae reconstructed by RASP with probability on each node, the color in the pie chart showing the ancestral area with over 50% probability.

**Figure 3:**
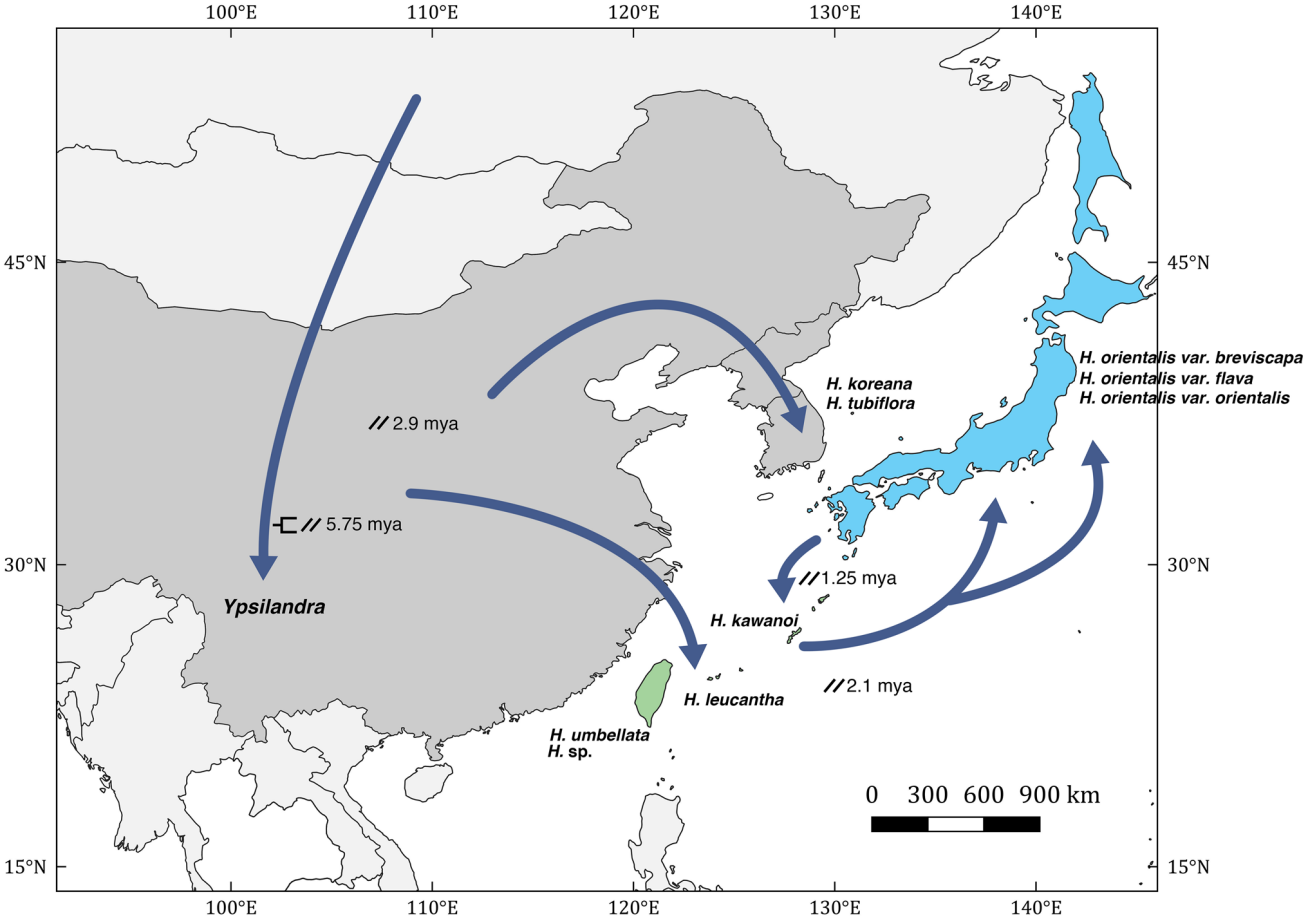
Dispersal routes of Heloniadeae in East Asia, with the dating of each event. Double slashes mean vicariance, and the clade symbol (besides *Ypsilandra*) means cladogenesis. The map was created using QGIS ver. 3.16 (https://www.qgis.org), and the map source was from GADM (https://gadm.org/index.html).

The best model for our dataset selected by BioGeoBears was DIVALIKE + J (AICc_wt = 0.51) (Supplementary Table S1). The ancestral area reconstruction suggests an ambiguous distribution for Heloniadeae, and the North America–East Asia disjunction might have been formed by a vicariance event (Fig. 2.) (Supplementary Tables S2, S3, Supplementary Fig. S4). The East Asia lineage experienced in situ cladogenesis and vicariance, resulting in two lineages, *Ypsilandra* and *Heloniopsis*. *Heloniopsis* further dispersed into the Ryukyus and Taiwan and separated into two lineages (viz. China + Korea and the Ryukyus + Taiwan) due to a vicariance event. The southern lineage of the Ryukyus + Taiwan further spread northward to Japan and became separate lineages in the two areas (Japan and the Ryukyus). The former one underwent in situ cladogenesis and vicariance resulting in the three present taxa. The latter one experienced cladogenesis and colonized southward to the Ryukyus again. After the vicariance event, it diversified to *H. orientalis* in Japan and *H. kawanoi* in the Ryukyus (Fig. 3).

### Ancestral state reconstruction of selected characters

**Figure 4:**
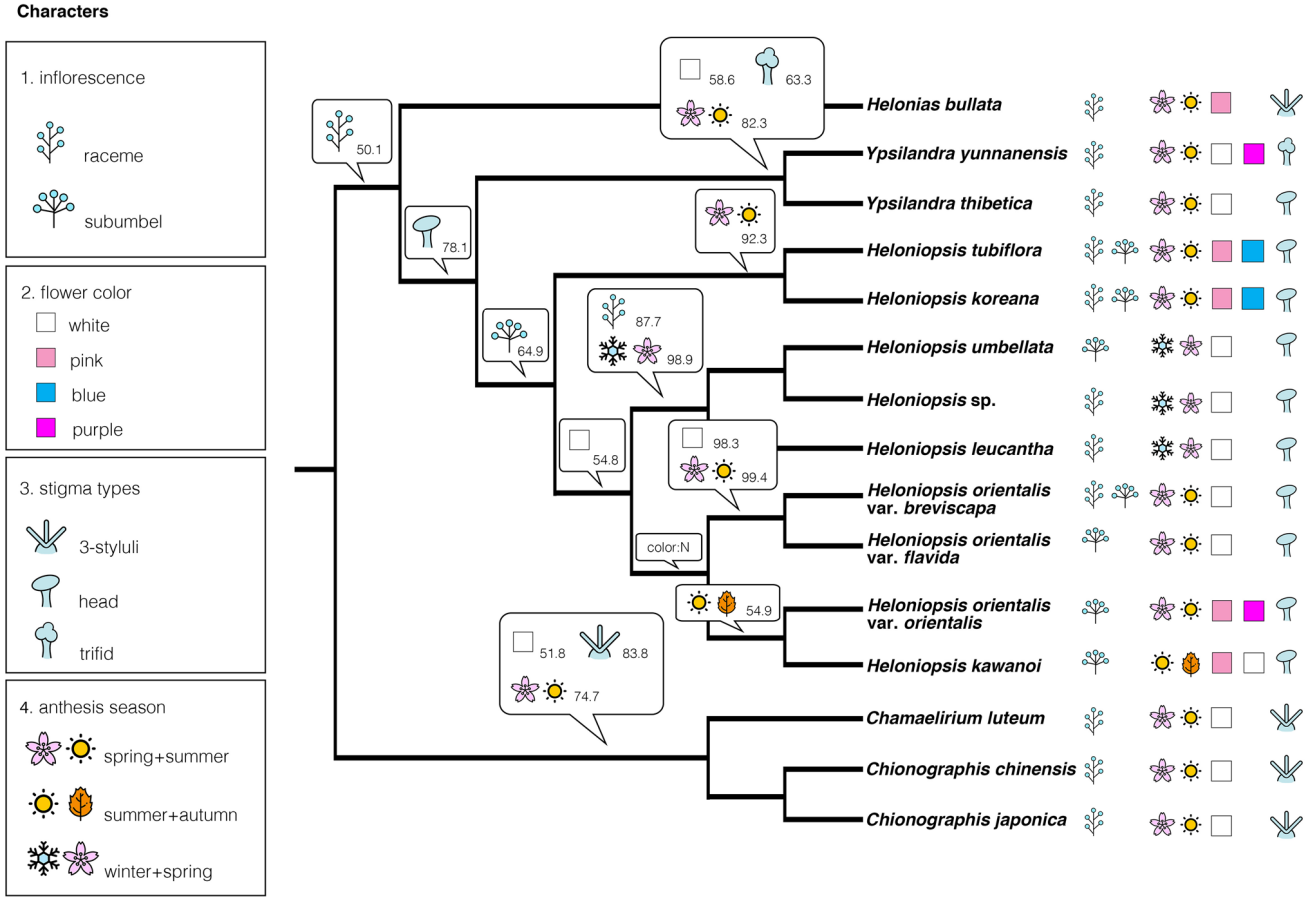
Evolution of changes in floral characters and anthesis period of Heloniadeae reconstructed by BayeTraits. Character states with probability > 50% were labelled on the node. Color: N represent the ancestral state was ambiguous on that node.

**Figure 5:**
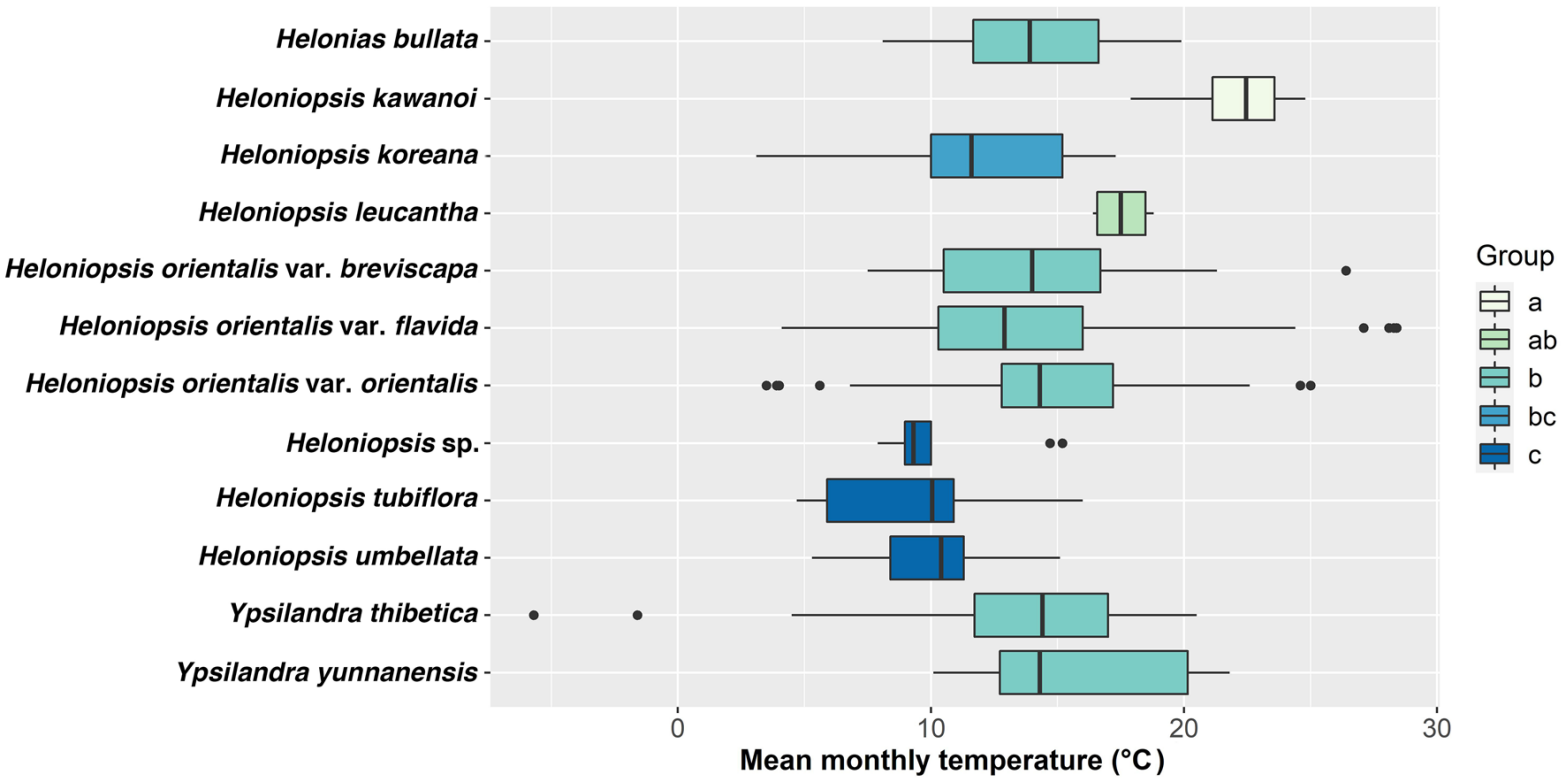
Boxplot of anthesis temperature analysis of Heloniadeae.

**Figure 6:**
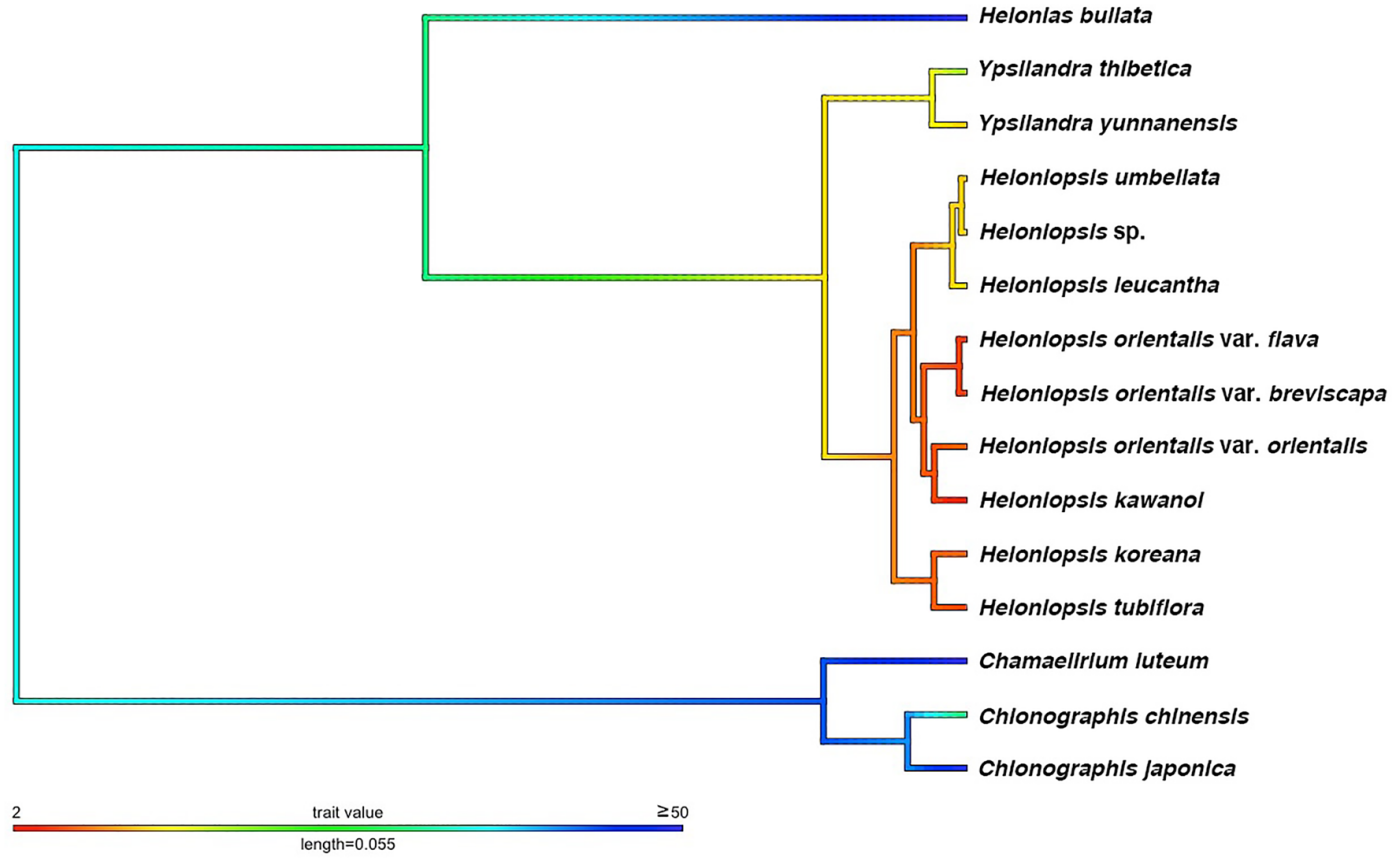
Evolution of number of flowers per inflorescence of Heloniadeae. Trait value represent flower number in an inflorescence.

Ancestral states of the floral and phenological characters were inferred on the reconstructed phylogeny. Different character revealed dissimilar state change patterns:

1. Inflorescence: The ancestral state of Heloniadeae and *Ypsilandra* + *Heloniopsis* was raceme, changing to sub-umbel in *Heloniopsis*. However, this state changed to raceme again in the Ryukyus + Taiwan and China + Korea clade (Fig. 4).
2. Flower color: The state change of this character was more complex and with more ambiguous states than in inflorescence. The ancestral state of flower color in Heloniadeae, *Ypsilandra* + *Heloniopsis*, and *Heloniopsis* was ambiguous. However, the ancestral state was white in the clade of Japan + the Ryukyus + Taiwan, and it was ambiguous in the Japan clade, and white in the clade of *H. orientalis* var. *flavida* + *H. orientalis* var. *breviscapa* (Fig. 4)*.*
3. Stigma: The ancestral state in *Ypsilandra* + *Heloniopsis* was reconstructed as capitate, and the 3-styluli stigma of *He. bullata* was an autapomorphy. The state of *Ypsilandra* was trifid, and all clades of *Heloniopsis* were reconstructed as capitate (Fig. 4).
4. Anthesis period: The ancestral state of Heloniadeae was spring to summer; however, this state became ambiguous in *Heloniopsis*. The state of the non-Korean species was reconstructed as summer to fall and switched to winter to spring in the Ryukyus + Taiwan clade, to spring to summer in the remaining species, and further reversely changed back to summer to fall in the clade of *H. kawanoi* + *H. orientalis* var. *orientalis* (Fig. 4).
5. Anthesis temperature: According to the suitable mean monthly temperature (mmt) of Heloniadeae, the species could be divided into three groups (Table 2, Fig. 5). The first group (group a) comprised only *H. kawanoi*, which the anthesis mmt was higher than 20 °C. In the second group, comprising *H. tubiflora*, *H. umbellata*, and *Heloniopsis* sp., the anthesis mmt was around or below 10 °C; the third group had an anthesis mmt between 10 and 20 °C, comprised *He. bullata*, *H. koreana, H. leucantha*, *H. orientalis* and its varieties, *Y. thibetica* Franch., and *Y. yunnanensis* W.W.Sm. & Jeffrey (Fig. 5).
6. Flower number: Flower number: The result showed reducing flower numbers in Heloniadeae over time. A reversal event was only observed in the clade of *H. leucantha* + *Heloniopsis* sp. + *H. umbellata* (Fig. 6).

**Table 2:** The result of ANOVA test for the flowering temperature of Heloniadeae.

	Df	Sum Sq	Mean Sq	F value	Pr(> F)
Name	11	358,170	32,561	24.07	< 2e−16***
Residuals	971	1,313,563	1353		

Signif. codes: 0 ‘***’0.001 ‘**’0.01 ‘*’0.05 ‘.’0.1 ‘ ’1.

### Correlation of inflorescence change and biogeography

The result of Pagel's test between inflorescence types and biogeography was marginal significant (For the sub-umbel and raceme coding in *H. koreana* and *H. tubiflora*, results were both marginally significant with *P*-value = 0.051 or 0.048, respectively) for alternative coding of inflorescence types.

## Discussion

In the study of Tanaka^31^, Heloniadeae was suggested as a member of the “Arcto-Tertiary Geoflora,” a group of deciduous broad-leaved plant communities in northern circumpolar regions^52,53^. The ancestor of Heloniadeae was widely distributed in the high latitudinal area of the Northern Hemisphere and dispersed southward to eastern Asia and eastern North America^31^. However, this inference was not well supported by the ambiguous inference of ancestral distribution of Heloniadeae in our BioGeoBEARS analysis. An analysis of plant taxa with the same (eastern North America–East Asia) distribution pattern revealed that all these taxa belonged to relic elements of temperate forest from the Tertiary^23^. Therefore, considering the Miocene origin and disjunct distribution pattern, we presumed that the tribe Heloniadeae might originate under the similar evolutionary pattern.

For the dating of Heloniadeae, Kim et al.^51^ suggested that the origin of Heloniadeae could be traced back to 27.3 mya, similar to our result (22.2 mya). Due to the lack of a fossil record in Melanthiaceae, Kim et al.^51^ calibrated the ages by applying fossil records of six outgroup clades. Here, we used a different strategy: an uncorrelated relaxed clock model but without fossil calibration but with an evolutionary rate of the chloroplast genes based on Wolfe et al.^54^. In our timeline, the divergence time between the North American and the Asian Heloniadeae is approximately Miocene (22.2 mya), which could be explained by the Beringia connection in the Miocene^55^. This divergence pattern was also found in some other species, e.g. *Phryma*^19^, *Pseudotsuga*^18^, *Meehania*^56^, and *Deutzia*^57^. The dating was similar to Kim et al.'s^51^ inference without ingroup fossils, and we expected the discovery of dated fossils in Heloniadeae or Melanthiaceae to validate this dating.

Two diversification hypotheses for the East Asian *Heloniopsis* have been proposed: one presumes that *Heloniopsis* originated in Sakhalin and spread southward into Japan and Korea^49^; the alternative presumed it originated in the southern part of East Asia, i.e., Taiwan and the Ryukyus, and migrated into Japan, Korea, and Sakhalin^31^. Generally, these two hypotheses are similar. They presume *Heloniopsis* originated on the islands of East Asia, spreading unidirectionally among these islands and the Korean peninsula. However, our result supports neither the inference of Fuse et al.^49^ nor Tanaka^31^. Our result shows that *Heloniopsis* possibly originated from southern China 5.75 mya, with a subsequent vicariance event (Fig. 2). Later, one lineage spread into the Korean Peninsula, and another colonized Taiwan and Ryukyus at approximately 2.9 mya. The colonization time roughly coincides with the formation of the islands of Taiwan and the Ryukyus^58–60^. After that, dispersal was northward to Japan and Sakhalin starting 1.65 mya, in the Pleistocene. If the Korean species spread from Sakhalin or the Ryukyus as proposed by Fuse et al.^49^ and Tanaka^31^, respectively, we would suppose that *Heloniopsis* inhabited areas surrounding the Korea Strait. However, populations of *Heloniopsis* are absent from Jeju Island, the southernmost part of the Korean peninsula, Shimayama island, and the Gotō archipelago. Besides, the dispersal of island *Heloniopsis* may not be unidirectional. Despite a general trend of dispersal from the southern (Taiwan + the Ryukyus) to the northern part (Sakhalin), one lineage had colonized the Ryukyus from major island of Japan, deriving *H. kawanoi* at approximately 1.25 mya.

States of most floral traits changed multiple times, including reversals, indicating a more complicated character evolution than Tanaka's^31^ inference. For the inflorescences, raceme was the primitive state, and sub-umbel was a derived one, and the flower number decreased from more than 50 flowers in *He. bullata* to the solitary flower of *H. kawanoi*, i.e. taxa with a sub-umbel usually had fewer flowers than those with a raceme. Tanaka^31^ supposed that the inflorescence change was pollinator- and habit-related, which improved the pollination efficiency. However, according to observations of the pollinator of *He. bullata*^61,62^ and *H. orientalis*^46^, the various flower visitors seem to be not diversified, and the flower morphology and the color under UV light also revealed a non-specialized pollination syndrome of Heloniadeae. Therefore, the shift in inflorescence type might not be attributed to the pollinator.

An extension of Baker's Law^63^ would be an alternative hypothesis for uniparental reproduction (self-compatibility) in plants on islands compared to their continental counterparts. The rationale is that the small population size after long-distance dispersal decreases the successful reproduction of obligate biparental populations, decreasing their fitness relative to uniparental ones at the beginning of colonization. However, many studies demonstrated the increasing dioecy on islands. This contrasting pattern was interpreted as in-situ diversification secondarily after colonization, when the selection against dioecy was relaxed through population expansion. As time goes by, Baker's Law would be obscured by subsequent local adaptation or genetic drift.

In terms of the breeding system, the dioecy–monoecy spectrum is connected with outcrossing and selfing, respectively. Besides dioecy and monoecy, floral display such as flower number and inflorescence are also related to the breeding system via pollination^64–68^. Several studies indicated that more flowers in an inflorescence could facilitate the mating opportunities; however, the self-pollination among different flowers in an inflorescence also increased, causing pollen discounting^66,69,70^. That is, the selfing rate would decrease by simplifying the inflorescence and reducing the flower number.

Among the Heloniadeae, Godt et al.^43^ found that the continental species *He. bullata* harbored low genetic variation due to frequent inbreeding; according to the results, a significant proportion of the seeds (20%) was produced in this way. A bagging experiment yielded seeds in 77% bagged flowers in this species^61^, revealing that autogamous pollination could occur under natural conditions. In contrast, a study of the island species, *H. orientalis* revealed a decreased selfing rate and increased outbreeding and total seed number with late flowering dates^47^. The bagging experiment with *H. orientalis* resulted in an extremely low seed: ovule ratio, revealing that autogamous pollination of this species was very limited^47^.

Tanaka^31^ also mentioned the change in the breeding system following the change in floral traits, especially the relative position between stamen and style. Based on Baker’s Law together with its connection with plant pollination and breeding, we hypothesize that the extant island species (i.e., *H. leucantha* and *H. umbellata*) with few but more aggregated flowers (i.e., sub-umbel) were evolved secondarily from their ancestors with abundant but more dispersed flowers (i.e., raceme). The more aggregated and decreased number of flowers would more easily attract pollinators and avoid inbreeding, outcompeting the early colonizing ancestors postceding the relaxation of selection against outcrossing.

Considerable flower-color variation is the characteristic of Heloniadeae^31,50^. However, the evolution of the flower color of Heloniadeae was less discussed. Here we show that the probability of each flower-color state was less than 50% on all nodes, revealing ambiguous patterns of ancestral states (Fig. 5). Only in the Ryukyu + Taiwan clade (*H. leucantha*, *H. umbellata*, and *Heloniopsis* sp.) and the clade composed of *H. orientalis* var. *flavida* and *H. orientalis* var. *orientalis* are dominated in white. All parts of the flowers of *H. orientalis* showed strong UV absorption without special patterns for insect vision^46^. The flower color variation of Heloniadeae might not act as nectary guilds like some other species^71,72^. Alternatively, it might provide a corresponding light color visual attraction to the pollinators under the dark understory environment^29^. Therefore, the flower color variation might be more influenced by habitat environments than by phylogenetic constraints. Further studies on the pollination of Heloniadeae were necessary to elucidate the flower color and pollinator interaction.

Compared to the other characters, the change of stigma states was relatively simple, both for Heloniadeae and *Heloniopsis*. The ancestral state of these taxa is capitate, whereas the tri-lobed one is a derived state. Except for the case of *Y. thibetica*, the only *Ypsilandra* species bearing a capitate or discoid stigma^31^, there was consensus about the stigma type within each genus. Although the evolution of floral organs is often tied with pollination syndrome, this seems not to be the case for the stigma of Heloniadeae.

The anthesis period reflects the flowering schedule, in which three anthesis periods were defined, viz. late spring to summer, late summer to autumn, and late winter to spring. The first period was suggested as the primitive state in Heloniadeae in this study, and the other two periods were derived ones. The spring–summer species (Heloniadeae excluding *H. leucantha*, *H. kawanoi*, *H. umbellata*, and *Heloniopsis* sp.) were all distributed in temperate or high-altitude areas; in contrast, the others are in the subtropics. Therefore, the evolution of this character might be related to adaptation to the local climate instead of phylogenetic constraints. The late winter to summer anthesis period of most Heloniadeae taxa implied most members were vernal flowering species, and the flowering phenology was susceptible to temperature change^73,74^.

Although there might be geographical bias caused by the rare collection record of some species, the analysis still revealed the anthesis temperature pattern of Heloniadeae. Our analysis of the mmt of the anthesis period indicated all species except *H. kawanoi* had an anthesis mmt below 20 °C. Furthermore, species could be classified into three groups according to their mmt, and the mmt of most temperate species were between 10 and 20 °C. For *H. tubiflora* and *H. koreana*, these closely related species are endemic to the Korean peninsula and distributed sympatrically, but have a different anthesis mmt. Such differences in phenological characteristics might affect pollinator behavior and hence maintain reproductive isolation. However, the anthesis mmt of the subtropical species, viz. *H. kawanoi*, *H. leucantha*, *H. umbellata*, and *Heloniopsis* sp., classified them into different groups. In the monophyletic group of *H. leucantha*, *H. umbellata*, and *Heloniopsis* sp., mmt of *H. leucantha* was 15–20 °C, and that of the others was around 10 °C. Such differences might be due to the distributional differences in altitude: *H. leucantha* is distributed in the Ryukyus, while *H. umbellata* and *Heloniopsis* sp. are endemic to the medium-altitude mountains (1500–2000 m) in Taiwan. Lower anthesis temperature was unfavorable for the pollinator. However, the long anthesis period in each flower (7–14 days)^47^, and the aggregated flowers, light flower color, with unspecialized pollinators still improved the pollination efficiency and resulted in mass production of seeds.

This study formulates an evolutionary hypothesis for the origin and dispersal of Heloniadeae from molecular data. The migration route of this tribe in East Asia was generally from continental to insular areas and then from south to north. Simultaneously, the inflorescence type became shorter and the number of flowers decreased during the dispersal process of *Heloniopsis* from North America to the continent and islands of East Asia, and accompanying the breeding system alteration. Along with the changes in the number of flowers, the evolutionary trend of these floral structures guarantees effective sexual reproduction. Our study provides a detailed biogeographic inference of Heloniadeae in East Asia and proposes a new model for the anthesis phenology of vernal species.

## Methods

### Source of materials

In this study, we integrated previous studies on Heloniadeae to analyze of the historical biogeography and character evolution. Therefore, we gathered morphological data from Chen et al.^28^, Tanaka^31,75^, Utech^27^, Fuse et al.^49^, Hsu et al.^76^, and Fuse^30^, and by visual examination of herbarium sheets or via online websites. The following herbaria or their websites were examined: HAST, KYO, P, TAI, TAIF, TCF, TNM, herbarium acronyms according to Thiers^77^. Over 100 herbarium sheets were examined for this study.

All taxa of Heloniadeae were included in this study; for the scientific names of all taxa Fuse and Tamura^50^ was followed. *Chamaelirium luteum*, *Chionographis chinensis*, and *Chionographis japonica* were selected as outgroups following the studies of Kim et al.^26^, and Fuse and Tamura^50^. Most sequences used in this study were adopted from Fuse and Tamura^50^ and downloaded from NCBI databases.

For new sequencing, young leaves of *H. umbellata* and *Heloniopsis* sp. were collected from Taiwan. Leaves were preserved in silica gel for DNA extraction. The newly collected taxa were not listed as protected species in the Cultural Heritage Preservation Act, the plant protection law in Taiwan. The collection location was neither the protected and reserved areas nor private land. No permission was required for the new collection of *H. umbellata* and *Heloniopsis* sp. The first author (C.T.C.) undertook formal identification of these taxa. Collection information for these materials is listed in Table 3. Voucher specimens were deposited in the herbarium of the Taiwan Forestry Research Institute (TAIF).

**Table 3:** Collection information of *Heloniopsis* taxa for newly sequencing.

Taxa	Locality	Coll. no
*Heloniopsis umbellata*	TAIWAN, Taoyuan City, Fuhsing District, near the crossroad of county road Bei113 and Tao119	Chao 4746
*Heloniopsis* sp.	TAIWAN, Nantou County, Chushan Town, Sun-link-sea vacation resorts, Sunglungyen	Chao 4742
	TAIWAN, Nantou County, Luku Town, industrial road to Chugaowan tea garden	Chao 4743

### DNA extraction and PCR protocols

Samples of *H. umbellata* and *Heloniopsis* sp. from Taiwan were included here. Accession numbers of all sequences are listed in Supplementary Table S5. Total genomic DNA was extracted by the modified CTAB method^78^. Five cpDNA fragments (atpB-rbcL, matK, trnG, trnK, and trnL-F) were applied for phylogeny reconstruction. The primers of all cpDNA loci used for the polymerase chain reaction (PCR) are listed in Table 1^79–82^. PCR amplification was conducted by the following protocol: 3 min at 94 °C for enzyme activation, and 35 cycles of 94 °C for 30 s, 57 °C for 30 s, and 72 °C for 1 min, followed by a final extension at 72 °C for 5 min. Quality and quantity of the PCR products were checked by gel electrophoresis and then they were sequenced using an ABI PRISMH3730XL DNA Sequencer (Perkin-Elmer, Foster City, CA, USA). All experiments were performed following the relevant guidelines and regulations.

### Phylogeny reconstruction

The sequences (including the downloaded and newly sequenced ones) were aligned in BioEdit 7.2.5^83^. Substitution models for the five cpDNA fragments were selected with MEGA 7.0^84^; the models with the lowest Akaike and Bayesian Information Criterion (AIC and BIC) values were applied in the following analyses.

The five chloroplast fragments, viz. atpB-rbcL, matK, trnG, trnK, and trnL-F, were concatenated to reconstruct the phylogenetic tree. The phylogenetic tree was reconstructed using BEAST v.1.8.0^85^. The *xml* file was generated in BEAUti v.1.8.0^85^, and the substitution models selected as mentioned above were applied. The clock model was set as a strict clock with a constant rate of 2 × 10^−9^ per site per year^54^. The length of the MCMC chain reaction was set as 5 × 10^8^ generations sampled every 50,000 generations; thus, a total of 10,000 trees were kept. Tracer v.1.6^86^ was used to check that the values of mean and ESS in the log file were over 200. TreeAnnotator v.1.8.0^85^ was applied to construct the consensus tree and it was visualized using FigTree v.1.4.0^87^.

### Character coding

For the biogeographic and character evolutionary analysis, distribution and selected morphological characters were coded manually. The distributional range was divided into four areas, namely North America (A), Taiwan and the Ryukyus (B), Korea and China (C), and Japan (excluding the Ryukyus) (D). These areas were selected according to the patterns of endemism and the distribution of each taxon.

Regarding the morphological analysis, we mainly chose characters, especially the floral parts, that were commonly used in the species diagnosis of Heloniadeae. Character states of each taxon were determined from herbarium specimens, living plants, and the studies of Hsu et al.^76^, Tanaka^31,75^, Utech^27^, Fuse et al.^49^, and Fuse^30^. The following characters were applied in this study: inflorescence type, flower color, anthesis period, flower number, and stigma type. The final list of characters is presented in Supplementary Table S1, together with their distribution. The anthesis period was classified into three categories according to the season of anthesis, namely spring to summer, summer to autumn, and winter to spring.

### Biogeographic inference

RASP 4.2^88^ was used for historical biogeographic inference. The biogeographic models, including jump dispersal events (+ J), were selected using the R package BioGeoBEARS^89^. The maximum distribution range was set to two areas, and the following combination of areas was excluded from the analysis, i.e. North America + Taiwan + Ryukyus (AB). The model with the highest AICc_wt was regarded as the best one for our dataset (Supplementary Table S6).

### Character evolution

Ancestral states of discrete characters were inferred with BayesTraits^90^ of RASP 4.2^88^ with default settings. The character with a possibility higher than 50% was labeled on the clade. Because non-discrete data cannot be applied in BayesTraits, the ancestral state of flower number was determined using the package *phytools* of R^91,92^.

In order to test the correlation between inflorescence change and biogeographic pattern, we used the Pagel's test of package *phytools* of R^91,92^. However, because only binary character state data could be tested in Pagel's test, we re-coded each taxon's distribution and inflorescence. For the distribution, we re-coded the states as continental and insular distribution; the inflorescence types were coded as raceme and sub-umbel. Some taxa (e.g., *H. koreana* and *H. tubiflora*) with both inflorescences were tested in alternative codings reciprocally.

### Anthesis temperature analysis

One of the characters we were interested in was the anthesis period. The records revealed that most Heloniadeae taxa bloom between late winter and early summer and are typical cases of vernal flowering plants. The anthesis of these species is often related to vernalization^73,93^. Thus, we collected and organized the distribution and flowering period of each species of Heloniadeae from the Global Biodiversity Information Facility (GBIF), iNaturalist (www.inaturalist.org), naturing.net (www.naturing.net), science museum net (science-net.kahaku.go.jp), and related references^28,30,49,50^. Further, we used the distribution points and the CHELSA Timeseries data, a high-resolution (~ 1 km) climate database for 1979–2013^94,95^, to extract the long-term mean monthly temperature of habitats during each species' flowering period. One-way ANOVA was used to test the similarity of the mean monthly temperature of each species' flowering period, and the Scheffe test was employed to determine significant differences (α = 0.05) among groups. To avoid bias caused by outliers, the first and third quantiles were used to describe the suitable anthesis temperature of each species. The analyzed location and their mean monthly temperature were provided in Supplementary Tables S7, S8, and S9.

## Supplementary Information

Supplementary Information.
