# Author Correction: Evolution of floral characters and biogeography of Heloniadeae (Melanthiaceae): an example of breeding system shifts with inflorescence change

**DOI:** 10.1038/s41598-022-05908-2

**Published:** 2022-01-27

**Authors:** Chien‑Ti Chao, Chu‑Chia Kuo, Jui‑Tse Chang, Min‑Wei Chai, Pei‑Chun Liao

**Affiliations:** 1 School of Life Science, National Taiwan Normal University, No. 88, Tingchou Rd. Section 4, Wenshan District, Taipei City, 116 Taiwan; 2 Department of Biological Resources, National Chiayi University, No. 300, Hsuehfu Rd., Chiayi City, Chiayi County 600 Taiwan

Correction to: *Scientific Reports* 10.1038/s41598-021-01049-0, published online 02 November 2021

The original version of this Article contained an error in the Abstract.

“The flowering temperature of was below 20 °C in most species, except *H. kawanoi*.”

now reads:

“The flowering temperature of Heloniadeae was below 20 °C in most species, except *H. kawanoi*.”

The original Article has been corrected.

