# Supplementary material for: Evolution of floral characters and biogeography of Heloniadeae (Melanthiaceae): an example of breeding system shifts with inflorescence change
Source: Sci Rep. 2021 Nov 2;11:21494. doi: 10.1038/s41598-021-01049-0 (PMC8563777; doi:10.1038/s41598-021-01049-0)
Supplement: Supplementary file 1 — Supplementary Information. [file 41598_2021_1049_MOESM1_ESM.pdf]

Supplementary Table S1. Coding of floral characters and distribution of Heloniadeae

	Distribution	inflorescence type	color of flower	flowering period	flower no.	stigma
Helonias bullata	North America	raceme	pink	late spring to early summer	30-70	3-styluli
Heloniopsis kawanoi	Ryukyu	solitary or subumbel	white or pink	aug-nov	1-3	capitate
Heloniopsis koreana	Korea	raceme or subumbel	blue or pink	apr-may	3-10	capitate
Heloniopsis leucantha	Ryukyu	raceme	white	jan-Feb	4-22	capitate
Heloniopsis orientalis var. orientalis	Hokkaido, Honsyu, Shikoku	subumbel	pink or purple	mar-jul	2-11	capitate
Heloniopsis orientalis var. flavida	Honsyu and Shikoku	subumbel	white	mar-apr	1-8	capitate
Heloniopsis orientalis var. breviscapa	Honsyu, Shikoku and Kyushu	raceme or subumbel	white	mar-apr	1-9	capitate
Heloniopsis tubiflora	Korea	raceme or subumbel	blue or pink	apr-may	3-7	capitate
Heloniopsis umbellata	Taiwan	subumbel	white	jan-feb	2-20	capitate
Heloniopsis sp.	Taiwan	raceme	white	jan-feb	4-20	capitate
Ypsilandra yunnanensis	China	raceme	white or purple	jun-jul	5-17	trifid
Ypsilandra thibetica	China	raceme	white	mar-apr	5-30	capitate
Chamaelirium luteum	North America	raceme	white	late spring to summer	>50	3-styluli
Chionographis chinensis	China	spike	white	apr-may	>50	3-styluli
Chionographis japonica	Japan, Korea	spike	white	may-jun	>50	3-styluli

	Distribution	inflorescence type	color of flower	fowering period	flower no. mean	stigma
Helonias bullata	A	A	A	A	50	C
Heloniopsis kawanoi	B	b	AB	C	2	B
Heloniopsis koreana	C	Ab	AC	A	6.5	B
Heloniopsis leucantha	B	A	B	B	13	B
Heloniopsis orientalis var. orientalis	D	b	AD	A	6.5	B
Heloniopsis orientalis var. flavida	D	b	B	A	4.5	B
Heloniopsis orientalis var. breviscapa	D	Ab	B	A	5	B
Heloniopsis tubiflora	C	Ab	AC	A	5	B
Heloniopsis umbellata	B	b	B	B	11	B
Heloniopsis sp.	B	A	B	B	12	B
Ypsilandra yunnanensis	C	A	BD	A	11	A
Ypsilandra thibetica	C	A	B	A	17.5	B
Chamaelirium luteum	A	A	B	A	50	C
Chionographis chinensis	C	a	B	A	50	C
Chionographis japonica	CD	a	B		50	C

	A North America	A raceme+spike	A pink	A spring-summer		A trifid
	B Taiwan+the Ryukyus	B subumbel+solitary	B white	B winter-spring		B capitate
	C China+Korea		C blue	C summer-fall		C 3-styluli
	D Japan (except the Ryukyus)		D purple			

Supplementary Table S2. Model selection result of BioGeoBears.

BioGeoBEARS result file of
[TAXON]

1	Cchi	c
2	Cjap	cd
3	Clut	a
4	Hbre	d
5	Hbul	a
6	Hfla	d
7	Hkaw	b
8	Hkor	c
9	Hleu	b
10	Hori	d
11	Hsp	b
12	Htub	c
13	Humb	b
14	Ythi	c
15	Yyun	c

[TREE]

Tree=(((2:0.0046566685765182525,1:
0.0046566685765182525):0.0069469
54291384241,3:0.0116036228679024
8):0.06649502815335401,((((12:0.002
487793750976325,8:0.002487793750
976325):0.003299127182947892,(((7:
0.002466209625069972,10:0.0024662
09625069972):
8.625659737625177E-
4,(4:4.2044147764608297E-
4,6:4.2044147764608297E-
4):0.0029083341211864067):8.53210
1629321123E-
4,(9:9.750728981622186E-
4,(11:2.3766859238370797E-
4,13:2.3766859238370797E-
4):7.374043057785107E-
4):0.0032069128636023833):0.00160
49351721596147):0.00571849408926
7015,(15:0.0026491917767920564,14
:0.0026491917767920564):0.0088562
23246399175):0.0328840274939705,
5:0.04438944251716173):0.03370920
8504094756);

[RESULT]
DEC results:
node 16: C 68.521770 CD 31.478230 AC 0.000000 BC 0.000000 D 0.000000 AD 0.000000 BD 0.000000 A 0.000000 B 0.000000 / 0.000000
node 17: AC 87.530740 AD 12.469260 CD 0.000000 C 0.000000 A 0.000000 D 0.000000 BC 0.000000 BD 0.000000 B 0.000000 / 0.000000
node 18: C 100.000000 BC 0.000000 CD 0.000000 AC 0.000000 B 0.000000 D 0.000000 A 0.000000 BD 0.000000 AD 0.000000 / 0.000000
node 19: BD 100.000000 D 0.000000 B 0.000000 CD 0.000000 BC 0.000000 AD 0.000000 C 0.000000 AC 0.000000 A 0.000000 / 0.000000
node 20: D 100.000000 BD 0.000000 CD 0.000000 AD 0.000000 B 0.000000 C 0.000000 BC 0.000000 AC 0.000000 A 0.000000 / 0.000000
node 21: BD 98.920710 D 1.079289 B 0.000000 CD 0.000000 BC 0.000000 AD 0.000000 C 0.000000 AC 0.000000 A 0.000000 / 0.000000
node 22: B 100.000000 BC 0.000000 BD 0.000000 C 0.000000 D 0.000000 CD 0.000000 AC 0.000000 AD 0.000000 / 0.000000 A 0.000000
node 23: B 100.000000 BC 0.000000 BD 0.000000 D 0.000000 C 0.000000 CD 0.000000 AD 0.000000 AC 0.000000 / 0.000000 A 0.000000
node 24: B 56.037540 BD 43.962460 BC 0.000000 D 0.000000 CD 0.000000 C 0.000000 AD 0.000000 AC 0.000000 A 0.000000 / 0.000000
node 25: BC 74.348140 CD 25.651850 C 0.000000 B 0.000000 BD 0.000000 D 0.000000 AC 0.000000 AD 0.000000 A 0.000000 / 0.000000
node 26: C 100.000000 AC 0.000000 BC 0.000000 CD 0.000000 B 0.000000 A 0.000000 D 0.000000 AD 0.000000 BD 0.000000 / 0.000000
node 27: BC 42.422380 C 39.757120 CD 17.820500 AC 0.000000 D 0.000000 AD 0.000000 BD 0.000000 B 0.000000 A 0.000000 / 0.000000
node 28: AC 92.581720 AD 7.418276 C 0.000003 A 0.000002 BC 0.000001 CD 0.000001 D 0.000000 BD 0.000000 B 0.000000 / 0.000000
node 29: AC 61.254490 A 19.873280 C 11.757780 AD 6.181808 CD 0.645750 D 0.286896 BC 0.000000 BD 0.000000 B 0.000000 / 0.000000
DEC+j results:
node 16: C 42.898020 D 2.910203 AD 0.018422 BD 0.002248 BC 0.000478 A 0.000318 B 0.000103 CD 0.000057 AC 0.000000 / 0.000000
node 17: AD 31.625310 A 18.319710 C 13.078400 BC 7.460266 D 2.360624 AC 0.015357 BD 0.008104 B 0.001142 CD 0.001046 / 0.000000
node 18: C 99.986400 BD 0.005987 AD 0.003210 B 0.000088 D 0.000065 A 0.000048 AC 0.000000 CD 0.000000 BC 0.000000 / 0.000000
node 19: D 61.655300 B 36.500500 CD 1.843383 C 0.000232 BD 0.000143 A 0.000104 BC 0.000083 AC 0.000049 AD 0.000000 / 0.000000
node 20: D 99.999270 CD 0.000400 BC 0.000125 B 0.000007 C 0.000002 A 0.000001 BD 0.000000 AC 0.000000 AD 0.000000 / 0.000000
node 21: D 68.337470 B 29.990860 CD 1.669275 C 0.000533 BC 0.000255 BD 0.000248 A 0.000169 AC 0.000039 AD 0.000000 / 0.000000
node 22: B 99.999780 BD 0.000097 CD 0.000081 AC 0.000039 D 0.000000 C 0.000000 A 0.000000 AD 0.000000 BC 0.000000 / 0.000000
node 23: B 99.997250 BD 0.001262 CD 0.001004 AC 0.000407 D 0.000035 C 0.000031 A 0.000010 AD 0.000000 BC 0.000000 / 0.000000
node 24: B 63.762740 D 34.341710 CD 1.884988 BD 0.004999 C 0.002823 A 0.000658 AC 0.000514 BC 0.000141 AD 0.000000 / 0.000000
node 25: C 68.497160 B 10.234450 BD 9.335517 D 6.064372 CD 0.377829 AD 0.008843 A 0.000830 AC 0.000448 BC 0.000215 / 0.000000
node 26: C 99.974240 AD 0.013131 BD 0.006346 A 0.000072 B 0.000053 D 0.000048 AC 0.000000 BC 0.000000 CD 0.000000 / 0.000000
node 27: C 78.740430 BD 9.650088 B 3.231896 D 2.226900 CD 0.148066 AD 0.123335 A 0.003910 AC 0.002657 BC 0.001644 / 0.000000
node 28: AD 33.560990 C 28.601360 A 27.932550 AC 2.400025 BC 1.788159 BD 1.565802 B 1.534356 D 1.453598 CD 0.027207 / 0.000000
node 29: AD 38.407240 A 18.195330 C 18.174580 BC 6.347611 D 2.604700 AC 1.684726 BD 1.340451 B 0.477892 CD 0.295195 / 0.000000
DIVALIKE results:
node 16: C 96.735450 D 0.015204 AD 0.001778 BC 0.001029 BD 0.000985 CD 0.000550 A 0.000005 B 0.000002 AC 0.000000 / 0.000000
node 17: AD 97.852580 BC 1.540048 C 0.271509 BD 0.098601 A 0.096685 AC 0.035191 D 0.003709 CD 0.001545 B 0.000085 / 0.000000
node 18: C 99.998790 BD 0.000623 AD 0.000138 B 0.000002 D 0.000001 A 0.000000 CD 0.000000 AC 0.000000 BC 0.000000 / 0.000000
node 19: CD 97.691970 D 1.806262 B 0.491236 BC 0.003933 BD 0.001329 AC 0.001083 C 0.000006 A 0.000000 AD 0.000000 / 0.000000
node 20: D 99.999790 CD 0.000211 BC 0.000003 B 0.000000 BD 0.000000 AC 0.000000 C 0.000000 A 0.000000 AD 0.000000 / 0.000000
node 21: D 78.616710 CD 20.698850 B 0.680174 BD 0.001811 AC 0.001472 BC 0.000064 C 0.000017 A 0.000001 AD 0.000000 / 0.000000
node 22: B 100.000000 CD 0.000000 BD 0.000000 AC 0.000000 D 0.000000 C 0.000000 A 0.000000 BC 0.000000 AD 0.000000 / 0.000000
node 23: B 99.998760 CD 0.000986 BD 0.000242 AC 0.000019 D 0.000000 C 0.000000 BC 0.000000 A 0.000000 AD 0.000000 / 0.000000
node 24: CD 77.677320 B 21.271120 D 0.830621 BD 0.015440 BC 0.007706 AC 0.000604 C 0.000315 A 0.000007 AD 0.000003 / 0.000000
node 25: BD 60.001500 C 0.268229 B 0.055639 D 0.036793 AD 0.006008 AC 0.003258 CD 0.002727 BC 0.002152 A 0.000013 / 0.000000
node 26: C 99.998630 AD 0.000510 BD 0.000448 A 0.000000 B 0.000000 D 0.000000 AC 0.000000 BC 0.000000 CD 0.000000 / 0.000000
node 27: C 85.091030 BD 8.893135 AD 0.043537 B 0.029363 D 0.019455 AC 0.017301 BC 0.011434 CD 0.009101 A 0.000079 / 0.000000
node 28: AD 78.121010 C 7.428978 AC 4.231588 A 3.883050 BC 3.028380 BD 1.323135 B 0.330482 D 0.237309 CD 0.111496 / 0.000000
node 29: C 30.941630 AD 30.901430 A 30.855130 AC 1.773562 BD 1.762326 BC 1.758766 D 0.094093 CD 0.087206 B 0.070622 / 0.000000
DIVALIKE+j results:
node 16: C 87.224210 D 5.691526 BD 0.000000 AD 0.000000 CD 0.000000 A 0.000000 BC 0.000000 B 0.000000 AC 0.000000 / 0.000000
node 17: AD 74.886670 A 7.699914 C 7.078947 BC 6.429528 D 0.578677 AC 0.000000 BD 0.000000 CD 0.000000 B 0.000000 / 0.000000
node 18: C 100.000000 B 0.000000 D 0.000000 A 0.000000 BD 0.000000 AD 0.000000 CD 0.000000 AC 0.000000 BC 0.000000 / 0.000000
node 19: D 63.245190 B 32.935680 CD 3.819137 C 0.000000 BD 0.000000 BC 0.000000 AC 0.000000 A 0.000000 AD 0.000000 / 0.000000
node 20: D 100.000000 B 0.000000 CD 0.000000 C 0.000000 A 0.000000 BC 0.000000 BD 0.000000 AC 0.000000 AD 0.000000 / 0.000000
node 21: D 70.207960 B 28.497970 CD 1.294069 C 0.000000 A 0.000000 BD 0.000000 AC 0.000000 BC 0.000000 AD 0.000000 / 0.000000
node 22: B 100.000000 D 0.000000 C 0.000000 A 0.000000 CD 0.000000 BD 0.000000 AC 0.000000 BC 0.000000 AD 0.000000 / 0.000000
node 23: B 100.000000 D 0.000000 C 0.000000 CD 0.000000 A 0.000000 BD 0.000000 AC 0.000000 BC 0.000000 AD 0.000000 / 0.000000
node 24: B 60.120140 D 30.477760 CD 9.402103 C 0.000000 BD 0.000000 AC 0.000000 A 0.000000 BC 0.000000 AD 0.000000 / 0.000000
node 25: C 51.382940 B 15.315790 BD 13.382650 D 8.023560 CD 4.871464 AD 0.000000 AC 0.000000 BC 0.000000 A 0.000000 / 0.000000
node 26: C 100.000000 AD 0.000000 A 0.000000 B 0.000000 BD 0.000000 D 0.000000 BC 0.000000 AC 0.000000 CD 0.000000 / 0.000000
node 27: C 70.230000 BD 14.493350 B 3.205854 CD 2.280394 D 1.771457 AD 0.000000 AC 0.000000 A 0.000000 BC 0.000000 / 0.000000
node 28: AD 53.286450 A 15.820980 C 13.136130 AC 7.147424 BC 4.229943 BD 2.239763 B 1.590225 D 0.951869 CD 0.363123 / 0.000000
node 29: AD 30.999170 A 30.069360 C 25.530020 BC 3.720942 AC 2.741770 BD 2.229995 D 0.885846 B 0.478787 CD 0.287322 / 0.000000
BAYAREALIKE results:
node 16: C 96.175250 AD 0.652673 D 0.006118 BD 0.000852 A 0.000582 BC 0.000038 B 0.000001 CD 0.000000 AC 0.000000 / 0.000000
node 17: AD 66.016460 C 25.594990 A 7.458391 BC 0.208855 D 0.156263 BD 0.017638 AC 0.004742 B 0.002752 CD 0.000049 / 0.000000
node 18: C 99.875110 BD 0.080815 AD 0.000283 B 0.000008 D 0.000005 A 0.000000 CD 0.000000 AC 0.000000 BC 0.000000 / 0.000000
node 19: D 41.578350 CD 32.449190 B 25.959800 C 0.006835 BD 0.000151 BC 0.000019 AC 0.000012 A 0.000001 AD 0.000000 / 0.000000
node 20: D 99.973990 CD 0.025916 BC 0.000005 B 0.000001 C 0.000000 A 0.000000 BD 0.000000 AC 0.000000 AD 0.000000 / 0.000000
node 21: D 41.641320 CD 31.991310 B 26.115370 C 0.008561 BD 0.004252 BC 0.000029 AC 0.000005 A 0.000001 AD 0.000000 / 0.000000
node 22: B 99.999960 CD 0.000029 BD 0.000008 AC 0.000001 D 0.000000 C 0.000000 A 0.000000 BC 0.000000 AD 0.000000 / 0.000000
node 23: B 99.908010 CD 0.072666 BD 0.019272 AC 0.000027 D 0.000020 C 0.000005 A 0.000000 BC 0.000000 AD 0.000000 / 0.000000
node 24: B 58.940660 D 31.362330 C 7.873861 CD 0.783510 BD 0.697774 A 0.000625 AC 0.000031 BC 0.000015 AD 0.000003 / 0.000000
node 25: C 87.459060 BD 7.953433 B 0.189080 D 0.105729 AD 0.003084 A 0.000184 CD 0.000170 AC 0.000008 BC 0.000004 / 0.000000
node 26: C 99.982990 BD 0.008051 AD 0.004083 B 0.000000 D 0.000000 A 0.000000 AC 0.000000 BC 0.000000 CD 0.000000 / 0.000000
node 27: C 96.637900 BD 1.607855 AD 0.768361 B 0.052243 D 0.031073 A 0.001238 AC 0.000372 BC 0.000213 CD 0.000022 / 0.000000
node 28: AD 69.705840 C 20.835300 A 7.878170 BD 0.519763 AC 0.213770 BC 0.180034 B 0.106333 D 0.097434 CD 0.001065 / 0.000000
node 29: AD 68.689870 C 19.262980 A 7.853981 BD 1.435333 BC 0.519487 AC 0.464548 D 0.102801 B 0.079468 CD 0.007736 / 0.000000
BAYAREALIKE+j results:
node 16: C 59.949870 D 4.826002 AD 0.079670 BD 0.073135 BC 0.029121 CD 0.022413 A 0.014373 B 0.002762 AC 0.000021 / 0.000000
node 17: A 32.547200 C 28.149920 D 2.449709 AC 1.671479 BC 1.534156 AD 0.962191 BD 0.850266 CD 0.077293 B 0.041863 / 0.000000
node 18: C 99.992540 AD 0.002028 B 0.001480 D 0.001031 BD 0.000920 A 0.000653 BC 0.000001 AC 0.000001 CD 0.000000 / 0.000000
node 19: D 62.462420 B 37.507050 BD 0.007434 AC 0.005934 BC 0.005083 C 0.004242 A 0.001620 CD 0.000624 AD 0.000001 / 0.000000
node 20: D 99.999770 B 0.000121 C 0.000034 CD 0.000026 A 0.000019 BC 0.000013 BD 0.000000 AC 0.000000 AD 0.000000 / 0.000000
node 21: D 68.957550 B 30.998740 BC 0.012100 C 0.011521 BD 0.005095 AC 0.004519 A 0.002796 CD 0.000275 AD 0.000002 / 0.000000
node 22: B 99.999980 D 0.000005 C 0.000005 A 0.000002 BD 0.000001 CD 0.000001 AC 0.000001 AD 0.000000 BC 0.000000 / 0.000000
node 23: B 99.998380 D 0.000520 C 0.000516 AC 0.000171 BD 0.000170 A 0.000126 CD 0.000118 AD 0.000000 BC 0.000000 / 0.000000

node 24: B 65.186900 D 34.568210 AC 0.094178 C 0.046668 BD 0.042074 BC 0.036664 A 0.006878 CD 0.001914 AD 0.000006 / 0.000000
node 25: C 82.464760 B 10.904450 D 6.081172 AC 0.153387 AD 0.152539 BC 0.071062 CD 0.042544 BD 0.037354 A 0.008936 / 0.000000
node 26: C 99.987140 AD 0.004881 BD 0.002030 A 0.000965 B 0.000927 D 0.000782 BC 0.000004 AC 0.000003 CD 0.000001 / 0.000000
node 27: C 90.773190 B 4.393484 D 2.716138 AD 0.774747 AC 0.324491 BD 0.176580 BC 0.168230 CD 0.138284 A 0.081741 / 0.000000
node 28: C 33.600690 A 29.517960 AD 10.335410 BC 5.157240 AC 4.942097 BD 3.311388 B 1.854115 D 1.712606 CD 0.318879 / 0.000000
node 29: C 23.515660 A 20.100540 AD 12.210610 BC 6.716009 AC 6.274493 BD 4.964544 D 3.700937 B 1.455604 CD 0.924983 / 0.000000
[SUPPLEMENT]
#Results of Model Test#

	LnL	numparams	d	e	j	AICc	AICc_wt
DEC	-23.41	2	5	7.20E-07	0	51.82	0.047
DEC+J	-19.67	3	3.09	0.38	0.098	47.52	0.4
DIVALIKE	-25.21	2	5	3.32	0	55.42	0.0077
DIVALIKE+J	-19.43	3	5	1.00E-12	0.065	47.04	0.51
BAYAREALIKE	-43.3	2	5	5	0	91.6	1.10E-10
BAYAREALIKE+J	-22.09	3	3.92	5	0.094	52.37	0.035

Use the highest AICc_wt to select the best model
==

alt	null	LnLalt	LnLnull	DFalt	DFnull	DF	Dstatistic	pval	test	tail	AIC1	AIC2	AICwt1	AICwt2	AICweight_ratio_model1	AICweight_ratio_model2
DEC+J	DEC	-19.67	-23.41	3	2	1	7.48	0.0063	chi-squared	one-tailed	45.34	50.82	0.94	0.061	15.46	0.065
DIVALIKE+J	DIVALIKE	-19.43	-25.21	3	2	1	11.56	0.0007	chi-squared	one-tailed	44.85	54.42	0.99	0.0083	119.2	0.0084
BAYAREALIKE+J	BAYAREALIKE	-22.09	-43.3	3	2	1	42.41	7.40E-11	chi-squared	one-tailed	50.19	90.6	1	1.70E-09	5.96E+08	1.70E-09

The p-value of the LRT (Likelihood Ratio Test) tells you whether or not you can reject
the null hypothesis that without J and +J confer equal likelihoods on the data.
[END]

Supplementary Table S3. The events of ancestral area reconstruction of Heloniadeae

NODE16:
EVENT MATRIX:
Dispersal:1
Vicariance:0
Extinction:0
Event Route:
C->C^C->CD^C->CD|C
PROBABILITY:
0.6852

NODE17:
EVENT MATRIX:
Dispersal:0
Vicariance:1
Extinction:0
Event Route:
AC->A|C
PROBABILITY:
0.5998

NODE18:
EVENT MATRIX:
Dispersal:0
Vicariance:0
Extinction:0
Event Route:
C->C^C->C|C
PROBABILITY:
1.0000

NODE19:
EVENT MATRIX:
Dispersal:0
Vicariance:1
Extinction:0
Event Route:

BD->B|D
PROBABILITY:
1.0000

NODE20:
EVENT MATRIX:
Dispersal:0
Vicariance:0
Extinction:0
Event Route:
D->D^D->D|D
PROBABILITY:
1.0000

NODE21:
EVENT MATRIX:
Dispersal:1
Vicariance:0
Extinction:0
Event Route:
BD->BD^D->D|BD
PROBABILITY:
0.9892

NODE22:
EVENT MATRIX:
Dispersal:0
Vicariance:0
Extinction:0
Event Route:
B->B^B->B|B
PROBABILITY:
1.0000

NODE23:
EVENT MATRIX:
Dispersal:0
Vicariance:0

  Extinction:0
Event Route:
  B->B^B->B|B
PROBABILITY:
  1.0000

NODE24:
EVENT MATRIX:
  Dispersal:1
  Vicariance:0
  Extinction:0
Event Route:
  B->B^B->BD^B->B|BD
PROBABILITY:
  0.5543

NODE25:
EVENT MATRIX:
  Dispersal:0
  Vicariance:1
  Extinction:0
Event Route:
  BC->B|C
PROBABILITY:
  0.4166

NODE26:
EVENT MATRIX:
  Dispersal:0
  Vicariance:0
  Extinction:0
Event Route:
  C->C^C->C|C
PROBABILITY:
  1.0000

NODE27:
EVENT MATRIX:

Dispersal:1
Vicariance:0
Extinction:0
Event Route:
BC->BC^C->C|BC
PROBABILITY:
0.3154

NODE28:
EVENT MATRIX:
Dispersal:1
Vicariance:1
Extinction:0
Event Route:
AC->ABC->A|BC
PROBABILITY:
0.3928

NODE29:
EVENT MATRIX:
Dispersal:2
Vicariance:0
Extinction:0
Event Route:
AC->AC^A^C->AC|AC
PROBABILITY:
0.4964

==================

Dispersal Between Areas:
A->B:0.5
B->D:1
C->B:0.5
C->D:1
Speciation Within Areas:
A:1
B:3
C:5

D:2

Dispersal Table:

	from	to	within
A	0.50	0.00	1
B	1.00	1.00	3
C	1.50	0.00	5
D	0.00	2.00	2

====================

Global Cost:

Global Dispersal: 7

Global Vicariance: 4

Global Extinction: 0

Supplementary Figure S4. Ancient area reconstruction of Heloniadeae, the node number referred the DIVALIKE+J model of supplementary table S3.

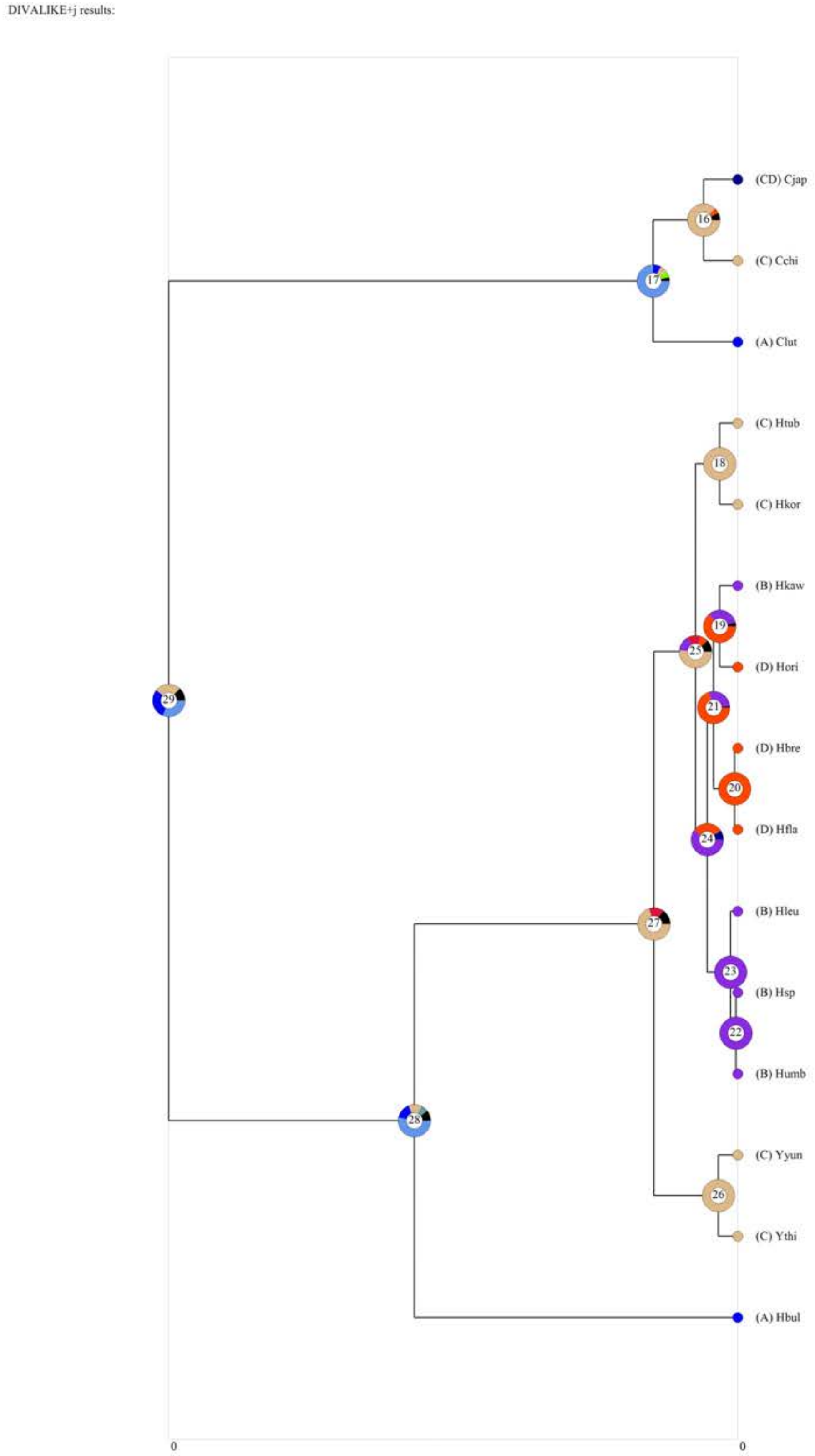

Supplement table S5. Accession numbers of sequences applied in this study.

	atpB_rbcL	matK	trnG	trnK	trnL_F
Helonias bullata	LC086934	AB104838	LC086994	AB109813	LC087054
Heloniopsis kawanoi	LC086935-938	AB040186, AB104835, LC086896-897	LC086995-998	AB109806-807, LC086860-861	LC087055-058
Heloniopsis koreana	LC086939-940	AB109326-327, KM242772	LC086999-7000	AB109320-321	LC087059-060
Heloniopsis leucantha	LC086941-943	AB040187, AB104834, LC086898	LC087001-003	AB109804-805, LC086862	LC087061-063, HG475354
Heloniopsis orientalis var. *orientalis*	LC086944-965	AB040188, LC086899-919	LC087004-025	AB109334, LC086863-883	LC087064-085
Heloniopsis orientalis var. *flavida*	LC098966-971	AB040190, AB104828, LC086920-923	LC087026-031	AB104777, 780, 086884-888	LC087086-091
Heloniopsis orientalis var. *breviscapa*	LC098972-983	AB040194, AB104829-833, LC086924-929	LC086032-043	AB104785, 788-798, 793, 801, 803, LC086889-893	LC087092-103
Heloniopsis tubiflora	LC098984-985	AB109323-324	LC087044-045	AB109317-318	LC087104-105
Heloniopsis umbellata	LC098986-987, MT951070	AB040195, AB104836, KM242776, OK075116, OK075117	LC087046-047, MT990758	AB109809-810, MT990761	LC087106-107, HG475353, MT990764
Heloniopsis sp.	MT951098-099	OK075118	MT990756-757	MT990759-760	MT990762-763
Ypsilandra thibetica	LC098988-989	AB040185, AB104837	LC087048-049	AB109811-812	LC087108-109
Ypsilandra yunnanensis	LC086990	LC086930	LC087050	LC086894	LC087110

Chamaelirium luteum	LC086991	AB040196	LC087051	LC086895	LC087111
Chionographis chinensis	LC086992	AB040197	LC087052	AB109814	LC087112
Chionographis japonica	LC086993	AB040198	LC087053	AB109815	LC087113

Supplement Table S6. Model test result of BioGeoBears

	LnL	numparams	d	e	j	AICc	AICc_wt
DEC	-25.59	2	4.99	4.68	0	56.17	0.0038
DEC+J	-19.33	3	4.01	0.94	0.098	46.84	0.4
DIVALIKE	-26.09	2	5	8.00E-08	0	57.18	0.0023
DIVALIKE+J	-19.03	3	5	1.00E-12	0.073	46.23	0.54
BAYAREALIKE	-48.41	2	5	5	0	101.8	4.60E-13

use the highest AICc_wt to select best model

Supplementary Table S7. Location and database information of Heloniadeae taxa applied for anthesis temperature analysis

n	s_n	decimalLongitude	decimalLatitude	reference	scientificName
1	1	-82.57671	35.202917	https://www.gbif.org/occurrence/2596154211	Helonias bullata
2	2	-82.718627	35.089694	https://www.gbif.org/occurrence/2603386398	Helonias bullata
3	3	-83.00483	35.110178	https://www.gbif.org/occurrence/2603413056	Helonias bullata
4	4	-75.558971	38.828432	https://www.gbif.org/occurrence/2609197697	Helonias bullata
5	5	-74.542094	39.68523	https://www.gbif.org/occurrence/2626190240	Helonias bullata
6	6	-82.748442	35.061556	https://www.gbif.org/occurrence/2626498137	Helonias bullata
7	7	-82.746403	35.064615	https://www.gbif.org/occurrence/2236914862	Helonias bullata
8	8	-82.668195	35.037788	https://www.gbif.org/occurrence/2237508174	Helonias bullata
9	9	-71.068253	42.574474	https://www.gbif.org/occurrence/2451714610	Helonias bullata
10	10	-83.128007	35.125903	https://www.gbif.org/occurrence/2265892402	Helonias bullata
11	11	-83.111173	35.104832	https://www.gbif.org/occurrence/2447919428	Helonias bullata
12	12	-83.199466	35.16533	https://www.gbif.org/occurrence/1838317735	Helonias bullata
13	13	-78.811232	37.878158	https://www.gbif.org/occurrence/1847492290	Helonias bullata
14	14	-82.708884	35.242154	https://www.gbif.org/occurrence/1880496744	Helonias bullata
15	15	-75.482044	38.857072	https://www.gbif.org/occurrence/2350448640	Helonias bullata
16	16	-82.694692	35.201394	https://www.gbif.org/occurrence/2573782936	Helonias bullata
17	17	-82.61466	35.232537	https://www.gbif.org/occurrence/2596439164	Helonias bullata
18	18	-75.374005	39.430358	https://www.gbif.org/occurrence/2251825448	Helonias bullata
19	19	-82.634122	35.295072	https://www.gbif.org/occurrence/1500335168	Helonias bullata
20	20	-82.764905	35.181609	https://www.gbif.org/occurrence/1805376412	Helonias bullata
21	21	-82.363352	34.946855	https://www.gbif.org/occurrence/1805384029	Helonias bullata
22	22	-82.754466	35.282826	https://www.gbif.org/occurrence/2609281212	Helonias bullata
23	23	-76.668084	39.158101	https://www.gbif.org/occurrence/1990443678	Helonias bullata
24	24	-82.781463	35.031812	https://www.gbif.org/occurrence/2573794301	Helonias bullata
25	25	-75.017127	39.447662	https://www.gbif.org/occurrence/1571062316	Helonias bullata
26	26	-76.585643	39.070183	https://www.gbif.org/occurrence/891146344	Helonias bullata
27	27	-74.318304	40.051295	https://www.gbif.org/occurrence/1993746125	Helonias bullata
28	28	-76.493634	39.087696	https://www.gbif.org/occurrence/2251846927	Helonias bullata
29	29	-74.188012	40.124435	https://www.gbif.org/occurrence/1993746047	Helonias bullata
30	30	-82.786816	35.380221	https://www.gbif.org/occurrence/1850955325	Helonias bullata
31	31	-78.821364	37.924166	https://www.gbif.org/occurrence/2460143220	Helonias bullata
32	32	-78.855031	37.826317	https://www.gbif.org/occurrence/2460151376	Helonias bullata
33	33	-83.25	35.46666	https://www.gbif.org/occurrence/1258603728	Helonias bullata
34	34	-83.25	35.46666	https://www.gbif.org/occurrence/1258630424	Helonias bullata
35	35	-74.316407	40.013969	https://www.gbif.org/occurrence/1929153150	Helonias bullata
36	36	-74.996287	39.821752	https://www.gbif.org/occurrence/1929907452	Helonias bullata
37	37	-75.258146	39.490513	https://www.gbif.org/occurrence/1928296907	Helonias bullata
38	38	-74.217644	40.097893	https://www.gbif.org/occurrence/1929027524	Helonias bullata
39	39	-74.625715	40.822877	https://www.gbif.org/occurrence/1928525663	Helonias bullata
40	40	-74.734052	40.87121	https://www.gbif.org/occurrence/1929220194	Helonias bullata
41	41	-74.75166	40.86305	https://www.gbif.org/occurrence/415933286	Helonias bullata
42	42	-74.982944	39.811503	https://www.gbif.org/occurrence/1928770497	Helonias bullata
43	43	-74.734052	40.87121	https://www.gbif.org/occurrence/1929045563	Helonias bullata
44	44	-74.734052	40.87121	https://www.gbif.org/occurrence/1929062247	Helonias bullata
45	45	-73.877023	40.862289	https://www.gbif.org/occurrence/1929407669	Helonias bullata
46	46	-74.89	39.7683	https://www.gbif.org/occurrence/1930856616	Helonias bullata
47	47	-74.75166	40.86305	https://www.gbif.org/occurrence/606653044	Helonias bullata
48	48	-74.75166	40.86305	https://www.gbif.org/occurrence/606650968	Helonias bullata
49	49	-74.734052	40.87121	https://www.gbif.org/occurrence/1930718120	Helonias bullata
50	50	-75.0249	39.5465	https://www.gbif.org/occurrence/1056514445	Helonias bullata
51	51	-75.74965	39.68372	https://www.gbif.org/occurrence/606650967	Helonias bullata
52	52	-75.13	39.86972	https://www.gbif.org/occurrence/606650964	Helonias bullata
53	53	-75.13	39.86972	https://www.gbif.org/occurrence/606650966	Helonias bullata
54	54	-75	40	https://www.gbif.org/occurrence/1228275077	Helonias bullata
55	55	-74.357649	40.523438	https://www.gbif.org/occurrence/1929933754	Helonias bullata
56	56	-75	40	https://www.gbif.org/occurrence/1228292400	Helonias bullata
57	57	-74.217644	40.097893	https://www.gbif.org/occurrence/1928285002	Helonias bullata
58	58	-74.290702	40.477884	https://www.gbif.org/occurrence/1928400642	Helonias bullata
59	2	128.143182	37.459917	https://www.gbif.org/occurrence/2596203019	Heloniopsis koreana
60	3	128.052006	37.340132	https://www.gbif.org/occurrence/2597707139	Heloniopsis koreana
61	4	128.143182	37.459917	https://www.gbif.org/occurrence/2236840853	Heloniopsis koreana
62	5	128.143182	37.459917	https://www.gbif.org/occurrence/2236917598	Heloniopsis koreana
63	6	128.397714	37.363407	https://www.gbif.org/occurrence/2609424257	Heloniopsis koreana
64	7	128.142471	37.459326	https://www.gbif.org/occurrence/1802636710	Heloniopsis koreana
65	8	128.91524	37.095739	https://www.gbif.org/occurrence/1668792499	Heloniopsis koreana
66	9	128.410611	38.102111	https://www.gbif.org/occurrence/2609423970	Heloniopsis koreana
67	10	127.959111	37.661222	https://www.gbif.org/occurrence/2609423374	Heloniopsis koreana
68	11	127.402888	37.013222	https://www.gbif.org/occurrence/2609424729	Heloniopsis koreana
69	12	128.137	38.208333	https://www.gbif.org/occurrence/2609422875	Heloniopsis koreana
70	13	128.079055	38.266638	https://www.gbif.org/occurrence/2609426183	Heloniopsis koreana
71	15	127.08109	37.69828	https://www.gbif.org/occurrence/2609426289	Heloniopsis koreana
72	16	128.895833	37.086111	https://www.gbif.org/occurrence/2609423209	Heloniopsis koreana
73	17	126.935444	37.2571	https://www.gbif.org/occurrence/2609421264	Heloniopsis koreana
74	18	127.444087	38.07485	https://www.gbif.org/occurrence/2609423378	Heloniopsis koreana

75	19	127.036111	37.341667	https://www.gbif.org/occurrence/2609426167	Heloniopsis koreana
76	20	126.935444	37.2571	https://www.gbif.org/occurrence/2609422527	Heloniopsis koreana
77	22	127.186111	37.475	https://www.gbif.org/occurrence/2609424051	Heloniopsis koreana
78	23	127.036111	37.341667	https://www.gbif.org/occurrence/2609422717	Heloniopsis koreana
79	24	127.163889	37.750556	https://www.gbif.org/occurrence/2609424119	Heloniopsis koreana
80	25	127.163889	37.750556	https://www.gbif.org/occurrence/2609424391	Heloniopsis koreana
81	29	126.935444	37.2571	https://www.gbif.org/occurrence/2609423915	Heloniopsis koreana
82	30	126.935444	37.2571	https://www.gbif.org/occurrence/2609424296	Heloniopsis koreana
83	31	128.425603	38.048804	https://www.gbif.org/occurrence/2609423299	Heloniopsis koreana
84	32	126.964057	37.445215	https://www.gbif.org/occurrence/2609422554	Heloniopsis koreana
85	33	127.147239	37.74003	https://www.gbif.org/occurrence/2609426992	Heloniopsis koreana
86	34	126.964057	37.445215	https://www.gbif.org/occurrence/2609423481	Heloniopsis koreana
87	37	126.964057	37.445215	https://www.gbif.org/occurrence/2609423109	Heloniopsis koreana
88	38	126.964057	37.445215	https://www.gbif.org/occurrence/2609423367	Heloniopsis koreana
89	39	126.964057	37.445215	https://www.gbif.org/occurrence/2609423077	Heloniopsis koreana
90	45	129.794444	42.76339	https://www.gbif.org/occurrence/2443058329	Heloniopsis koreana
91	1	139.101094	35.439622	https://www.gbif.org/occurrence/1829942864	Heloniopsis orientalis var. breviscapa
92	2	139.040594	35.232447	https://www.gbif.org/occurrence/1829929140	Heloniopsis orientalis var. breviscapa
93	3	139.22807	35.424094	https://www.gbif.org/occurrence/1829917534	Heloniopsis orientalis var. breviscapa
94	4	139.053091	35.224114	https://www.gbif.org/occurrence/1829919426	Heloniopsis orientalis var. breviscapa
95	5	139.053091	35.224114	https://www.gbif.org/occurrence/2244425743	Heloniopsis orientalis var. breviscapa
96	6	135.695	34.538056	https://www.gbif.org/occurrence/1830519592	Heloniopsis orientalis var. breviscapa
97	7	130.72838	33.330513	https://www.gbif.org/occurrence/1829852569	Heloniopsis orientalis var. breviscapa
98	8	131.041385	32.857539	https://www.gbif.org/occurrence/388830938	Heloniopsis orientalis var. breviscapa
99	9	139.040599	35.124125	https://www.gbif.org/occurrence/1829868229	Heloniopsis orientalis var. breviscapa
100	10	131.199444	32.938889	https://www.gbif.org/occurrence/1829908391	Heloniopsis orientalis var. breviscapa
101	11	139.101094	35.439622	https://www.gbif.org/occurrence/1829942864	Heloniopsis orientalis var. breviscapa
102	12	139.040594	35.232447	https://www.gbif.org/occurrence/1829929140	Heloniopsis orientalis var. breviscapa
103	13	130.957778	33.114444	https://www.gbif.org/occurrence/2243101756	Heloniopsis orientalis var. breviscapa
104	14	139.22807	35.424094	https://www.gbif.org/occurrence/1829917534	Heloniopsis orientalis var. breviscapa
105	15	139.053091	35.224114	https://www.gbif.org/occurrence/1829919426	Heloniopsis orientalis var. breviscapa
106	16	139.053091	35.224114	https://www.gbif.org/occurrence/2244425743	Heloniopsis orientalis var. breviscapa
107	17	130.82	31.48000003	https://www.gbif.org/occurrence/1931746128	Heloniopsis orientalis var. breviscapa
108	18	135.695	34.538056	https://www.gbif.org/occurrence/1830519592	Heloniopsis orientalis var. breviscapa
109	19	130.72838	33.330513	https://www.gbif.org/occurrence/1829852569	Heloniopsis orientalis var. breviscapa
110	20	131.45	32.066667	https://www.gbif.org/occurrence/1933897215	Heloniopsis orientalis var. breviscapa
111	21	131.041385	32.857539	https://www.gbif.org/occurrence/388830938	Heloniopsis orientalis var. breviscapa
112	22	132.19	34.566944	https://www.gbif.org/occurrence/1830525004	Heloniopsis orientalis var. breviscapa
113	23	134.083333	33.466667	https://www.gbif.org/occurrence/1830671522	Heloniopsis orientalis var. breviscapa
114	24	133.583333	33.633333	https://www.gbif.org/occurrence/1830671525	Heloniopsis orientalis var. breviscapa
115	25	134.216667	33.566667	https://www.gbif.org/occurrence/1830671582	Heloniopsis orientalis var. breviscapa
116	26	133.583333	33.633333	https://www.gbif.org/occurrence/1830672902	Heloniopsis orientalis var. breviscapa
117	27	139.040599	35.124125	https://www.gbif.org/occurrence/1829868229	Heloniopsis orientalis var. breviscapa
118	28	134.233333	33.533333	https://www.gbif.org/occurrence/1830671545	Heloniopsis orientalis var. breviscapa
119	29	134.487713	34.01951	https://www.gbif.org/occurrence/1830624396	Heloniopsis orientalis var. breviscapa
120	30	134.221381	33.585399	https://www.gbif.org/occurrence/1830631861	Heloniopsis orientalis var. breviscapa
121	31	133.55	33.766667	https://www.gbif.org/occurrence/2244521481	Heloniopsis orientalis var. breviscapa
122	32	133.55	33.766667	https://www.gbif.org/occurrence/2244522934	Heloniopsis orientalis var. breviscapa
123	33	133.566667	33.75	https://www.gbif.org/occurrence/2244523047	Heloniopsis orientalis var. breviscapa
124	34	134.327095	33.818648	https://www.gbif.org/occurrence/1830631502	Heloniopsis orientalis var. breviscapa
125	35	134.21902	33.555597	https://www.gbif.org/occurrence/1830643510	Heloniopsis orientalis var. breviscapa
126	36	130.9769	32.6343	https://www.gbif.org/occurrence/1933931030	Heloniopsis orientalis var. breviscapa
127	37	131.199444	32.938889	https://www.gbif.org/occurrence/1829908391	Heloniopsis orientalis var. breviscapa
128	38	130.9592	32.6428	https://www.gbif.org/occurrence/1933931022	Heloniopsis orientalis var. breviscapa
129	39	131.0585	33.1517	https://www.gbif.org/occurrence/1933931072	Heloniopsis orientalis var. breviscapa
130	40	131.3939	32.3295	https://www.gbif.org/occurrence/1933931089	Heloniopsis orientalis var. breviscapa
131	41	131.4403	33.1456	https://www.gbif.org/occurrence/1933931102	Heloniopsis orientalis var. breviscapa
132	42	133.7536	33.961	https://www.gbif.org/occurrence/1933931039	Heloniopsis orientalis var. breviscapa
133	43	134.2258	34.0401	https://www.gbif.org/occurrence/1933931046	Heloniopsis orientalis var. breviscapa
134	44	133.3776	33.9273	https://www.gbif.org/occurrence/1933931101	Heloniopsis orientalis var. breviscapa
135	45	130.3496	33.483	https://www.gbif.org/occurrence/1933931015	Heloniopsis orientalis var. breviscapa
136	46	131.19661	32.89853	https://www.gbif.org/occurrence/1933826878	Heloniopsis orientalis var. breviscapa
137	47	131.19661	32.89853	https://www.gbif.org/occurrence/1933826879	Heloniopsis orientalis var. breviscapa
138	48	130.72908	32.35936	https://www.gbif.org/occurrence/1933826872	Heloniopsis orientalis var. breviscapa
139	49	130.72908	32.35936	https://www.gbif.org/occurrence/1933826897	Heloniopsis orientalis var. breviscapa
140	50	130.72908	32.35936	https://www.gbif.org/occurrence/1933826954	Heloniopsis orientalis var. breviscapa
141	51	140.15555	35.159	https://www.gbif.org/occurrence/1933873121	Heloniopsis orientalis var. breviscapa
142	52	140.1523	35.1623	https://www.gbif.org/occurrence/1933930901	Heloniopsis orientalis var. breviscapa
143	53	134.325233	33.887	https://www.gbif.org/occurrence/1830621099	Heloniopsis orientalis var. breviscapa
144	54	133.586693	33.641965	https://www.gbif.org/occurrence/1830641635	Heloniopsis orientalis var. breviscapa
145	55	130.90202	33.48381	https://www.gbif.org/occurrence/1933910174	Heloniopsis orientalis var. breviscapa
146	56	130.90202	33.48381	https://www.gbif.org/occurrence/1933910188	Heloniopsis orientalis var. breviscapa
147	57	140.1523	35.1623	https://www.gbif.org/occurrence/1933930968	Heloniopsis orientalis var. breviscapa
148	58	140.15555	35.159	https://www.gbif.org/occurrence/1933873042	Heloniopsis orientalis var. breviscapa
149	59	139.226	35.5225	https://www.gbif.org/occurrence/1933976971	Heloniopsis orientalis var. breviscapa
150	60	139.226	35.5225	https://www.gbif.org/occurrence/1933977023	Heloniopsis orientalis var. breviscapa
151	61	140.15555	35.159	https://www.gbif.org/occurrence/1933873182	Heloniopsis orientalis var. breviscapa

152	62	140.15555	35.159	https://www.gbif.org/occurrence/1933873204	Heloniopsis orientalis var. breviscapa
153	63	139.2821	35.5282	https://www.gbif.org/occurrence/1933930934	Heloniopsis orientalis var. breviscapa
154	64	139.1658	35.47108	https://www.gbif.org/occurrence/1830215552	Heloniopsis orientalis var. breviscapa
155	65	140.15555	35.159	https://www.gbif.org/occurrence/1933872804	Heloniopsis orientalis var. breviscapa
156	66	130.9992	32.9175	https://www.gbif.org/occurrence/1933931061	Heloniopsis orientalis var. breviscapa
157	67	139.4482	35.6525	https://www.gbif.org/occurrence/1933992290	Heloniopsis orientalis var. breviscapa
158	68	131.2463	31.6374	https://www.gbif.org/occurrence/1933931070	Heloniopsis orientalis var. breviscapa
159	69	131.2463	31.6374	https://www.gbif.org/occurrence/1933931082	Heloniopsis orientalis var. breviscapa
160	70	131.3798	31.689	https://www.gbif.org/occurrence/1933931055	Heloniopsis orientalis var. breviscapa
161	71	131.3798	31.689	https://www.gbif.org/occurrence/1933931071	Heloniopsis orientalis var. breviscapa
162	72	131.1847	31.678	https://www.gbif.org/occurrence/1933977059	Heloniopsis orientalis var. breviscapa
163	73	130.0907	32.9641	https://www.gbif.org/occurrence/1934164694	Heloniopsis orientalis var. breviscapa
164	74	139.4516	35.651	https://www.gbif.org/occurrence/1933992286	Heloniopsis orientalis var. breviscapa
165	75	140.1523	35.1623	https://www.gbif.org/occurrence/1933930914	Heloniopsis orientalis var. breviscapa
166	76	140.1523	35.1623	https://www.gbif.org/occurrence/1933930860	Heloniopsis orientalis var. breviscapa
167	77	140.1523	35.1623	https://www.gbif.org/occurrence/1933930923	Heloniopsis orientalis var. breviscapa
168	78	140.1523	35.1623	https://www.gbif.org/occurrence/1933930947	Heloniopsis orientalis var. breviscapa
169	79	140.1523	35.1623	https://www.gbif.org/occurrence/1933926168	Heloniopsis orientalis var. breviscapa
170	80	140.1523	35.1623	https://www.gbif.org/occurrence/1933930906	Heloniopsis orientalis var. breviscapa
171	81	140.1523	35.1623	https://www.gbif.org/occurrence/1933926178	Heloniopsis orientalis var. breviscapa
172	82	134.4486	33.9785	https://www.gbif.org/occurrence/1933930907	Heloniopsis orientalis var. breviscapa
173	83	139.046	35.212	https://www.gbif.org/occurrence/1934003239	Heloniopsis orientalis var. breviscapa
174	84	134.19867	34.01394	https://www.gbif.org/occurrence/1933941656	Heloniopsis orientalis var. breviscapa
175	85	140.1523	35.1623	https://www.gbif.org/occurrence/1933905009	Heloniopsis orientalis var. breviscapa
176	1	139.028094	35.299103	https://www.gbif.org/occurrence/1829943363	Heloniopsis orientalis var. flavida
177	2	140.015506	35.165811	https://www.gbif.org/occurrence/1829937518	Heloniopsis orientalis var. flavida
178	3	139.028094	35.299103	https://www.gbif.org/occurrence/1829942052	Heloniopsis orientalis var. flavida
179	4	135.25	34.75	https://www.gbif.org/occurrence/2244511992	Heloniopsis orientalis var. flavida
180	5	135.20625	34.720833	https://www.gbif.org/occurrence/1830537301	Heloniopsis orientalis var. flavida
181	6	133.75157	33.759232	https://www.gbif.org/occurrence/1829866993	Heloniopsis orientalis var. flavida
182	7	135.91115	34.56447	https://www.gbif.org/occurrence/204379789	Heloniopsis orientalis var. flavida
183	8	135.9241	34.56277	https://www.gbif.org/occurrence/204379790	Heloniopsis orientalis var. flavida
184	9	135.86116	34.46801	https://www.gbif.org/occurrence/204379782	Heloniopsis orientalis var. flavida
185	10	135.85034	34.44655	https://www.gbif.org/occurrence/204379783	Heloniopsis orientalis var. flavida
186	11	135.86116	34.46801	https://www.gbif.org/occurrence/204379784	Heloniopsis orientalis var. flavida
187	12	134.183333	33.566667	https://www.gbif.org/occurrence/1830678444	Heloniopsis orientalis var. flavida
188	13	133.883333	33.633333	https://www.gbif.org/occurrence/1830680903	Heloniopsis orientalis var. flavida
189	14	132.333333	34.416667	https://www.gbif.org/occurrence/1830520013	Heloniopsis orientalis var. flavida
190	15	133.3	33.716667	https://www.gbif.org/occurrence/1830678265	Heloniopsis orientalis var. flavida
191	16	133.833333	33.616667	https://www.gbif.org/occurrence/1830680369	Heloniopsis orientalis var. flavida
192	17	134.05	33.566667	https://www.gbif.org/occurrence/1830680377	Heloniopsis orientalis var. flavida
193	18	133.6	33.733333	https://www.gbif.org/occurrence/1830680918	Heloniopsis orientalis var. flavida
194	19	133.3	33.716667	https://www.gbif.org/occurrence/1934052265	Heloniopsis orientalis var. flavida
195	20	139.178073	35.457421	https://www.gbif.org/occurrence/388830889	Heloniopsis orientalis var. flavida
196	21	133.9	33.733333	https://www.gbif.org/occurrence/1830686145	Heloniopsis orientalis var. flavida
197	22	133.766667	33.783333	https://www.gbif.org/occurrence/1830686186	Heloniopsis orientalis var. flavida
198	23	133.566667	33.733333	https://www.gbif.org/occurrence/1830686307	Heloniopsis orientalis var. flavida
199	24	135.73318	34.4429	https://www.gbif.org/occurrence/204379779	Heloniopsis orientalis var. flavida
200	25	135.233333	34.75	https://www.gbif.org/occurrence/1933838527	Heloniopsis orientalis var. flavida
201	26	139.10308	35.440754	https://www.gbif.org/occurrence/388830851	Heloniopsis orientalis var. flavida
202	27	139.040593	35.224114	https://www.gbif.org/occurrence/388830853	Heloniopsis orientalis var. flavida
203	28	135.95116	34.49162	https://www.gbif.org/occurrence/204379791	Heloniopsis orientalis var. flavida
204	29	135.87511	34.4044	https://www.gbif.org/occurrence/204379788	Heloniopsis orientalis var. flavida
205	30	133.910889	35.091556	https://www.gbif.org/occurrence/395767211	Heloniopsis orientalis var. flavida
206	31	139.078089	35.215783	https://www.gbif.org/occurrence/388830845	Heloniopsis orientalis var. flavida
207	32	139.10308	35.440754	https://www.gbif.org/occurrence/388830813	Heloniopsis orientalis var. flavida
208	33	139.10308	35.440754	https://www.gbif.org/occurrence/388830814	Heloniopsis orientalis var. flavida
209	34	138.9906	35.21578	https://www.gbif.org/occurrence/388830829	Heloniopsis orientalis var. flavida
210	35	135.84082	34.54226	https://www.gbif.org/occurrence/204379785	Heloniopsis orientalis var. flavida
211	36	139.053086	35.490748	https://www.gbif.org/occurrence/388830816	Heloniopsis orientalis var. flavida
212	37	134.83299	35.0667	https://www.gbif.org/occurrence/729297334	Heloniopsis orientalis var. flavida
213	38	135.89224	34.42646	https://www.gbif.org/occurrence/204379780	Heloniopsis orientalis var. flavida
214	39	135.70021	34.35019	https://www.gbif.org/occurrence/204379781	Heloniopsis orientalis var. flavida
215	40	135.25	34.766701	https://www.gbif.org/occurrence/729278954	Heloniopsis orientalis var. flavida
216	42	135.33299	34.849998	https://www.gbif.org/occurrence/729316242	Heloniopsis orientalis var. flavida
217	43	135.25	34.733299	https://www.gbif.org/occurrence/729276281	Heloniopsis orientalis var. flavida
218	44	135.683333	34.533333	https://www.gbif.org/occurrence/1933930926	Heloniopsis orientalis var. flavida
219	45	135.70073	34.51492	https://www.gbif.org/occurrence/204379775	Heloniopsis orientalis var. flavida
220	46	135.92606	34.56942	https://www.gbif.org/occurrence/204379776	Heloniopsis orientalis var. flavida
221	47	139.103079	35.524079	https://www.gbif.org/occurrence/295907970	Heloniopsis orientalis var. flavida
222	48	135.25	34.733299	https://www.gbif.org/occurrence/729276235	Heloniopsis orientalis var. flavida
223	49	135.2	34.716702	https://www.gbif.org/occurrence/729275798	Heloniopsis orientalis var. flavida
224	50	135.233	34.783298	https://www.gbif.org/occurrence/729277797	Heloniopsis orientalis var. flavida
225	51	135.217	34.75	https://www.gbif.org/occurrence/729278996	Heloniopsis orientalis var. flavida
226	52	135.317	34.766701	https://www.gbif.org/occurrence/729294502	Heloniopsis orientalis var. flavida
227	53	135.317	34.8167	https://www.gbif.org/occurrence/729295549	Heloniopsis orientalis var. flavida
228	54	135.267	34.783298	https://www.gbif.org/occurrence/729276140	Heloniopsis orientalis var. flavida

229	55	135.217	34.75	https://www.gbif.org/occurrence/729279048	Heloniopsis orientalis var. flavida
230	56	135.283	34.849998	https://www.gbif.org/occurrence/729277017	Heloniopsis orientalis var. flavida
231	57	135.71767	34.49286	https://www.gbif.org/occurrence/204379777	Heloniopsis orientalis var. flavida
232	58	135.79846	34.40693	https://www.gbif.org/occurrence/204379792	Heloniopsis orientalis var. flavida
233	59	135.25	34.766701	https://www.gbif.org/occurrence/729277224	Heloniopsis orientalis var. flavida
234	60	135.25	34.766701	https://www.gbif.org/occurrence/729278789	Heloniopsis orientalis var. flavida
235	61	135.25	34.75	https://www.gbif.org/occurrence/729278814	Heloniopsis orientalis var. flavida
236	62	135.233	34.766701	https://www.gbif.org/occurrence/729279030	Heloniopsis orientalis var. flavida
237	63	139.190572	35.465754	https://www.gbif.org/occurrence/295907749	Heloniopsis orientalis var. flavida
238	64	139.10308	35.440754	https://www.gbif.org/occurrence/295907746	Heloniopsis orientalis var. flavida
239	65	140.159244	35.328289	https://www.gbif.org/occurrence/2576017009	Heloniopsis orientalis var. flavida
240	66	140.171742	35.328289	https://www.gbif.org/occurrence/624040623	Heloniopsis orientalis var. flavida
241	67	135.217	34.766701	https://www.gbif.org/occurrence/729277447	Heloniopsis orientalis var. flavida
242	68	135.7424	34.46184	https://www.gbif.org/occurrence/204379778	Heloniopsis orientalis var. flavida
243	69	135.33299	34.849998	https://www.gbif.org/occurrence/729316689	Heloniopsis orientalis var. flavida
244	70	139.065591	35.24078	https://www.gbif.org/occurrence/295907657	Heloniopsis orientalis var. flavida
245	71	139.290564	35.449092	https://www.gbif.org/occurrence/295907116	Heloniopsis orientalis var. flavida
246	72	139.078083	35.499081	https://www.gbif.org/occurrence/295907117	Heloniopsis orientalis var. flavida
247	73	140.146744	35.161647	https://www.gbif.org/occurrence/2576018991	Heloniopsis orientalis var. flavida
248	74	135.3	34.783298	https://www.gbif.org/occurrence/729273109	Heloniopsis orientalis var. flavida
249	75	135.3	34.783298	https://www.gbif.org/occurrence/729273110	Heloniopsis orientalis var. flavida
250	76	134.2483	33.783	https://www.gbif.org/occurrence/1933931088	Heloniopsis orientalis var. flavida
251	77	134.83299	35.0667	https://www.gbif.org/occurrence/729294121	Heloniopsis orientalis var. flavida
252	78	139.140576	35.499082	https://www.gbif.org/occurrence/295907112	Heloniopsis orientalis var. flavida
253	79	139.240569	35.449091	https://www.gbif.org/occurrence/295907115	Heloniopsis orientalis var. flavida
254	80	135.6095	34.1812	https://www.gbif.org/occurrence/1933930878	Heloniopsis orientalis var. flavida
255	81	139.253066	35.540747	https://www.gbif.org/occurrence/295907114	Heloniopsis orientalis var. flavida
256	82	133.4324	33.5462	https://www.gbif.org/occurrence/43212330	Heloniopsis orientalis var. flavida
257	83	139.140576	35.499082	https://www.gbif.org/occurrence/295907113	Heloniopsis orientalis var. flavida
258	84	133.75625	33.754167	https://www.gbif.org/occurrence/1830663634	Heloniopsis orientalis var. flavida
259	85	133.53574	33.55642	https://www.gbif.org/occurrence/43212328	Heloniopsis orientalis var. flavida
260	86	133.79375	33.7625	https://www.gbif.org/occurrence/1830663176	Heloniopsis orientalis var. flavida
261	87	134.28049	33.51547	https://www.gbif.org/occurrence/43212258	Heloniopsis orientalis var. flavida
262	88	134.208734	34.008925	https://www.gbif.org/occurrence/1829859235	Heloniopsis orientalis var. flavida
263	89	134.208734	34.008925	https://www.gbif.org/occurrence/1829859954	Heloniopsis orientalis var. flavida
264	90	134.208734	34.008925	https://www.gbif.org/occurrence/1829859958	Heloniopsis orientalis var. flavida
265	91	132.05695	34.464	https://www.gbif.org/occurrence/1933826737	Heloniopsis orientalis var. flavida
266	92	135.5912	34.2156	https://www.gbif.org/occurrence/1933954471	Heloniopsis orientalis var. flavida
267	93	131.637	34.4857	https://www.gbif.org/occurrence/1933930897	Heloniopsis orientalis var. flavida
268	94	131.9415	34.1681	https://www.gbif.org/occurrence/1933930902	Heloniopsis orientalis var. flavida
269	95	132.0252	34.3739	https://www.gbif.org/occurrence/1933930912	Heloniopsis orientalis var. flavida
270	96	131.7405	34.3684	https://www.gbif.org/occurrence/1933930925	Heloniopsis orientalis var. flavida
271	97	131.7405	34.3684	https://www.gbif.org/occurrence/1934045931	Heloniopsis orientalis var. flavida
272	98	133.53574	33.55642	https://www.gbif.org/occurrence/43211857	Heloniopsis orientalis var. flavida
273	99	133.37758	33.87172	https://www.gbif.org/occurrence/1933931104	Heloniopsis orientalis var. flavida
274	100	131.9415	34.1681	https://www.gbif.org/occurrence/1933930881	Heloniopsis orientalis var. flavida
275	101	134.65193	33.95582	https://www.gbif.org/occurrence/43211855	Heloniopsis orientalis var. flavida
276	102	133.65704	33.59174	https://www.gbif.org/occurrence/43212305	Heloniopsis orientalis var. flavida
277	103	132.0283	34.0614	https://www.gbif.org/occurrence/1933930920	Heloniopsis orientalis var. flavida
278	104	132.0252	34.3739	https://www.gbif.org/occurrence/1933930949	Heloniopsis orientalis var. flavida
279	105	133.53574	33.55642	https://www.gbif.org/occurrence/43212310	Heloniopsis orientalis var. flavida
280	106	133.64203	33.58078	https://www.gbif.org/occurrence/43212312	Heloniopsis orientalis var. flavida
281	107	134.65193	33.95582	https://www.gbif.org/occurrence/43212334	Heloniopsis orientalis var. flavida
282	108	134.44717	34.0782	https://www.gbif.org/occurrence/43212331	Heloniopsis orientalis var. flavida
283	109	136.1099	34.9441	https://www.gbif.org/occurrence/1933977040	Heloniopsis orientalis var. flavida
284	110	134.53708	33.73306	https://www.gbif.org/occurrence/43212343	Heloniopsis orientalis var. flavida
285	111	133.53574	33.55642	https://www.gbif.org/occurrence/43212306	Heloniopsis orientalis var. flavida
286	112	136.5127	34.2889	https://www.gbif.org/occurrence/1933930856	Heloniopsis orientalis var. flavida
287	113	136.5127	34.2889	https://www.gbif.org/occurrence/1933930916	Heloniopsis orientalis var. flavida
288	114	133.53574	33.55642	https://www.gbif.org/occurrence/43212307	Heloniopsis orientalis var. flavida
289	115	136.4455	35.0158	https://www.gbif.org/occurrence/1933976987	Heloniopsis orientalis var. flavida
290	116	136.4455	35.0158	https://www.gbif.org/occurrence/1933977029	Heloniopsis orientalis var. flavida
291	117	136.4292	35.019	https://www.gbif.org/occurrence/1933992282	Heloniopsis orientalis var. flavida
292	118	136.4292	35.019	https://www.gbif.org/occurrence/1933992381	Heloniopsis orientalis var. flavida
293	119	135.79846	34.40693	https://www.gbif.org/occurrence/204379787	Heloniopsis orientalis var. flavida
294	120	133.53574	33.55642	https://www.gbif.org/occurrence/43212309	Heloniopsis orientalis var. flavida
295	121	136.0908	34.5603	https://www.gbif.org/occurrence/1933966138	Heloniopsis orientalis var. flavida
296	122	136.7011	34.4826	https://www.gbif.org/occurrence/1933966122	Heloniopsis orientalis var. flavida
297	123	134.36575	34.06585	https://www.gbif.org/occurrence/43212320	Heloniopsis orientalis var. flavida
298	124	139.22807	35.440758	https://www.gbif.org/occurrence/295907834	Heloniopsis orientalis var. flavida
299	125	133.53574	33.55642	https://www.gbif.org/occurrence/43212308	Heloniopsis orientalis var. flavida
300	126	135.85925	34.51769	https://www.gbif.org/occurrence/204379786	Heloniopsis orientalis var. flavida
301	127	133.81232	34.03289	https://www.gbif.org/occurrence/43212316	Heloniopsis orientalis var. flavida
302	128	134.36575	34.06585	https://www.gbif.org/occurrence/43212315	Heloniopsis orientalis var. flavida
303	129	134.44717	34.0782	https://www.gbif.org/occurrence/43212318	Heloniopsis orientalis var. flavida
304	130	134.44717	34.0782	https://www.gbif.org/occurrence/43212319	Heloniopsis orientalis var. flavida
305	131	134.36575	34.06585	https://www.gbif.org/occurrence/43212314	Heloniopsis orientalis var. flavida

306	132	134.5198	33.8741	https://www.gbif.org/occurrence/1934144131	Heloniopsis orientalis var. flavida
307	133	134.65193	33.95582	https://www.gbif.org/occurrence/43212325	Heloniopsis orientalis var. flavida
308	134	134.65193	33.95582	https://www.gbif.org/occurrence/43212326	Heloniopsis orientalis var. flavida
309	135	133.81232	34.03289	https://www.gbif.org/occurrence/43212336	Heloniopsis orientalis var. flavida
310	136	133.81232	34.03289	https://www.gbif.org/occurrence/43212338	Heloniopsis orientalis var. flavida
311	137	133.81232	34.03289	https://www.gbif.org/occurrence/43212340	Heloniopsis orientalis var. flavida
312	138	134.57877	34.16953	https://www.gbif.org/occurrence/43212322	Heloniopsis orientalis var. flavida
313	139	135.8452	35.0059	https://www.gbif.org/occurrence/1933930945	Heloniopsis orientalis var. flavida
314	140	133.71242	33.56778	https://www.gbif.org/occurrence/43212302	Heloniopsis orientalis var. flavida
315	141	133.71242	33.56778	https://www.gbif.org/occurrence/43212303	Heloniopsis orientalis var. flavida
316	142	135.5912	34.2156	https://www.gbif.org/occurrence/1934003219	Heloniopsis orientalis var. flavida
317	143	135.5912	34.2156	https://www.gbif.org/occurrence/1934003243	Heloniopsis orientalis var. flavida
318	144	133.6545	33.9506	https://www.gbif.org/occurrence/1934096640	Heloniopsis orientalis var. flavida
319	145	135.941	34.2524	https://www.gbif.org/occurrence/1933992281	Heloniopsis orientalis var. flavida
320	1	136.37796	34.99507	https://www.gbif.org/occurrence/2576089507	Heloniopsis orientalis var. orientalis
321	2	135.03125	34.820833	https://www.gbif.org/occurrence/1830530813	Heloniopsis orientalis var. orientalis
322	3	135.08125	34.7875	https://www.gbif.org/occurrence/1830532002	Heloniopsis orientalis var. orientalis
323	4	138.130611	36.887917	https://www.gbif.org/occurrence/1934033275	Heloniopsis orientalis var. orientalis
324	5	138.086667	36.757722	https://www.gbif.org/occurrence/1934033477	Heloniopsis orientalis var. orientalis
325	6	140.081111	35.153611	https://www.gbif.org/occurrence/1933982372	Heloniopsis orientalis var. orientalis
326	7	130.831944	31.510028	https://www.gbif.org/occurrence/1934004866	Heloniopsis orientalis var. orientalis
327	8	137.616667	35.75	https://www.gbif.org/occurrence/1933897837	Heloniopsis orientalis var. orientalis
328	9	134.545472	35.39525	https://www.gbif.org/occurrence/1933982420	Heloniopsis orientalis var. orientalis
329	10	132.845028	35.077667	https://www.gbif.org/occurrence/1934032808	Heloniopsis orientalis var. orientalis
330	11	140.683333	39.45	https://www.gbif.org/occurrence/1933897303	Heloniopsis orientalis var. orientalis
331	12	134.559497	35.401028	https://www.gbif.org/occurrence/1933863715	Heloniopsis orientalis var. orientalis
332	13	132.916667	33.3	https://www.gbif.org/occurrence/1830685148	Heloniopsis orientalis var. orientalis
333	14	133.883333	33.8	https://www.gbif.org/occurrence/1830685168	Heloniopsis orientalis var. orientalis
334	15	132.9	33.3	https://www.gbif.org/occurrence/1830685195	Heloniopsis orientalis var. orientalis
335	16	132.883333	33.266667	https://www.gbif.org/occurrence/1830686192	Heloniopsis orientalis var. orientalis
336	17	133.1	33.533333	https://www.gbif.org/occurrence/1830686361	Heloniopsis orientalis var. orientalis
337	18	136.4476	35.3393	https://www.gbif.org/occurrence/1933838524	Heloniopsis orientalis var. orientalis
338	19	133	33.466667	https://www.gbif.org/occurrence/1830686306	Heloniopsis orientalis var. orientalis
339	20	137.65	35.333333	https://www.gbif.org/occurrence/1933896030	Heloniopsis orientalis var. orientalis
340	21	133.15	33.316667	https://www.gbif.org/occurrence/1830691778	Heloniopsis orientalis var. orientalis
341	22	138.189685	36.829897	https://www.gbif.org/occurrence/1829851024	Heloniopsis orientalis var. orientalis
342	23	135.08299	34.6833	https://www.gbif.org/occurrence/729293524	Heloniopsis orientalis var. orientalis
343	24	136.605425	36.559725	https://www.gbif.org/occurrence/1829850184	Heloniopsis orientalis var. orientalis
344	25	134.633	35.483299	https://www.gbif.org/occurrence/729314974	Heloniopsis orientalis var. orientalis
345	26	134.64999	35.516701	https://www.gbif.org/occurrence/729290420	Heloniopsis orientalis var. orientalis
346	27	135.067	35.016701	https://www.gbif.org/occurrence/729295860	Heloniopsis orientalis var. orientalis
347	28	139.156025	34.215913	https://www.gbif.org/occurrence/388830815	Heloniopsis orientalis var. orientalis
348	29	135.08299	35.049999	https://www.gbif.org/occurrence/729299854	Heloniopsis orientalis var. orientalis
349	30	134.617	35.466702	https://www.gbif.org/occurrence/729315165	Heloniopsis orientalis var. orientalis
350	31	134.58299	35.383301	https://www.gbif.org/occurrence/729318307	Heloniopsis orientalis var. orientalis
351	32	134.517	35.3167	https://www.gbif.org/occurrence/729287148	Heloniopsis orientalis var. orientalis
352	33	135.233	34.75	https://www.gbif.org/occurrence/729279161	Heloniopsis orientalis var. orientalis
353	34	135.25	34.983299	https://www.gbif.org/occurrence/729282238	Heloniopsis orientalis var. orientalis
354	35	135.16701	34.9333	https://www.gbif.org/occurrence/729282721	Heloniopsis orientalis var. orientalis
355	36	135.217	34.783298	https://www.gbif.org/occurrence/729276823	Heloniopsis orientalis var. orientalis
356	37	135.283	34.833302	https://www.gbif.org/occurrence/729295761	Heloniopsis orientalis var. orientalis
357	38	135.283	34.75	https://www.gbif.org/occurrence/729273036	Heloniopsis orientalis var. orientalis
358	39	135.267	34.950001	https://www.gbif.org/occurrence/729282137	Heloniopsis orientalis var. orientalis
359	40	135.33299	34.766701	https://www.gbif.org/occurrence/729294519	Heloniopsis orientalis var. orientalis
360	41	135.283	34.75	https://www.gbif.org/occurrence/729273037	Heloniopsis orientalis var. orientalis
361	42	135.267	34.9333	https://www.gbif.org/occurrence/729282532	Heloniopsis orientalis var. orientalis
362	43	135.233	35.133301	https://www.gbif.org/occurrence/729297561	Heloniopsis orientalis var. orientalis
363	44	135.133	35.099998	https://www.gbif.org/occurrence/729297984	Heloniopsis orientalis var. orientalis
364	45	135.117	35.099998	https://www.gbif.org/occurrence/729297989	Heloniopsis orientalis var. orientalis
365	46	135.14999	34.75	https://www.gbif.org/occurrence/729278165	Heloniopsis orientalis var. orientalis
366	47	135.10001	34.766701	https://www.gbif.org/occurrence/729277941	Heloniopsis orientalis var. orientalis
367	48	135.217	34.766701	https://www.gbif.org/occurrence/729277446	Heloniopsis orientalis var. orientalis
368	49	134.66701	35.033298	https://www.gbif.org/occurrence/729292295	Heloniopsis orientalis var. orientalis
369	50	134.633	35.533298	https://www.gbif.org/occurrence/729310769	Heloniopsis orientalis var. orientalis
370	51	134.633	35.466702	https://www.gbif.org/occurrence/729315116	Heloniopsis orientalis var. orientalis
371	52	135.217	35.002201	https://www.gbif.org/occurrence/729284376	Heloniopsis orientalis var. orientalis
372	53	135	34.5667	https://www.gbif.org/occurrence/729269881	Heloniopsis orientalis var. orientalis
373	54	134.983	34.5667	https://www.gbif.org/occurrence/729298211	Heloniopsis orientalis var. orientalis
374	55	135.267	34.883301	https://www.gbif.org/occurrence/729281095	Heloniopsis orientalis var. orientalis
375	56	134.867	35.083302	https://www.gbif.org/occurrence/729297118	Heloniopsis orientalis var. orientalis
376	57	135.14999	35.051701	https://www.gbif.org/occurrence/729299747	Heloniopsis orientalis var. orientalis
377	58	134.983	35.233299	https://www.gbif.org/occurrence/729311521	Heloniopsis orientalis var. orientalis
378	59	135.10201	35.150299	https://www.gbif.org/occurrence/729319257	Heloniopsis orientalis var. orientalis
379	60	134.517	35.349998	https://www.gbif.org/occurrence/729325683	Heloniopsis orientalis var. orientalis
380	61	135.14999	34.783298	https://www.gbif.org/occurrence/729274852	Heloniopsis orientalis var. orientalis
381	62	135.233	35.75	https://www.gbif.org/occurrence/729273118	Heloniopsis orientalis var. orientalis
382	63	134.7	35.016701	https://www.gbif.org/occurrence/729301429	Heloniopsis orientalis var. orientalis

383	64	133.052393	33.346518	https://www.gbif.org/occurrence/1829858349	Heloniopsis orientalis var. orientalis
384	65	140.5625	36.791667	https://www.gbif.org/occurrence/1934155414	Heloniopsis orientalis var. orientalis
385	66	135.69417	34.73533	https://www.gbif.org/occurrence/1933826892	Heloniopsis orientalis var. orientalis
386	67	135.67741	34.40839	https://www.gbif.org/occurrence/1933906377	Heloniopsis orientalis var. orientalis
387	68	135.67741	34.40839	https://www.gbif.org/occurrence/1933906411	Heloniopsis orientalis var. orientalis
388	69	140.1152	36.89	https://www.gbif.org/occurrence/1934097880	Heloniopsis orientalis var. orientalis
389	70	134.64999	34.883301	https://www.gbif.org/occurrence/729325816	Heloniopsis orientalis var. orientalis
390	71	137.55519	34.91903	https://www.gbif.org/occurrence/1933826923	Heloniopsis orientalis var. orientalis
391	72	137.55519	34.91903	https://www.gbif.org/occurrence/1933826964	Heloniopsis orientalis var. orientalis
392	73	134.79849	35.57761	https://www.gbif.org/occurrence/1933826989	Heloniopsis orientalis var. orientalis
393	74	141.700054	45.034185	https://www.gbif.org/occurrence/388830737	Heloniopsis orientalis var. orientalis
394	75	143.038174	42.082612	https://www.gbif.org/occurrence/388830716	Heloniopsis orientalis var. orientalis
395	76	134.533	35.383301	https://www.gbif.org/occurrence/729318151	Heloniopsis orientalis var. orientalis
396	77	139.588056	33.864444	https://www.gbif.org/occurrence/1829888159	Heloniopsis orientalis var. orientalis
397	78	139.588056	33.864444	https://www.gbif.org/occurrence/1829888394	Heloniopsis orientalis var. orientalis
398	79	139.588056	33.864444	https://www.gbif.org/occurrence/1829888407	Heloniopsis orientalis var. orientalis
399	80	139.164722	34.226667	https://www.gbif.org/occurrence/1829888744	Heloniopsis orientalis var. orientalis
400	81	135.05	35.200001	https://www.gbif.org/occurrence/729311641	Heloniopsis orientalis var. orientalis
401	82	133.531243	35.393488	https://www.gbif.org/occurrence/1829870318	Heloniopsis orientalis var. orientalis
402	83	136.45572	35.15547	https://www.gbif.org/occurrence/1933877850	Heloniopsis orientalis var. orientalis
403	84	134.634	35.468899	https://www.gbif.org/occurrence/729329523	Heloniopsis orientalis var. orientalis
404	85	136.4292	35.019	https://www.gbif.org/occurrence/1933892006	Heloniopsis orientalis var. orientalis
405	86	134.634	35.468899	https://www.gbif.org/occurrence/729327882	Heloniopsis orientalis var. orientalis
406	87	134.634	35.468899	https://www.gbif.org/occurrence/729328811	Heloniopsis orientalis var. orientalis
407	88	134.634	35.468899	https://www.gbif.org/occurrence/729329610	Heloniopsis orientalis var. orientalis
408	89	134.634	35.468899	https://www.gbif.org/occurrence/729328634	Heloniopsis orientalis var. orientalis
409	90	136.45572	35.15547	https://www.gbif.org/occurrence/1933877870	Heloniopsis orientalis var. orientalis
410	91	136.45572	35.15547	https://www.gbif.org/occurrence/1933877875	Heloniopsis orientalis var. orientalis
411	92	139.156025	34.215913	https://www.gbif.org/occurrence/388830582	Heloniopsis orientalis var. orientalis
412	93	141.146667	39.469167	https://www.gbif.org/occurrence/1829902183	Heloniopsis orientalis var. orientalis
413	94	133.544278	35.372861	https://www.gbif.org/occurrence/1829892923	Heloniopsis orientalis var. orientalis
414	95	138.963806	37.316611	https://www.gbif.org/occurrence/1829901462	Heloniopsis orientalis var. orientalis
415	96	138.963806	37.316611	https://www.gbif.org/occurrence/1829901499	Heloniopsis orientalis var. orientalis
416	97	134.764167	34.988333	https://www.gbif.org/occurrence/1829890634	Heloniopsis orientalis var. orientalis
417	98	139.388146	36.865587	https://www.gbif.org/occurrence/388830566	Heloniopsis orientalis var. orientalis
418	99	142.985298	43.779923	https://www.gbif.org/occurrence/388830703	Heloniopsis orientalis var. orientalis
419	100	136.421228	35.001237	https://www.gbif.org/occurrence/1829824191	Heloniopsis orientalis var. orientalis
420	101	139.910378	39.194088	https://www.gbif.org/occurrence/1829818123	Heloniopsis orientalis var. orientalis
421	102	137.8625	36.698056	https://www.gbif.org/occurrence/1829899738	Heloniopsis orientalis var. orientalis
422	103	139.3902	35.5762	https://www.gbif.org/occurrence/1934061691	Heloniopsis orientalis var. orientalis
423	104	138.456667	35.768889	https://www.gbif.org/occurrence/1829896111	Heloniopsis orientalis var. orientalis
424	105	142.854204	43.663542	https://www.gbif.org/occurrence/388830587	Heloniopsis orientalis var. orientalis
425	106	140.002643	37.619764	https://www.gbif.org/occurrence/1829825264	Heloniopsis orientalis var. orientalis
426	107	138.645315	36.410905	https://www.gbif.org/occurrence/1829823866	Heloniopsis orientalis var. orientalis
427	108	138.177958	36.100541	https://www.gbif.org/occurrence/1829824351	Heloniopsis orientalis var. orientalis
428	109	138.157313	35.461261	https://www.gbif.org/occurrence/1829824184	Heloniopsis orientalis var. orientalis
429	110	139.173198	36.903438	https://www.gbif.org/occurrence/1829824158	Heloniopsis orientalis var. orientalis
430	111	139.173198	36.903438	https://www.gbif.org/occurrence/1829824175	Heloniopsis orientalis var. orientalis
431	112	139.203275	36.918628	https://www.gbif.org/occurrence/1829824376	Heloniopsis orientalis var. orientalis
432	113	139.173198	36.903438	https://www.gbif.org/occurrence/1829825266	Heloniopsis orientalis var. orientalis
433	114	140.47931	38.24942	https://www.gbif.org/occurrence/1933826886	Heloniopsis orientalis var. orientalis
434	115	140.15555	35.159	https://www.gbif.org/occurrence/1933873370	Heloniopsis orientalis var. orientalis
435	116	138.9302	36.8344	https://www.gbif.org/occurrence/1934153508	Heloniopsis orientalis var. orientalis
436	117	138.9302	36.8344	https://www.gbif.org/occurrence/1934153512	Heloniopsis orientalis var. orientalis
437	118	137.6295	36.152	https://www.gbif.org/occurrence/1934061698	Heloniopsis orientalis var. orientalis
438	119	139.0413	35.1277	https://www.gbif.org/occurrence/1934061716	Heloniopsis orientalis var. orientalis
439	120	139.0413	35.1277	https://www.gbif.org/occurrence/1934061726	Heloniopsis orientalis var. orientalis
440	121	137.6182	36.5793	https://www.gbif.org/occurrence/1933977001	Heloniopsis orientalis var. orientalis
441	1	103.44424	30.587229	https://www.gbif.org/occurrence/1030871249	Ypsilandra thibetica
442	2	110.85	26.43	https://www.gbif.org/occurrence/2417614381	Ypsilandra thibetica
443	3	102.928376	30.144125	https://www.gbif.org/occurrence/1030898355	Ypsilandra thibetica
444	4	102.928376	30.144125	https://www.gbif.org/occurrence/1030898380	Ypsilandra thibetica
445	5	102.758313	30.066708	https://www.gbif.org/occurrence/1030871210	Ypsilandra thibetica
446	6	102.758313	30.066708	https://www.gbif.org/occurrence/1030871229	Ypsilandra thibetica
447	7	102.758313	30.066708	https://www.gbif.org/occurrence/1030871230	Ypsilandra thibetica
448	8	110.585686	25.60168	https://www.gbif.org/occurrence/1030923728	Ypsilandra thibetica
449	9	110.585686	25.60168	https://www.gbif.org/occurrence/1030923733	Ypsilandra thibetica
450	10	110.585686	25.60168	https://www.gbif.org/occurrence/1030923749	Ypsilandra thibetica
451	11	110.585686	25.60168	https://www.gbif.org/occurrence/1030923764	Ypsilandra thibetica
452	12	102.758313	30.066708	https://www.gbif.org/occurrence/1030870998	Ypsilandra thibetica
453	13	102.758313	30.066708	https://www.gbif.org/occurrence/1030898353	Ypsilandra thibetica
454	14	102.758313	30.066708	https://www.gbif.org/occurrence/1030898365	Ypsilandra thibetica
455	15	103.571742	28.262715	https://www.gbif.org/occurrence/1030898372	Ypsilandra thibetica
456	16	103.39	29.49	https://www.gbif.org/occurrence/2417519251	Ypsilandra thibetica
457	17	103.39	29.49	https://www.gbif.org/occurrence/2417520984	Ypsilandra thibetica
458	18	107.099276	29.157849	https://www.gbif.org/occurrence/1030898397	Ypsilandra thibetica
459	19	103.39	29.49	https://www.gbif.org/occurrence/2417517659	Ypsilandra thibetica

460	20	103.39	29.49	https://www.gbif.org/occurrence/2417525168	Ypsilandra thibetica
461	21	103.39	29.49	https://www.gbif.org/occurrence/2417517470	Ypsilandra thibetica
462	22	103.39	29.49	https://www.gbif.org/occurrence/2417518242	Ypsilandra thibetica
463	23	110.040007	25.742153	https://www.gbif.org/occurrence/1030951317	Ypsilandra thibetica
464	24	110.040007	25.742153	https://www.gbif.org/occurrence/1030951303	Ypsilandra thibetica
465	25	102.81464	30.368117	https://www.gbif.org/occurrence/1030870952	Ypsilandra thibetica
466	26	102.758313	30.066708	https://www.gbif.org/occurrence/1030898317	Ypsilandra thibetica
467	27	102.990174	32.061655	https://www.gbif.org/occurrence/1030898345	Ypsilandra thibetica
468	28	103.398589	29.496119	https://www.gbif.org/occurrence/1030898347	Ypsilandra thibetica
469	29	102.81464	30.368117	https://www.gbif.org/occurrence/1030898352	Ypsilandra thibetica
470	30	102.758313	30.066708	https://www.gbif.org/occurrence/1030898367	Ypsilandra thibetica
471	31	103.398589	29.496119	https://www.gbif.org/occurrence/1030898374	Ypsilandra thibetica
472	32	102.81464	30.368117	https://www.gbif.org/occurrence/1030898376	Ypsilandra thibetica
473	33	103.398589	29.496119	https://www.gbif.org/occurrence/1030898377	Ypsilandra thibetica
474	34	109.833841	27.203906	https://www.gbif.org/occurrence/1030951283	Ypsilandra thibetica
475	35	105.1	28.27	https://www.gbif.org/occurrence/2417341998	Ypsilandra thibetica
476	36	105.1	28.27	https://www.gbif.org/occurrence/2417346086	Ypsilandra thibetica
477	37	102.75	30.06	https://www.gbif.org/occurrence/2417441556	Ypsilandra thibetica
478	38	102.81	30.36	https://www.gbif.org/occurrence/2417487624	Ypsilandra thibetica
479	39	103.39	29.49	https://www.gbif.org/occurrence/2417519887	Ypsilandra thibetica
480	40	103.39	29.49	https://www.gbif.org/occurrence/2417519975	Ypsilandra thibetica
481	41	103.39	29.49	https://www.gbif.org/occurrence/2417526537	Ypsilandra thibetica
482	42	103.39	29.49	https://www.gbif.org/occurrence/2417520516	Ypsilandra thibetica
483	43	103.39	29.49	https://www.gbif.org/occurrence/2417521206	Ypsilandra thibetica
484	44	110.040007	25.742153	https://www.gbif.org/occurrence/1030951323	Ypsilandra thibetica
485	45	103.39	29.49	https://www.gbif.org/occurrence/2417518784	Ypsilandra thibetica
486	46	103.39	29.49	https://www.gbif.org/occurrence/2417519133	Ypsilandra thibetica
487	47	103.39	29.49	https://www.gbif.org/occurrence/2417521630	Ypsilandra thibetica
488	48	109.83	27.2	https://www.gbif.org/occurrence/2417711465	Ypsilandra thibetica
489	49	102.758313	30.066708	https://www.gbif.org/occurrence/1030951261	Ypsilandra thibetica
490	50	110.050532	25.255089	https://www.gbif.org/occurrence/1030951281	Ypsilandra thibetica
491	51	110.050532	25.255089	https://www.gbif.org/occurrence/1030951294	Ypsilandra thibetica
492	52	102.758313	30.066708	https://www.gbif.org/occurrence/1030951297	Ypsilandra thibetica
493	53	102.758313	30.066708	https://www.gbif.org/occurrence/1030951305	Ypsilandra thibetica
494	54	110.05	25.25	https://www.gbif.org/occurrence/2417280201	Ypsilandra thibetica
495	55	110.58	25.6	https://www.gbif.org/occurrence/2417341103	Ypsilandra thibetica
496	56	102.75	30.06	https://www.gbif.org/occurrence/2417440195	Ypsilandra thibetica
497	57	102.75	30.06	https://www.gbif.org/occurrence/2417441755	Ypsilandra thibetica
498	58	102.75	30.06	https://www.gbif.org/occurrence/2417442085	Ypsilandra thibetica
499	59	102.75	30.06	https://www.gbif.org/occurrence/2417442557	Ypsilandra thibetica
500	60	102.75	30.06	https://www.gbif.org/occurrence/2417445188	Ypsilandra thibetica
501	61	103.398589	29.496119	https://www.gbif.org/occurrence/1030951265	Ypsilandra thibetica
502	62	103.853481	31.68115	https://www.gbif.org/occurrence/1030951313	Ypsilandra thibetica
503	63	103.39	29.49	https://www.gbif.org/occurrence/2417518529	Ypsilandra thibetica
504	64	103.39	29.49	https://www.gbif.org/occurrence/2417518579	Ypsilandra thibetica
505	65	103.39	29.49	https://www.gbif.org/occurrence/2417518590	Ypsilandra thibetica
506	66	103.39	29.49	https://www.gbif.org/occurrence/2417519324	Ypsilandra thibetica
507	67	103.39	29.49	https://www.gbif.org/occurrence/2417523280	Ypsilandra thibetica
508	68	102.54	30.91	https://www.gbif.org/occurrence/2417736736	Ypsilandra thibetica
509	69	104.39	34.04	https://www.gbif.org/occurrence/2417475368	Ypsilandra thibetica
510	70	112.948819	25.39996	https://www.gbif.org/occurrence/1030951285	Ypsilandra thibetica
511	71	103.398589	29.496119	https://www.gbif.org/occurrence/1303703889	Ypsilandra thibetica
512	72	103.398589	29.496119	https://www.gbif.org/occurrence/1303703900	Ypsilandra thibetica
513	73	103.398589	29.496119	https://www.gbif.org/occurrence/1303965973	Ypsilandra thibetica
514	74	103.398589	29.496119	https://www.gbif.org/occurrence/1303965991	Ypsilandra thibetica
515	75	112.948819	25.39996	https://www.gbif.org/occurrence/1303966034	Ypsilandra thibetica
516	76	103.398589	29.496119	https://www.gbif.org/occurrence/1303703906	Ypsilandra thibetica
517	77	103.398589	29.496119	https://www.gbif.org/occurrence/1303965957	Ypsilandra thibetica
518	78	103.398589	29.496119	https://www.gbif.org/occurrence/1303966540	Ypsilandra thibetica
519	79	103.398589	29.496119	https://www.gbif.org/occurrence/1303703843	Ypsilandra thibetica
520	80	103.398589	29.496119	https://www.gbif.org/occurrence/1303703886	Ypsilandra thibetica
521	81	103.398589	29.496119	https://www.gbif.org/occurrence/1304026760	Ypsilandra thibetica
522	82	103.398589	29.496119	https://www.gbif.org/occurrence/1304026773	Ypsilandra thibetica
523	83	103.398589	29.496119	https://www.gbif.org/occurrence/1304026796	Ypsilandra thibetica
524	84	103.231884	29.73797	https://www.gbif.org/occurrence/1303703818	Ypsilandra thibetica
525	85	103.231884	29.73797	https://www.gbif.org/occurrence/1303703836	Ypsilandra thibetica
526	86	103.398589	29.496119	https://www.gbif.org/occurrence/1303703845	Ypsilandra thibetica
527	87	103.398589	29.496119	https://www.gbif.org/occurrence/1303703871	Ypsilandra thibetica
528	88	113.184435	24.813781	https://www.gbif.org/occurrence/1030951295	Ypsilandra thibetica
529	89	107.099276	29.157849	https://www.gbif.org/occurrence/1303703870	Ypsilandra thibetica
530	90	102.7586	30.066425	https://www.gbif.org/occurrence/1057119797	Ypsilandra thibetica
531	91	111.009947	25.897942	https://www.gbif.org/occurrence/1030951316	Ypsilandra thibetica
532	92	102.758313	30.066708	https://www.gbif.org/occurrence/1303703819	Ypsilandra thibetica
533	93	111.009947	25.897942	https://www.gbif.org/occurrence/1303703833	Ypsilandra thibetica
534	94	102.758313	30.066708	https://www.gbif.org/occurrence/1303703841	Ypsilandra thibetica
535	95	102.758313	30.066708	https://www.gbif.org/occurrence/1303703862	Ypsilandra thibetica
536	96	102.758313	30.066708	https://www.gbif.org/occurrence/1303703864	Ypsilandra thibetica

537	97	102.758313	30.066708	https://www.gbif.org/occurrence/1303703876	Ypsilandra thibetica
538	98	102.758313	30.066708	https://www.gbif.org/occurrence/1303703881	Ypsilandra thibetica
539	99	102.758313	30.066708	https://www.gbif.org/occurrence/1303703902	Ypsilandra thibetica
540	100	102.758313	30.066708	https://www.gbif.org/occurrence/1303703924	Ypsilandra thibetica
541	101	106.761103	26.543493	https://www.gbif.org/occurrence/1303965908	Ypsilandra thibetica
542	102	111.009947	25.897942	https://www.gbif.org/occurrence/1303965974	Ypsilandra thibetica
543	103	102.758313	30.066708	https://www.gbif.org/occurrence/1303965976	Ypsilandra thibetica
544	104	102.758313	30.066708	https://www.gbif.org/occurrence/1303965984	Ypsilandra thibetica
545	105	102.758313	30.066708	https://www.gbif.org/occurrence/1303965988	Ypsilandra thibetica
546	106	107.099276	29.157849	https://www.gbif.org/occurrence/1303703812	Ypsilandra thibetica
547	107	107.099276	29.157849	https://www.gbif.org/occurrence/1303703913	Ypsilandra thibetica
548	108	106.266667	29.3	https://www.gbif.org/occurrence/1303703877	Ypsilandra thibetica
549	109	103.398589	29.496119	https://www.gbif.org/occurrence/1303703861	Ypsilandra thibetica
550	110	107.099276	29.157849	https://www.gbif.org/occurrence/1303703863	Ypsilandra thibetica
551	111	103.398589	29.496119	https://www.gbif.org/occurrence/1303703873	Ypsilandra thibetica
552	112	103.398589	29.496119	https://www.gbif.org/occurrence/1303703887	Ypsilandra thibetica
553	113	107.099276	29.157849	https://www.gbif.org/occurrence/1303703909	Ypsilandra thibetica
554	114	107.099276	29.157849	https://www.gbif.org/occurrence/1303703934	Ypsilandra thibetica
555	115	107.099276	29.157849	https://www.gbif.org/occurrence/1303966544	Ypsilandra thibetica
556	1	98.460833	27.784444	https://www.gbif.org/occurrence/1426119922	Ypsilandra yunnanensis
557	2	98.459167	27.753889	https://www.gbif.org/occurrence/686866892	Ypsilandra yunnanensis
558	3	98.460833	27.784444	https://www.gbif.org/occurrence/723808433	Ypsilandra yunnanensis
559	4	98.702194	27.215723	https://www.gbif.org/occurrence/1426073456	Ypsilandra yunnanensis
560	5	98.7015	27.214083	https://www.gbif.org/occurrence/1426099879	Ypsilandra yunnanensis
561	6	98.694138	27.199413	https://www.gbif.org/occurrence/1426118453	Ypsilandra yunnanensis
562	7	98.695885	27.210333	https://www.gbif.org/occurrence/1426120200	Ypsilandra yunnanensis
563	8	98.694695	27.200806	https://www.gbif.org/occurrence/1426219333	Ypsilandra yunnanensis
564	9	98.695854	27.212278	https://www.gbif.org/occurrence/1426069215	Ypsilandra yunnanensis
565	10	98.720062	27.18494	https://www.gbif.org/occurrence/1426119771	Ypsilandra yunnanensis
566	11	98.695833	27.212222	https://www.gbif.org/occurrence/575203234	Ypsilandra yunnanensis
567	12	98.465919	27.793079	https://www.gbif.org/occurrence/1426107943	Ypsilandra yunnanensis
568	13	98.465833	27.793056	https://www.gbif.org/occurrence/890157378	Ypsilandra yunnanensis
569	14	98.455276	27.696108	https://www.gbif.org/occurrence/1426055606	Ypsilandra yunnanensis
570	15	98.491661	27.713055	https://www.gbif.org/occurrence/1426213133	Ypsilandra yunnanensis
571	16	98.4553	27.6961	https://www.gbif.org/occurrence/147060139	Ypsilandra yunnanensis
572	17	98.4917	27.7131	https://www.gbif.org/occurrence/147060142	Ypsilandra yunnanensis
573	18	98.491667	27.713056	https://www.gbif.org/occurrence/575151234	Ypsilandra yunnanensis
574	19	87.17	27.67	https://www.gbif.org/occurrence/1056740185	Ypsilandra yunnanensis
575	20	99.28717	27.177166	https://www.gbif.org/occurrence/919464584	Ypsilandra yunnanensis
576	21	99.28717	27.177166	https://www.gbif.org/occurrence/919464591	Ypsilandra yunnanensis
577	22	95.76	29.85	https://www.gbif.org/occurrence/2417697019	Ypsilandra yunnanensis
578	23	95.76	29.85	https://www.gbif.org/occurrence/2417697405	Ypsilandra yunnanensis
579	24	95.76	29.85	https://www.gbif.org/occurrence/2417699046	Ypsilandra yunnanensis
580	25	95.76	29.85	https://www.gbif.org/occurrence/2417699435	Ypsilandra yunnanensis
581	26	95.76	29.85	https://www.gbif.org/occurrence/2417699456	Ypsilandra yunnanensis
582	27	98.66582	27.740736	https://www.gbif.org/occurrence/919531270	Ypsilandra yunnanensis
583	28	98.66582	27.740736	https://www.gbif.org/occurrence/919531280	Ypsilandra yunnanensis
584	29	98.66582	27.740736	https://www.gbif.org/occurrence/919531291	Ypsilandra yunnanensis
585	30	83.938548	28.253008	https://www.gbif.org/occurrence/1057508184	Ypsilandra yunnanensis
586	31	91.208	27.767	https://www.gbif.org/occurrence/1057083648	Ypsilandra yunnanensis
587	32	98.911543	28.48611	https://www.gbif.org/occurrence/1304026789	Ypsilandra yunnanensis
588	33	98.911543	28.48611	https://www.gbif.org/occurrence/1304026790	Ypsilandra yunnanensis
589	34	98.911543	28.48611	https://www.gbif.org/occurrence/1304026802	Ypsilandra yunnanensis
590	35	98.66582	27.740736	https://www.gbif.org/occurrence/1304026803	Ypsilandra yunnanensis
591	36	98.911543	28.48611	https://www.gbif.org/occurrence/1304026812	Ypsilandra yunnanensis
592	37	98.911543	28.48611	https://www.gbif.org/occurrence/1304026828	Ypsilandra yunnanensis
593	38	98.66582	27.740736	https://www.gbif.org/occurrence/1304026929	Ypsilandra yunnanensis
594	39	98.911543	28.48611	https://www.gbif.org/occurrence/919531268	Ypsilandra yunnanensis
595	40	98.911543	28.48611	https://www.gbif.org/occurrence/919531273	Ypsilandra yunnanensis
596	41	98.911543	28.48611	https://www.gbif.org/occurrence/919531283	Ypsilandra yunnanensis
597	42	98.911543	28.48611	https://www.gbif.org/occurrence/919531290	Ypsilandra yunnanensis
598	43	98.911543	28.48611	https://www.gbif.org/occurrence/919531294	Ypsilandra yunnanensis
599	44	98.911543	28.48611	https://www.gbif.org/occurrence/919531307	Ypsilandra yunnanensis
600	45	98.911543	28.48611	https://www.gbif.org/occurrence/919588795	Ypsilandra yunnanensis
601	46	98.66582	27.740736	https://www.gbif.org/occurrence/1304026758	Ypsilandra yunnanensis
602	47	98.66582	27.740736	https://www.gbif.org/occurrence/1304026779	Ypsilandra yunnanensis
603	48	98.66582	27.740736	https://www.gbif.org/occurrence/1304026795	Ypsilandra yunnanensis
604	49	98.66582	27.740736	https://www.gbif.org/occurrence/1304026944	Ypsilandra yunnanensis
605	50	98.66582	27.740736	https://www.gbif.org/occurrence/919531251	Ypsilandra yunnanensis
606	51	98.66582	27.740736	https://www.gbif.org/occurrence/919531261	Ypsilandra yunnanensis
607	52	98.66582	27.740736	https://www.gbif.org/occurrence/919531313	Ypsilandra yunnanensis
608	53	98.911543	28.48611	https://www.gbif.org/occurrence/919531322	Ypsilandra yunnanensis
609	54	98.911543	28.48611	https://www.gbif.org/occurrence/919588794	Ypsilandra yunnanensis
610	55	98.66582	27.740736	https://www.gbif.org/occurrence/1304026756	Ypsilandra yunnanensis
611	56	103.891767	36.519977	https://www.gbif.org/occurrence/1055801870	Ypsilandra yunnanensis
612	57	97.67	28.33	https://www.gbif.org/occurrence/1056367875	Ypsilandra yunnanensis
613	1	35.352	127.5351	https://www.naturing.net/o/359138	Heloniopsis tubiflora

614	2	35.29662	127.53005	https://www.naturing.net/o/323839	Heloniopsis tubiflora
615	3	35.29599	127.53	https://www.naturing.net/o/471003	Heloniopsis tubiflora
616	4	35.84853	127.74556	https://www.naturing.net/o/326509	Heloniopsis tubiflora
617	5	35.85802	127.7464	https://www.naturing.net/o/8670	Heloniopsis tubiflora
618	6	35.85802	127.74661	https://www.naturing.net/o/297506	Heloniopsis tubiflora
619	7	37.11262	128.92667	https://www.naturing.net/o/79487	Heloniopsis tubiflora
620	8	35.54118	129.05865	https://www.naturing.net/o/98475	Heloniopsis tubiflora
621	9	35.81327	127.72972	https://www.naturing.net/o/81929	Heloniopsis tubiflora
622	10	38.14663	127.48105	herbarium specimen	Heloniopsis tubiflora
623	11	35.309549	127.579089	https://www.gbif.org/occurrence/2609423503	Heloniopsis tubiflora
624	12	38.17	128.493465	https://www.gbif.org/occurrence/2609423797	Heloniopsis tubiflora
625	13	35.341342	127.734417	https://www.gbif.org/occurrence/2609424475	Heloniopsis tubiflora
626	1	28.28847642	129.3157555	Tanaka, 2019,RYU-25059	Helonias kawanoi
627	2	24.76934881	125.3241704	Tanaka, 2019,TNS-60738	Helonias kawanoi
628	3	26.71384287	128.2689202	Tanaka, 2019,L-1455529*, TI	Helonias kawanoi
629	4	24.3360742	123.8171499	Tanaka, 2019,MAK-141694, RYU-17010	Helonias kawanoi
630	5	24.32474229	123.7825761	Tanaka, 2019,TNS-60736, -60737	Helonias kawanoi
631	6	24.31639647	123.8194057	Tanaka, 2019,TI, RYU-27147	Helonias kawanoi
632	7	24.42811651	124.1834577	Tanaka, 2019,MAK-138905	Helonias kawanoi
633	1	26.66787791	128.1626844	Tanaka, 2019, TNS-129705	Heloniopsis leucantha
634	2	26.61617043	128.0433308	Tanaka, 2019, RYU-41828; TNS-116547, 116548).	Heloniopsis leucantha
635	3	24.36323668	123.7959738	Tanaka, 2019, TI	Heloniopsis leucantha
636	4	24.40729251	124.1755926	Tanaka, 2019, RYU-19301	Heloniopsis leucantha
637	1	23.68944	120.90722	This research	Heloniopsis sp.
638	2	24.6213	120.80412	This research	Heloniopsis sp.
639	3	23.66451	120.7704	This research	Heloniopsis sp.
640	4	24.01805	121.36916	This research	Heloniopsis sp.

Supplementary Table S8. Data of anthesis temperature for ANOVA analysis

	name	mon	temp (10°C)
1	Heloniopsis koreana	3	31
2	Heloniopsis koreana	4	89
3	Heloniopsis koreana	4	97
4	Heloniopsis koreana	4	97
5	Heloniopsis koreana	4	112
6	Heloniopsis koreana	4	97
7	Heloniopsis koreana	5	100
8	Heloniopsis koreana	5	105
9	Heloniopsis koreana	5	165
10	Heloniopsis koreana	5	169
11	Heloniopsis koreana	5	108
12	Heloniopsis koreana	5	114
13	Heloniopsis koreana	5	155
14	Heloniopsis koreana	5	130
15	Heloniopsis koreana	4	117
16	Heloniopsis koreana	4	75
17	Heloniopsis koreana	4	100
18	Heloniopsis koreana	5	173
19	Heloniopsis koreana	4	106
20	Heloniopsis koreana	4	100
21	Heloniopsis koreana	4	116
22	Heloniopsis koreana	4	116
23	Heloniopsis koreana	5	173
24	Heloniopsis koreana	5	173
25	Heloniopsis koreana	5	101
26	Heloniopsis koreana	5	152
27	Heloniopsis koreana	5	158
28	Heloniopsis koreana	5	152
29	Heloniopsis koreana	5	152
30	Heloniopsis koreana	5	152
31	Heloniopsis koreana	5	152
32	Heloniopsis koreana	5	146
33	Heloniopsis orientalis var. breviscapa	4	103
34	Heloniopsis orientalis var. breviscapa	5	137
35	Heloniopsis orientalis var. breviscapa	5	165
36	Heloniopsis orientalis var. breviscapa	6	183
37	Heloniopsis orientalis var. breviscapa	6	183
38	Heloniopsis orientalis var. breviscapa	3	93
39	Heloniopsis orientalis var. breviscapa	3	105
40	Heloniopsis orientalis var. breviscapa	4	122

41	Heloniopsis orientalis var. breviscapa	5	155
42	Heloniopsis orientalis var. breviscapa	5	161
43	Heloniopsis orientalis var. breviscapa	4	103
44	Heloniopsis orientalis var. breviscapa	5	137
45	Heloniopsis orientalis var. breviscapa	3	81
46	Heloniopsis orientalis var. breviscapa	5	165
47	Heloniopsis orientalis var. breviscapa	6	183
48	Heloniopsis orientalis var. breviscapa	6	183
49	Heloniopsis orientalis var. breviscapa	9	203
50	Heloniopsis orientalis var. breviscapa	3	93
51	Heloniopsis orientalis var. breviscapa	3	105
52	Heloniopsis orientalis var. breviscapa	4	169
53	Heloniopsis orientalis var. breviscapa	4	122
54	Heloniopsis orientalis var. breviscapa	6	196
55	Heloniopsis orientalis var. breviscapa	3	85
56	Heloniopsis orientalis var. breviscapa	4	138
57	Heloniopsis orientalis var. breviscapa	4	150
58	Heloniopsis orientalis var. breviscapa	4	138
59	Heloniopsis orientalis var. breviscapa	5	155
60	Heloniopsis orientalis var. breviscapa	7	264
61	Heloniopsis orientalis var. breviscapa	4	152
62	Heloniopsis orientalis var. breviscapa	3	98
63	Heloniopsis orientalis var. breviscapa	5	179
64	Heloniopsis orientalis var. breviscapa	5	179
65	Heloniopsis orientalis var. breviscapa	5	184
66	Heloniopsis orientalis var. breviscapa	3	80
67	Heloniopsis orientalis var. breviscapa	3	104
68	Heloniopsis orientalis var. breviscapa	4	149
69	Heloniopsis orientalis var. breviscapa	5	161
70	Heloniopsis orientalis var. breviscapa	3	91
71	Heloniopsis orientalis var. breviscapa	3	84
72	Heloniopsis orientalis var. breviscapa	3	107
73	Heloniopsis orientalis var. breviscapa	3	87
74	Heloniopsis orientalis var. breviscapa	4	146
75	Heloniopsis orientalis var. breviscapa	4	151
76	Heloniopsis orientalis var. breviscapa	4	140
77	Heloniopsis orientalis var. breviscapa	3	99
78	Heloniopsis orientalis var. breviscapa	5	159
79	Heloniopsis orientalis var. breviscapa	5	159
80	Heloniopsis orientalis var. breviscapa	3	87
81	Heloniopsis orientalis var. breviscapa	3	87
82	Heloniopsis orientalis var. breviscapa	3	87
83	Heloniopsis orientalis var. breviscapa	4	132

84	Heloniopsis orientalis var. breviscapa	4	132
85	Heloniopsis orientalis var. breviscapa	8	213
86	Heloniopsis orientalis var. breviscapa	3	84
87	Heloniopsis orientalis var. breviscapa	5	167
88	Heloniopsis orientalis var. breviscapa	5	167
89	Heloniopsis orientalis var. breviscapa	5	172
90	Heloniopsis orientalis var. breviscapa	4	132
91	Heloniopsis orientalis var. breviscapa	4	127
92	Heloniopsis orientalis var. breviscapa	4	127
93	Heloniopsis orientalis var. breviscapa	4	132
94	Heloniopsis orientalis var. breviscapa	4	132
95	Heloniopsis orientalis var. breviscapa	4	135
96	Heloniopsis orientalis var. breviscapa	6	149
97	Heloniopsis orientalis var. breviscapa	3	86
98	Heloniopsis orientalis var. breviscapa	4	135
99	Heloniopsis orientalis var. breviscapa	4	141
100	Heloniopsis orientalis var. breviscapa	4	164
101	Heloniopsis orientalis var. breviscapa	4	164
102	Heloniopsis orientalis var. breviscapa	4	172
103	Heloniopsis orientalis var. breviscapa	4	172
104	Heloniopsis orientalis var. breviscapa	5	188
105	Heloniopsis orientalis var. breviscapa	3	75
106	Heloniopsis orientalis var. breviscapa	4	141
107	Heloniopsis orientalis var. breviscapa	5	172
108	Heloniopsis orientalis var. breviscapa	4	132
109	Heloniopsis orientalis var. breviscapa	4	132
110	Heloniopsis orientalis var. breviscapa	4	132
111	Heloniopsis orientalis var. breviscapa	5	172
112	Heloniopsis orientalis var. breviscapa	5	172
113	Heloniopsis orientalis var. breviscapa	5	172
114	Heloniopsis orientalis var. breviscapa	4	141
115	Heloniopsis orientalis var. breviscapa	6	171
116	Heloniopsis orientalis var. breviscapa	4	104
117	Heloniopsis orientalis var. breviscapa	4	132
118	Heloniopsis orientalis var. flavida	4	110
119	Heloniopsis orientalis var. flavida	3	87
120	Heloniopsis orientalis var. flavida	12	41
121	Heloniopsis orientalis var. flavida	4	121
122	Heloniopsis orientalis var. flavida	4	131
123	Heloniopsis orientalis var. flavida	5	130
124	Heloniopsis orientalis var. flavida	3	76
125	Heloniopsis orientalis var. flavida	3	75
126	Heloniopsis orientalis var. flavida	4	123

127	Heloniopsis orientalis var. flavida	4	122
128	Heloniopsis orientalis var. flavida	4	123
129	Heloniopsis orientalis var. flavida	3	80
130	Heloniopsis orientalis var. flavida	3	73
131	Heloniopsis orientalis var. flavida	4	132
132	Heloniopsis orientalis var. flavida	4	116
133	Heloniopsis orientalis var. flavida	4	128
134	Heloniopsis orientalis var. flavida	4	137
135	Heloniopsis orientalis var. flavida	4	136
136	Heloniopsis orientalis var. flavida	4	116
137	Heloniopsis orientalis var. flavida	10	118
138	Heloniopsis orientalis var. flavida	3	89
139	Heloniopsis orientalis var. flavida	3	85
140	Heloniopsis orientalis var. flavida	3	74
141	Heloniopsis orientalis var. flavida	4	147
142	Heloniopsis orientalis var. flavida	5	155
143	Heloniopsis orientalis var. flavida	4	103
144	Heloniopsis orientalis var. flavida	4	91
145	Heloniopsis orientalis var. flavida	5	179
146	Heloniopsis orientalis var. flavida	3	86
147	Heloniopsis orientalis var. flavida	4	134
148	Heloniopsis orientalis var. flavida	10	153
149	Heloniopsis orientalis var. flavida	4	103
150	Heloniopsis orientalis var. flavida	4	103
151	Heloniopsis orientalis var. flavida	5	137
152	Heloniopsis orientalis var. flavida	6	232
153	Heloniopsis orientalis var. flavida	6	181
154	Heloniopsis orientalis var. flavida	6	196
155	Heloniopsis orientalis var. flavida	4	125
156	Heloniopsis orientalis var. flavida	4	148
157	Heloniopsis orientalis var. flavida	4	104
158	Heloniopsis orientalis var. flavida	5	170
159	Heloniopsis orientalis var. flavida	3	82
160	Heloniopsis orientalis var. flavida	4	147
161	Heloniopsis orientalis var. flavida	4	148
162	Heloniopsis orientalis var. flavida	4	125
163	Heloniopsis orientalis var. flavida	4	112
164	Heloniopsis orientalis var. flavida	4	138
165	Heloniopsis orientalis var. flavida	5	178
166	Heloniopsis orientalis var. flavida	5	167
167	Heloniopsis orientalis var. flavida	5	153
168	Heloniopsis orientalis var. flavida	5	184
169	Heloniopsis orientalis var. flavida	5	192

170	Heloniopsis orientalis var. flavida	6	195
171	Heloniopsis orientalis var. flavida	6	194
172	Heloniopsis orientalis var. flavida	3	75
173	Heloniopsis orientalis var. flavida	4	148
174	Heloniopsis orientalis var. flavida	5	187
175	Heloniopsis orientalis var. flavida	5	151
176	Heloniopsis orientalis var. flavida	5	151
177	Heloniopsis orientalis var. flavida	5	168
178	Heloniopsis orientalis var. flavida	5	161
179	Heloniopsis orientalis var. flavida	8	220
180	Heloniopsis orientalis var. flavida	10	141
181	Heloniopsis orientalis var. flavida	3	94
182	Heloniopsis orientalis var. flavida	3	96
183	Heloniopsis orientalis var. flavida	5	164
184	Heloniopsis orientalis var. flavida	4	148
185	Heloniopsis orientalis var. flavida	4	124
186	Heloniopsis orientalis var. flavida	4	109
187	Heloniopsis orientalis var. flavida	5	182
188	Heloniopsis orientalis var. flavida	6	171
189	Heloniopsis orientalis var. flavida	4	133
190	Heloniopsis orientalis var. flavida	4	126
191	Heloniopsis orientalis var. flavida	4	126
192	Heloniopsis orientalis var. flavida	3	85
193	Heloniopsis orientalis var. flavida	4	108
194	Heloniopsis orientalis var. flavida	5	115
195	Heloniopsis orientalis var. flavida	7	208
196	Heloniopsis orientalis var. flavida	4	106
197	Heloniopsis orientalis var. flavida	4	128
198	Heloniopsis orientalis var. flavida	4	160
199	Heloniopsis orientalis var. flavida	6	152
200	Heloniopsis orientalis var. flavida	4	89
201	Heloniopsis orientalis var. flavida	7	271
202	Heloniopsis orientalis var. flavida	3	56
203	Heloniopsis orientalis var. flavida	3	113
204	Heloniopsis orientalis var. flavida	4	128
205	Heloniopsis orientalis var. flavida	4	128
206	Heloniopsis orientalis var. flavida	4	128
207	Heloniopsis orientalis var. flavida	4	77
208	Heloniopsis orientalis var. flavida	3	56
209	Heloniopsis orientalis var. flavida	4	121
210	Heloniopsis orientalis var. flavida	4	128
211	Heloniopsis orientalis var. flavida	4	123
212	Heloniopsis orientalis var. flavida	4	105

213	Heloniopsis orientalis var. flavida	4	105
214	Heloniopsis orientalis var. flavida	4	160
215	Heloniopsis orientalis var. flavida	5	120
216	Heloniopsis orientalis var. flavida	3	81
217	Heloniopsis orientalis var. flavida	8	283
218	Heloniopsis orientalis var. flavida	3	111
219	Heloniopsis orientalis var. flavida	4	138
220	Heloniopsis orientalis var. flavida	4	123
221	Heloniopsis orientalis var. flavida	5	200
222	Heloniopsis orientalis var. flavida	5	200
223	Heloniopsis orientalis var. flavida	8	283
224	Heloniopsis orientalis var. flavida	8	284
225	Heloniopsis orientalis var. flavida	4	117
226	Heloniopsis orientalis var. flavida	8	281
227	Heloniopsis orientalis var. flavida	11	151
228	Heloniopsis orientalis var. flavida	3	102
229	Heloniopsis orientalis var. flavida	3	102
230	Heloniopsis orientalis var. flavida	5	200
231	Heloniopsis orientalis var. flavida	7	244
232	Heloniopsis orientalis var. flavida	7	244
233	Heloniopsis orientalis var. flavida	3	41
234	Heloniopsis orientalis var. flavida	3	41
235	Heloniopsis orientalis var. flavida	3	88
236	Heloniopsis orientalis var. flavida	7	271
237	Heloniopsis orientalis var. flavida	5	172
238	Heloniopsis orientalis var. flavida	4	152
239	Heloniopsis orientalis var. flavida	3	102
240	Heloniopsis orientalis var. flavida	4	85
241	Heloniopsis orientalis var. flavida	4	160
242	Heloniopsis orientalis var. flavida	3	90
243	Heloniopsis orientalis var. flavida	3	98
244	Heloniopsis orientalis var. flavida	4	155
245	Heloniopsis orientalis var. flavida	4	155
246	Heloniopsis orientalis var. flavida	4	155
247	Heloniopsis orientalis var. flavida	3	102
248	Heloniopsis orientalis var. flavida	3	83
249	Heloniopsis orientalis var. flavida	4	157
250	Heloniopsis orientalis var. flavida	4	157
251	Heloniopsis orientalis var. flavida	4	150
252	Heloniopsis orientalis var. flavida	4	150
253	Heloniopsis orientalis var. flavida	4	150
254	Heloniopsis orientalis var. flavida	11	138
255	Heloniopsis orientalis var. flavida	4	135

256	Heloniopsis orientalis var. flavida	5	196
257	Heloniopsis orientalis var. flavida	10	196
258	Heloniopsis orientalis var. flavida	8	235
259	Heloniopsis orientalis var. flavida	3	56
260	Heloniopsis orientalis var. flavida	3	88
261	Heloniopsis orientalis var. flavida	5	110
262	Heloniopsis orientalis var. orientalis	5	161
263	Heloniopsis orientalis var. orientalis	4	143
264	Heloniopsis orientalis var. orientalis	4	142
265	Heloniopsis orientalis var. orientalis	7	158
266	Heloniopsis orientalis var. orientalis	7	190
267	Heloniopsis orientalis var. orientalis	2	68
268	Heloniopsis orientalis var. orientalis	2	80
269	Heloniopsis orientalis var. orientalis	4	82
270	Heloniopsis orientalis var. orientalis	5	133
271	Heloniopsis orientalis var. orientalis	5	130
272	Heloniopsis orientalis var. orientalis	6	153
273	Heloniopsis orientalis var. orientalis	4	106
274	Heloniopsis orientalis var. orientalis	4	125
275	Heloniopsis orientalis var. orientalis	4	74
276	Heloniopsis orientalis var. orientalis	4	114
277	Heloniopsis orientalis var. orientalis	4	144
278	Heloniopsis orientalis var. orientalis	4	138
279	Heloniopsis orientalis var. orientalis	4	131
280	Heloniopsis orientalis var. orientalis	5	136
281	Heloniopsis orientalis var. orientalis	6	175
282	Heloniopsis orientalis var. orientalis	4	142
283	Heloniopsis orientalis var. orientalis	5	142
284	Heloniopsis orientalis var. orientalis	4	143
285	Heloniopsis orientalis var. orientalis	5	171
286	Heloniopsis orientalis var. orientalis	5	140
287	Heloniopsis orientalis var. orientalis	6	195
288	Heloniopsis orientalis var. orientalis	5	165
289	Heloniopsis orientalis var. orientalis	6	197
290	Heloniopsis orientalis var. orientalis	4	120
291	Heloniopsis orientalis var. orientalis	5	151
292	Heloniopsis orientalis var. orientalis	5	154
293	Heloniopsis orientalis var. orientalis	6	165
294	Heloniopsis orientalis var. orientalis	4	110
295	Heloniopsis orientalis var. orientalis	4	124
296	Heloniopsis orientalis var. orientalis	4	137
297	Heloniopsis orientalis var. orientalis	5	173
298	Heloniopsis orientalis var. orientalis	5	181

299	Heloniopsis orientalis var. orientalis	5	170
300	Heloniopsis orientalis var. orientalis	5	182
301	Heloniopsis orientalis var. orientalis	6	226
302	Heloniopsis orientalis var. orientalis	4	123
303	Heloniopsis orientalis var. orientalis	4	136
304	Heloniopsis orientalis var. orientalis	4	127
305	Heloniopsis orientalis var. orientalis	4	131
306	Heloniopsis orientalis var. orientalis	4	128
307	Heloniopsis orientalis var. orientalis	5	181
308	Heloniopsis orientalis var. orientalis	4	138
309	Heloniopsis orientalis var. orientalis	5	164
310	Heloniopsis orientalis var. orientalis	5	180
311	Heloniopsis orientalis var. orientalis	5	142
312	Heloniopsis orientalis var. orientalis	6	183
313	Heloniopsis orientalis var. orientalis	7	246
314	Heloniopsis orientalis var. orientalis	4	143
315	Heloniopsis orientalis var. orientalis	4	139
316	Heloniopsis orientalis var. orientalis	5	184
317	Heloniopsis orientalis var. orientalis	5	176
318	Heloniopsis orientalis var. orientalis	5	171
319	Heloniopsis orientalis var. orientalis	5	157
320	Heloniopsis orientalis var. orientalis	6	223
321	Heloniopsis orientalis var. orientalis	6	154
322	Heloniopsis orientalis var. orientalis	2	35
323	Heloniopsis orientalis var. orientalis	4	116
324	Heloniopsis orientalis var. orientalis	6	208
325	Heloniopsis orientalis var. orientalis	5	164
326	Heloniopsis orientalis var. orientalis	6	175
327	Heloniopsis orientalis var. orientalis	4	142
328	Heloniopsis orientalis var. orientalis	4	98
329	Heloniopsis orientalis var. orientalis	4	98
330	Heloniopsis orientalis var. orientalis	4	119
331	Heloniopsis orientalis var. orientalis	6	221
332	Heloniopsis orientalis var. orientalis	4	146
333	Heloniopsis orientalis var. orientalis	4	146
334	Heloniopsis orientalis var. orientalis	4	130
335	Heloniopsis orientalis var. orientalis	6	135
336	Heloniopsis orientalis var. orientalis	7	178
337	Heloniopsis orientalis var. orientalis	5	134
338	Heloniopsis orientalis var. orientalis	5	170
339	Heloniopsis orientalis var. orientalis	5	170
340	Heloniopsis orientalis var. orientalis	5	170
341	Heloniopsis orientalis var. orientalis	6	202

342	Heloniopsis orientalis var. orientalis	5	182
343	Heloniopsis orientalis var. orientalis	5	141
344	Heloniopsis orientalis var. orientalis	3	40
345	Heloniopsis orientalis var. orientalis	6	170
346	Heloniopsis orientalis var. orientalis	5	143
347	Heloniopsis orientalis var. orientalis	5	128
348	Heloniopsis orientalis var. orientalis	5	128
349	Heloniopsis orientalis var. orientalis	6	170
350	Heloniopsis orientalis var. orientalis	8	223
351	Heloniopsis orientalis var. orientalis	3	40
352	Heloniopsis orientalis var. orientalis	3	40
353	Heloniopsis orientalis var. orientalis	4	134
354	Heloniopsis orientalis var. orientalis	7	225
355	Heloniopsis orientalis var. orientalis	5	97
356	Heloniopsis orientalis var. orientalis	5	157
357	Heloniopsis orientalis var. orientalis	5	157
358	Heloniopsis orientalis var. orientalis	5	189
359	Heloniopsis orientalis var. orientalis	8	180
360	Heloniopsis orientalis var. orientalis	8	120
361	Heloniopsis orientalis var. orientalis	5	140
362	Heloniopsis orientalis var. orientalis	8	250
363	Heloniopsis orientalis var. orientalis	6	186
364	Heloniopsis orientalis var. orientalis	1	39
365	Heloniopsis orientalis var. orientalis	5	157
366	Heloniopsis orientalis var. orientalis	7	85
367	Heloniopsis orientalis var. orientalis	5	110
368	Heloniopsis orientalis var. orientalis	6	145
369	Heloniopsis orientalis var. orientalis	7	173
370	Heloniopsis orientalis var. orientalis	8	117
371	Heloniopsis orientalis var. orientalis	7	142
372	Heloniopsis orientalis var. orientalis	7	142
373	Heloniopsis orientalis var. orientalis	7	177
374	Heloniopsis orientalis var. orientalis	7	142
375	Heloniopsis orientalis var. orientalis	4	56
376	Heloniopsis orientalis var. orientalis	5	172
377	Heloniopsis orientalis var. orientalis	7	160
378	Heloniopsis orientalis var. orientalis	7	160
379	Heloniopsis orientalis var. orientalis	8	203
380	Heloniopsis orientalis var. orientalis	4	110
381	Heloniopsis orientalis var. orientalis	4	110
382	Heloniopsis orientalis var. orientalis	7	105
383	Heloniopsis sp.	1	91
384	Heloniopsis sp.	1	79

385	Heloniopsis sp.	1	85
386	Heloniopsis sp.	1	93
387	Heloniopsis sp.	2	100
388	Heloniopsis sp.	2	86
389	Heloniopsis sp.	2	93
390	Heloniopsis sp.	2	100
391	Heloniopsis tubiflora	4	59
392	Heloniopsis tubiflora	4	108
393	Heloniopsis tubiflora	4	47
394	Heloniopsis tubiflora	5	109
395	Heloniopsis tubiflora	5	160
396	Heloniopsis tubiflora	5	97
397	Ypsilandra thibetica	3	123
398	Ypsilandra thibetica	3	121
399	Ypsilandra thibetica	3	123
400	Ypsilandra thibetica	3	123
401	Ypsilandra thibetica	3	122
402	Ypsilandra thibetica	3	122
403	Ypsilandra thibetica	3	122
404	Ypsilandra thibetica	3	137
405	Ypsilandra thibetica	3	137
406	Ypsilandra thibetica	3	137
407	Ypsilandra thibetica	3	137
408	Ypsilandra thibetica	3	122
409	Ypsilandra thibetica	3	122
410	Ypsilandra thibetica	3	122
411	Ypsilandra thibetica	3	109
412	Ypsilandra thibetica	3	108
413	Ypsilandra thibetica	3	108
414	Ypsilandra thibetica	3	126
415	Ypsilandra thibetica	3	108
416	Ypsilandra thibetica	3	108
417	Ypsilandra thibetica	3	108
418	Ypsilandra thibetica	3	108
419	Ypsilandra thibetica	3	117
420	Ypsilandra thibetica	3	117
421	Ypsilandra thibetica	3	106
422	Ypsilandra thibetica	3	122
423	Ypsilandra thibetica	3	45
424	Ypsilandra thibetica	3	110
425	Ypsilandra thibetica	3	106
426	Ypsilandra thibetica	3	122
427	Ypsilandra thibetica	3	110

428	Ypsilandra thibetica	3	106
429	Ypsilandra thibetica	3	110
430	Ypsilandra thibetica	3	125
431	Ypsilandra thibetica	3	114
432	Ypsilandra thibetica	3	114
433	Ypsilandra thibetica	3	124
434	Ypsilandra thibetica	3	100
435	Ypsilandra thibetica	3	108
436	Ypsilandra thibetica	3	108
437	Ypsilandra thibetica	3	108
438	Ypsilandra thibetica	3	108
439	Ypsilandra thibetica	3	108
440	Ypsilandra thibetica	3	117
441	Ypsilandra thibetica	3	108
442	Ypsilandra thibetica	3	108
443	Ypsilandra thibetica	3	108
444	Ypsilandra thibetica	3	126
445	Ypsilandra thibetica	3	122
446	Ypsilandra thibetica	3	147
447	Ypsilandra thibetica	3	147
448	Ypsilandra thibetica	3	122
449	Ypsilandra thibetica	3	122
450	Ypsilandra thibetica	3	147
451	Ypsilandra thibetica	3	138
452	Ypsilandra thibetica	3	124
453	Ypsilandra thibetica	3	124
454	Ypsilandra thibetica	3	124
455	Ypsilandra thibetica	3	124
456	Ypsilandra thibetica	3	124
457	Ypsilandra thibetica	3	110
458	Ypsilandra thibetica	3	67
459	Ypsilandra thibetica	3	108
460	Ypsilandra thibetica	3	108
461	Ypsilandra thibetica	3	108
462	Ypsilandra thibetica	3	108
463	Ypsilandra thibetica	3	108
464	Ypsilandra thibetica	3	-57
465	Ypsilandra thibetica	3	52
466	Ypsilandra thibetica	3	141
467	Ypsilandra thibetica	3	110
468	Ypsilandra thibetica	3	110
469	Ypsilandra thibetica	3	110
470	Ypsilandra thibetica	3	110

471	Ypsilandra thibetica	3	141
472	Ypsilandra thibetica	3	110
473	Ypsilandra thibetica	3	110
474	Ypsilandra thibetica	3	110
475	Ypsilandra thibetica	3	110
476	Ypsilandra thibetica	3	110
477	Ypsilandra thibetica	3	110
478	Ypsilandra thibetica	3	110
479	Ypsilandra thibetica	3	110
480	Ypsilandra thibetica	3	127
481	Ypsilandra thibetica	3	127
482	Ypsilandra thibetica	3	110
483	Ypsilandra thibetica	3	110
484	Ypsilandra thibetica	3	150
485	Ypsilandra thibetica	3	126
486	Ypsilandra thibetica	3	123
487	Ypsilandra thibetica	3	125
488	Ypsilandra thibetica	3	122
489	Ypsilandra thibetica	3	125
490	Ypsilandra thibetica	3	122
491	Ypsilandra thibetica	3	122
492	Ypsilandra thibetica	3	122
493	Ypsilandra thibetica	3	122
494	Ypsilandra thibetica	3	122
495	Ypsilandra thibetica	3	122
496	Ypsilandra thibetica	3	122
497	Ypsilandra thibetica	3	118
498	Ypsilandra thibetica	3	125
499	Ypsilandra thibetica	3	122
500	Ypsilandra thibetica	3	122
501	Ypsilandra thibetica	3	122
502	Ypsilandra thibetica	3	126
503	Ypsilandra thibetica	3	126
504	Ypsilandra thibetica	3	137
505	Ypsilandra thibetica	3	110
506	Ypsilandra thibetica	3	126
507	Ypsilandra thibetica	3	110
508	Ypsilandra thibetica	3	110
509	Ypsilandra thibetica	3	126
510	Ypsilandra thibetica	3	126
511	Ypsilandra thibetica	3	126
512	Ypsilandra thibetica	4	175
513	Ypsilandra thibetica	4	183

514	Ypsilandra thibetica	4	173
515	Ypsilandra thibetica	4	173
516	Ypsilandra thibetica	4	170
517	Ypsilandra thibetica	4	170
518	Ypsilandra thibetica	4	170
519	Ypsilandra thibetica	4	196
520	Ypsilandra thibetica	4	196
521	Ypsilandra thibetica	4	196
522	Ypsilandra thibetica	4	196
523	Ypsilandra thibetica	4	170
524	Ypsilandra thibetica	4	170
525	Ypsilandra thibetica	4	170
526	Ypsilandra thibetica	4	154
527	Ypsilandra thibetica	4	158
528	Ypsilandra thibetica	4	158
529	Ypsilandra thibetica	4	176
530	Ypsilandra thibetica	4	158
531	Ypsilandra thibetica	4	158
532	Ypsilandra thibetica	4	158
533	Ypsilandra thibetica	4	158
534	Ypsilandra thibetica	4	172
535	Ypsilandra thibetica	4	172
536	Ypsilandra thibetica	4	154
537	Ypsilandra thibetica	4	170
538	Ypsilandra thibetica	4	86
539	Ypsilandra thibetica	4	159
540	Ypsilandra thibetica	4	154
541	Ypsilandra thibetica	4	170
542	Ypsilandra thibetica	4	159
543	Ypsilandra thibetica	4	154
544	Ypsilandra thibetica	4	159
545	Ypsilandra thibetica	4	186
546	Ypsilandra thibetica	4	164
547	Ypsilandra thibetica	4	164
548	Ypsilandra thibetica	4	172
549	Ypsilandra thibetica	4	148
550	Ypsilandra thibetica	4	158
551	Ypsilandra thibetica	4	158
552	Ypsilandra thibetica	4	158
553	Ypsilandra thibetica	4	158
554	Ypsilandra thibetica	4	158
555	Ypsilandra thibetica	4	172
556	Ypsilandra thibetica	4	158

557	Ypsilandra thibetica	4	158
558	Ypsilandra thibetica	4	158
559	Ypsilandra thibetica	4	187
560	Ypsilandra thibetica	4	170
561	Ypsilandra thibetica	4	203
562	Ypsilandra thibetica	4	203
563	Ypsilandra thibetica	4	170
564	Ypsilandra thibetica	4	170
565	Ypsilandra thibetica	4	203
566	Ypsilandra thibetica	4	196
567	Ypsilandra thibetica	4	172
568	Ypsilandra thibetica	4	172
569	Ypsilandra thibetica	4	172
570	Ypsilandra thibetica	4	172
571	Ypsilandra thibetica	4	172
572	Ypsilandra thibetica	4	159
573	Ypsilandra thibetica	4	115
574	Ypsilandra thibetica	4	158
575	Ypsilandra thibetica	4	158
576	Ypsilandra thibetica	4	158
577	Ypsilandra thibetica	4	158
578	Ypsilandra thibetica	4	158
579	Ypsilandra thibetica	4	-16
580	Ypsilandra thibetica	4	104
581	Ypsilandra thibetica	4	201
582	Ypsilandra thibetica	4	159
583	Ypsilandra thibetica	4	159
584	Ypsilandra thibetica	4	159
585	Ypsilandra thibetica	4	159
586	Ypsilandra thibetica	4	201
587	Ypsilandra thibetica	4	159
588	Ypsilandra thibetica	4	159
589	Ypsilandra thibetica	4	159
590	Ypsilandra thibetica	4	159
591	Ypsilandra thibetica	4	159
592	Ypsilandra thibetica	4	159
593	Ypsilandra thibetica	4	159
594	Ypsilandra thibetica	4	159
595	Ypsilandra thibetica	4	178
596	Ypsilandra thibetica	4	178
597	Ypsilandra thibetica	4	159
598	Ypsilandra thibetica	4	159
599	Ypsilandra thibetica	4	205

600	Ypsilandra thibetica	4	176
601	Ypsilandra thibetica	4	171
602	Ypsilandra thibetica	4	185
603	Ypsilandra thibetica	4	170
604	Ypsilandra thibetica	4	185
605	Ypsilandra thibetica	4	170
606	Ypsilandra thibetica	4	170
607	Ypsilandra thibetica	4	170
608	Ypsilandra thibetica	4	170
609	Ypsilandra thibetica	4	170
610	Ypsilandra thibetica	4	170
611	Ypsilandra thibetica	4	170
612	Ypsilandra thibetica	4	166
613	Ypsilandra thibetica	4	185
614	Ypsilandra thibetica	4	170
615	Ypsilandra thibetica	4	170
616	Ypsilandra thibetica	4	170
617	Ypsilandra thibetica	4	176
618	Ypsilandra thibetica	4	176
619	Ypsilandra thibetica	4	187
620	Ypsilandra thibetica	4	159
621	Ypsilandra thibetica	4	176
622	Ypsilandra thibetica	4	159
623	Ypsilandra thibetica	4	159
624	Ypsilandra thibetica	4	176
625	Ypsilandra thibetica	4	176
626	Ypsilandra thibetica	4	176
627	Ypsilandra yunnanensis	6	126
628	Ypsilandra yunnanensis	6	120
629	Ypsilandra yunnanensis	6	126
630	Ypsilandra yunnanensis	6	120
631	Ypsilandra yunnanensis	6	120
632	Ypsilandra yunnanensis	6	118
633	Ypsilandra yunnanensis	6	120
634	Ypsilandra yunnanensis	6	116
635	Ypsilandra yunnanensis	6	120
636	Ypsilandra yunnanensis	6	137
637	Ypsilandra yunnanensis	6	120
638	Ypsilandra yunnanensis	6	120
639	Ypsilandra yunnanensis	6	120
640	Ypsilandra yunnanensis	6	117
641	Ypsilandra yunnanensis	6	140
642	Ypsilandra yunnanensis	6	117

643	Ypsilandra yunnanensis	6	140
644	Ypsilandra yunnanensis	6	140
645	Ypsilandra yunnanensis	6	101
646	Ypsilandra yunnanensis	6	186
647	Ypsilandra yunnanensis	6	186
648	Ypsilandra yunnanensis	6	146
649	Ypsilandra yunnanensis	6	146
650	Ypsilandra yunnanensis	6	146
651	Ypsilandra yunnanensis	6	146
652	Ypsilandra yunnanensis	6	146
653	Ypsilandra yunnanensis	6	209
654	Ypsilandra yunnanensis	6	209
655	Ypsilandra yunnanensis	6	209
656	Ypsilandra yunnanensis	6	211
657	Ypsilandra yunnanensis	6	138
658	Ypsilandra yunnanensis	6	137
659	Ypsilandra yunnanensis	6	137
660	Ypsilandra yunnanensis	6	137
661	Ypsilandra yunnanensis	6	209
662	Ypsilandra yunnanensis	6	137
663	Ypsilandra yunnanensis	6	137
664	Ypsilandra yunnanensis	6	209
665	Ypsilandra yunnanensis	6	137
666	Ypsilandra yunnanensis	6	137
667	Ypsilandra yunnanensis	6	137
668	Ypsilandra yunnanensis	6	137
669	Ypsilandra yunnanensis	6	137
670	Ypsilandra yunnanensis	6	137
671	Ypsilandra yunnanensis	6	137
672	Ypsilandra yunnanensis	6	209
673	Ypsilandra yunnanensis	6	209
674	Ypsilandra yunnanensis	6	209
675	Ypsilandra yunnanensis	6	209
676	Ypsilandra yunnanensis	6	209
677	Ypsilandra yunnanensis	6	209
678	Ypsilandra yunnanensis	6	209
679	Ypsilandra yunnanensis	6	137
680	Ypsilandra yunnanensis	6	137
681	Ypsilandra yunnanensis	6	209
682	Ypsilandra yunnanensis	6	187
683	Ypsilandra yunnanensis	6	122
684	Ypsilandra yunnanensis	7	131
685	Ypsilandra yunnanensis	7	126

686	Ypsilandra yunnanensis	7	131
687	Ypsilandra yunnanensis	7	124
688	Ypsilandra yunnanensis	7	124
689	Ypsilandra yunnanensis	7	122
690	Ypsilandra yunnanensis	7	124
691	Ypsilandra yunnanensis	7	120
692	Ypsilandra yunnanensis	7	124
693	Ypsilandra yunnanensis	7	140
694	Ypsilandra yunnanensis	7	124
695	Ypsilandra yunnanensis	7	125
696	Ypsilandra yunnanensis	7	125
697	Ypsilandra yunnanensis	7	122
698	Ypsilandra yunnanensis	7	145
699	Ypsilandra yunnanensis	7	122
700	Ypsilandra yunnanensis	7	145
701	Ypsilandra yunnanensis	7	145
702	Ypsilandra yunnanensis	7	112
703	Ypsilandra yunnanensis	7	188
704	Ypsilandra yunnanensis	7	188
705	Ypsilandra yunnanensis	7	162
706	Ypsilandra yunnanensis	7	162
707	Ypsilandra yunnanensis	7	162
708	Ypsilandra yunnanensis	7	162
709	Ypsilandra yunnanensis	7	162
710	Ypsilandra yunnanensis	7	214
711	Ypsilandra yunnanensis	7	214
712	Ypsilandra yunnanensis	7	214
713	Ypsilandra yunnanensis	7	218
714	Ypsilandra yunnanensis	7	147
715	Ypsilandra yunnanensis	7	143
716	Ypsilandra yunnanensis	7	143
717	Ypsilandra yunnanensis	7	143
718	Ypsilandra yunnanensis	7	214
719	Ypsilandra yunnanensis	7	143
720	Ypsilandra yunnanensis	7	143
721	Ypsilandra yunnanensis	7	214
722	Ypsilandra yunnanensis	7	143
723	Ypsilandra yunnanensis	7	143
724	Ypsilandra yunnanensis	7	143
725	Ypsilandra yunnanensis	7	143
726	Ypsilandra yunnanensis	7	143
727	Ypsilandra yunnanensis	7	143
728	Ypsilandra yunnanensis	7	143

729	Ypsilandra yunnanensis	7	214
730	Ypsilandra yunnanensis	7	214
731	Ypsilandra yunnanensis	7	214
732	Ypsilandra yunnanensis	7	214
733	Ypsilandra yunnanensis	7	214
734	Ypsilandra yunnanensis	7	214
735	Ypsilandra yunnanensis	7	214
736	Ypsilandra yunnanensis	7	143
737	Ypsilandra yunnanensis	7	143
738	Ypsilandra yunnanensis	7	214
739	Ypsilandra yunnanensis	7	206
740	Ypsilandra yunnanensis	7	131
741	Heloniopsis umbellata	2	116
742	Heloniopsis umbellata	3	121
743	Heloniopsis umbellata	2	78
744	Heloniopsis umbellata	4	116
745	Heloniopsis umbellata	4	85
746	Heloniopsis umbellata	1	111
747	Heloniopsis umbellata	1	111
748	Heloniopsis umbellata	2	70
749	Heloniopsis umbellata	2	116
750	Heloniopsis umbellata	2	116
751	Heloniopsis umbellata	2	91
752	Heloniopsis umbellata	2	105
753	Heloniopsis umbellata	2	145
754	Heloniopsis umbellata	2	69
755	Heloniopsis umbellata	2	75
756	Heloniopsis umbellata	2	93
757	Heloniopsis umbellata	3	121
758	Heloniopsis umbellata	2	112
759	Heloniopsis umbellata	3	83
760	Heloniopsis umbellata	2	68
761	Heloniopsis umbellata	2	110
762	Heloniopsis umbellata	3	87
763	Heloniopsis umbellata	3	87
764	Heloniopsis umbellata	2	124
765	Heloniopsis umbellata	1	53
766	Heloniopsis umbellata	1	81
767	Heloniopsis umbellata	1	81
768	Heloniopsis umbellata	12	140
769	Heloniopsis umbellata	1	110
770	Heloniopsis umbellata	1	85
771	Heloniopsis umbellata	1	124

772	Heloniopsis umbellata	1	102
773	Heloniopsis umbellata	1	83
774	Heloniopsis umbellata	1	112
775	Heloniopsis umbellata	2	114
776	Heloniopsis umbellata	2	114
777	Heloniopsis umbellata	1	107
778	Heloniopsis umbellata	2	110
779	Heloniopsis umbellata	1	83
780	Heloniopsis umbellata	2	90
781	Heloniopsis umbellata	2	112
782	Heloniopsis umbellata	2	136
783	Heloniopsis umbellata	2	112
784	Heloniopsis umbellata	2	105
785	Heloniopsis umbellata	2	68
786	Heloniopsis umbellata	2	110
787	Heloniopsis umbellata	2	104
788	Heloniopsis umbellata	2	104
789	Heloniopsis umbellata	2	104
790	Heloniopsis umbellata	2	104
791	Heloniopsis umbellata	2	86
792	Heloniopsis umbellata	2	104
793	Heloniopsis umbellata	2	104
794	Heloniopsis umbellata	2	114
795	Heloniopsis umbellata	2	148
796	Heloniopsis umbellata	2	69
797	Heloniopsis umbellata	2	70
798	Heloniopsis umbellata	3	87
799	Heloniopsis umbellata	3	79
800	Heloniopsis umbellata	3	79
801	Heloniopsis umbellata	3	79
802	Heloniopsis umbellata	3	88
803	Heloniopsis umbellata	3	93
804	Heloniopsis umbellata	3	151
805	Heloniopsis umbellata	3	86
806	Heloniopsis umbellata	3	70
807	Heloniopsis umbellata	2	104
808	Heloniopsis umbellata	3	86
809	Heloniopsis umbellata	2	114
810	Heloniopsis umbellata	2	114
811	Heloniopsis umbellata	3	96
812	Heloniopsis tubiflora	4	86
813	Heloniopsis tubiflora	4	56
814	Heloniopsis tubiflora	4	56

815	Heloniopsis tubiflora	4	55
816	Heloniopsis tubiflora	4	51
817	Heloniopsis tubiflora	4	51
818	Heloniopsis tubiflora	4	68
819	Heloniopsis tubiflora	4	93
820	Heloniopsis tubiflora	4	60
821	Heloniopsis tubiflora	4	86
822	Heloniopsis tubiflora	4	59
823	Heloniopsis tubiflora	4	108
824	Heloniopsis tubiflora	4	47
825	Heloniopsis tubiflora	5	137
826	Heloniopsis tubiflora	5	105
827	Heloniopsis tubiflora	5	105
828	Heloniopsis tubiflora	5	107
829	Heloniopsis tubiflora	5	104
830	Heloniopsis tubiflora	5	104
831	Heloniopsis tubiflora	5	117
832	Heloniopsis tubiflora	5	139
833	Heloniopsis tubiflora	5	113
834	Heloniopsis tubiflora	5	143
835	Heloniopsis tubiflora	5	109
836	Heloniopsis tubiflora	5	160
837	Heloniopsis tubiflora	5	97
838	Helonias bullata	4	116
839	Helonias bullata	4	117
840	Helonias bullata	4	111
841	Helonias bullata	4	123
842	Helonias bullata	4	110
843	Helonias bullata	4	119
844	Helonias bullata	4	119
845	Helonias bullata	4	150
846	Helonias bullata	4	81
847	Helonias bullata	4	98
848	Helonias bullata	4	105
849	Helonias bullata	4	96
850	Helonias bullata	4	136
851	Helonias bullata	4	130
852	Helonias bullata	4	120
853	Helonias bullata	4	117
854	Helonias bullata	4	126
855	Helonias bullata	4	120
856	Helonias bullata	4	125
857	Helonias bullata	4	130

858	Helonias bullata	4	154
859	Helonias bullata	4	123
860	Helonias bullata	4	130
861	Helonias bullata	4	131
862	Helonias bullata	4	113
863	Helonias bullata	4	132
864	Helonias bullata	4	108
865	Helonias bullata	4	133
866	Helonias bullata	4	107
867	Helonias bullata	4	83
868	Helonias bullata	4	135
869	Helonias bullata	4	137
870	Helonias bullata	4	123
871	Helonias bullata	4	123
872	Helonias bullata	4	109
873	Helonias bullata	4	116
874	Helonias bullata	4	118
875	Helonias bullata	4	107
876	Helonias bullata	4	97
877	Helonias bullata	4	97
878	Helonias bullata	4	96
879	Helonias bullata	4	116
880	Helonias bullata	4	97
881	Helonias bullata	4	97
882	Helonias bullata	4	104
883	Helonias bullata	4	114
884	Helonias bullata	4	96
885	Helonias bullata	4	96
886	Helonias bullata	4	97
887	Helonias bullata	4	114
888	Helonias bullata	4	123
889	Helonias bullata	4	119
890	Helonias bullata	4	119
891	Helonias bullata	4	117
892	Helonias bullata	4	111
893	Helonias bullata	4	117
894	Helonias bullata	4	107
895	Helonias bullata	4	109
896	Helonias bullata	5	161
897	Helonias bullata	5	161
898	Helonias bullata	5	156
899	Helonias bullata	5	174
900	Helonias bullata	5	163

901	Helonias bullata	5	164
902	Helonias bullata	5	164
903	Helonias bullata	5	195
904	Helonias bullata	5	136
905	Helonias bullata	5	143
906	Helonias bullata	5	150
907	Helonias bullata	5	141
908	Helonias bullata	5	183
909	Helonias bullata	5	175
910	Helonias bullata	5	172
911	Helonias bullata	5	161
912	Helonias bullata	5	170
913	Helonias bullata	5	173
914	Helonias bullata	5	170
915	Helonias bullata	5	175
916	Helonias bullata	5	199
917	Helonias bullata	5	168
918	Helonias bullata	5	184
919	Helonias bullata	5	176
920	Helonias bullata	5	166
921	Helonias bullata	5	185
922	Helonias bullata	5	162
923	Helonias bullata	5	186
924	Helonias bullata	5	160
925	Helonias bullata	5	127
926	Helonias bullata	5	183
927	Helonias bullata	5	184
928	Helonias bullata	5	169
929	Helonias bullata	5	169
930	Helonias bullata	5	162
931	Helonias bullata	5	171
932	Helonias bullata	5	172
933	Helonias bullata	5	160
934	Helonias bullata	5	154
935	Helonias bullata	5	154
936	Helonias bullata	5	153
937	Helonias bullata	5	170
938	Helonias bullata	5	154
939	Helonias bullata	5	154
940	Helonias bullata	5	159
941	Helonias bullata	5	167
942	Helonias bullata	5	153
943	Helonias bullata	5	153

944	Helonias bullata	5	154
945	Helonias bullata	5	167
946	Helonias bullata	5	178
947	Helonias bullata	5	174
948	Helonias bullata	5	174
949	Helonias bullata	5	172
950	Helonias bullata	5	166
951	Helonias bullata	5	172
952	Helonias bullata	5	160
953	Helonias bullata	5	164
954	Heloniopsis kawanoi	4	179
955	Heloniopsis kawanoi	4	225
956	Heloniopsis kawanoi	4	205
957	Heloniopsis kawanoi	4	215
958	Heloniopsis kawanoi	4	224
959	Heloniopsis kawanoi	4	212
960	Heloniopsis kawanoi	4	211
961	Heloniopsis kawanoi	5	207
962	Heloniopsis kawanoi	5	248
963	Heloniopsis kawanoi	5	231
964	Heloniopsis kawanoi	5	239
965	Heloniopsis kawanoi	5	247
966	Heloniopsis kawanoi	5	236
967	Heloniopsis kawanoi	5	235
968	Heloniopsis leucantha	1	164
969	Heloniopsis leucantha	1	166
970	Heloniopsis leucantha	1	183
971	Heloniopsis leucantha	1	184
972	Heloniopsis leucantha	2	165
973	Heloniopsis leucantha	2	167
974	Heloniopsis leucantha	2	187
975	Heloniopsis leucantha	2	188
976	Heloniopsis sp.	1	91
977	Heloniopsis sp.	1	147
978	Heloniopsis sp.	1	85
979	Heloniopsis sp.	1	93
980	Heloniopsis sp.	2	100
981	Heloniopsis sp.	2	152
982	Heloniopsis sp.	2	93
983	Heloniopsis sp.	2	100

Supplement table S9. Sample number of Heloniadeae taxa for anthesis temperature analysis

Scientific Name	Number
Helonias bullata	58
Helonias kawanoi	7
Heloniopsis koreana	32
Heloniopsis leucantha	4
Heloniopsis orientalis var. *breviscapa*	85
Heloniopsis orientalis var. *flavida*	144
Heloniopsis orientalis var. *orientalis*	121
Heloniopsis sp.	4
Heloniopsis tubiflora	13
Ypsilandra thibetica	115
Ypsilandra yunnanensis	57
**total**	**640**
